# Diversity and conservation of legumes in the Gran Chaco and biogeograpical inferences

**DOI:** 10.1371/journal.pone.0220151

**Published:** 2019-08-14

**Authors:** Matías Morales, Luis Oakley, Angela L. B. Sartori, Virginia Y. Mogni, Margoth Atahuachi, Ricardo O. Vanni, Renée H. Fortunato, Darién E. Prado

**Affiliations:** 1 Instituto de Recursos Biológicos (CIRN–CNIA, INTA). Las Cabañas y Los Reseros s.n. Hurlingham (1686), Argentina; 2 Consejo Nacional de Investigaciones Científicas y Técnicas (CONICET). Buenos Aires. Argentina; 3 Facultad de Agronomía y Cs. Agroalimentarias, Universidad de Morón, Cabildo, Morón, Argentina; 4 Facultad de Ciencias Agrarias, Universidad Nacional de Rosario. Campo Experimental Villarino, CC Nº 14, S2125ZAA, Zavalla, Santa Fe, Argentina; 5 Red List Authority Coordinator for the Temperate South American Plant Specialist Groups -International Union for Conservation of Nature (IUCN), Cambridge, United Kingdom; 6 Universidade Federal de Mato Grosso do Sul, Instituto de Biociências, Laboratório de Sistemática Vegetal, Cidade Universitária, s/n, C.P. 549, CEP, Campo Grande, Mato Grosso do Sul, Brasil; 7 Herbario Forestal Nacional M. Cárdenas, Centro de Biodiversidad y Genética, Universidad Mayor de San Simón, Final Jordan este, Casilla, Cochabamba, Bolivia; 8 Instituto de Botánica del Nordeste (IBONE), Casilla de Correo, Corrientes, Argentina; 9 Instituto de Investigaciones en Ciencias Agrarias IICAR (UNR-CONICET), Zavalla, Santa Fe, Argentina; University of Pittsburgh, UNITED STATES

## Abstract

The Gran Chaco is a wide ecologic-geographic region comprising northern Argentina, western Paraguay, southern Bolivia and the southwestern extreme of Brazil. This region exhibits extreme temperatures, annually regular frosts, and sedimentary soils; it has been dramatically threatened by agriculture expansion in recent decades. Therefore, increasing knowledge of plant diversity is critical for conservation purposes. We present a Legume checklist of the Gran Chaco ecoregion including conservation status of its endemic species. Leguminosae is the third most diverse plant family in the Neotropics. Assuming a rigorous spatial definition of the Gran Chaco, we recorded 98 genera, 362 species, and 404 specific and infraspecific taxa. Endemic/typical taxa were 17%, comparable to adjacent tropical plant formations, and they were found in higher percentages in Caesalpinioideae (24%) and Cercidoideae (33%) than Papilionoideae (11%) subfamily. We also analyzed the plant diversity comparing lineages and subregions. The Gran Chaco Legumes are predominantly widespread generalists, or they belong to either Chaco sensu stricto or Neotropical Seasonally Dry Tropical Forest (SDTF) lineages. Though the Humid Chaco registered the highest species richness, Dry Chaco and Sierra Chaco, the most threatrened subregions, exhibited the highest percentages of exclusive and proper Chaco-lineage species. These results suggest that diversification of Legumes has been most relevant in Dry Chaco and Sierra Chaco, probably by their more demanding and harsh environmental conditions limiting the dispersion of generalists or intrusive-invading species. This study is paramount to reach an improved delimitation of the Gran Chaco ecoregion in transitional areas with the SDTF and Cerrado formations. Conservation status is critical in genera of high economic interest, such as *Arachis*, *Mimosa* and *Prosopis*. At least one third of endemic taxa exhibit a critical status of conservation or are endangered, many of them being relevant to inbreeding program or exhibiting multiple economic uses.

## Introduction

There are some controversies about the definition of Chaco. The Gran Chaco ecoregion or Chaco sensu lato is an ample region in South America, defined by geographical, economical and social criteria [1, 2]. This region has been largely studied in terms of plant diversity, vegetation structure and composition [1, 2], particularly for the Argentine sector. The biogeographical Chaco or Chaco *sensu stricto* comprises areas with exclusive or almost exclusive taxa and plant formations.

The ecoregion called Gran Chaco is a large area located in central-southern South America. There are different geographical circumscriptions of this region, but, in general, it comprises northeastern Argentina, central and western Paraguay, southeastern Bolivia and adjacent areas of Brazil [1, 3, 4, 5, 6, 7]. The Gran Chaco is characterized by its subtropical climate with an average annual temperature ca. 18–22°C, frequent frosts in winter and annual precipitation of 500–1200 mm, with minimum extremes of 300 mm in some parts of Bolivian Chaco [5] and the presence of different types of soils but usually saline or alkaline [8].

According to [9], the biogeographical Chaco is part of the Chaquenian Dominium, which comprises a large portion of southern South America, extending from the Caatinga of northeastern Brazil, then south to the Chaco, Espinal, Pampean, Monte and Prepuna provinces. However, in an extensive floristic study of the vegetation of this part of the continent, [10] demonstrated that the Caatinga province is floristically very different from the rest of the Chaquenian Dominium and therefore that it should be excluded from the latter.

The biogeographical Chaco or Chaco *sensu stricto* is based on the presence of communities where the dominant species belong to *Schinopsis* Engl. (communities commonly referred as “quebrachal”), *Prosopis* L. (“algarrobal” and “vinalar”, among others), *Bulnesia* Gay (“palosantal”) and *Stetsonia* Britton and Rose (“cardonal”) [1, 2, 11]. Several Chaco subdivisions have been proposed; for example, [2] partially based in [12, 13, 14], among others, stated the existence of different subregions: the Humid Chaco or Eastern Chaco, dominated mainly by forests of “quebracho colorado chaqueño” (*Schinopsis balansae* Engl.); the Central Chaco, dominated by formations of different species of “quebracho” (*S*. *balansae*, *S*. *lorentzii* (Griseb.) Engl. and *Aspidosperma quebracho-blanco* Schltdl.), and the Dry Chaco or Western Chaco, with formations dominated by *S*. *lorentzii*, *Bulnesia sarmientoi* Lorentz ex Griseb. and *A*. *quebracho-blanco* (the latter case called Arid Chaco). [15] coincides with the presence of formations defined by *A*. *quebracho-blanco* in western areas of Bolivian Chaco, *A*. *triternatum* Rojas Acosta and *Bulnesia sarmientoi* in poorly drained soils and *Schinopsis balansae* in eastern Bolivian Chaco.

Additionally, [1, 2] also re-defined the phytogeographical Chaco by analyzing different phytosociological studies and reconciling different biogeographer’s criteria. Thus, the Chaco *sensu stricto* was defined as the region where the typical Chaco forest formations are present [2], including the forests and savannas of north-central Argentina, southeastern Bolivia and western and central Paraguay, plus a reduced area of southwestern Brazil [16]. The existence of transitional areas where the Chaco formations are present but admixtures with other phytogeographic units have been pointed out [1, 11]; they mainly correspond to parts of the eastern Chaco, extended as a transitional belt along the Paraguay-Paraná fluvial system on its right margin and with some transgressions on the left margin [17].

Later, [18] defined a new vegetation unit in South America, called the Neotropical Seasonally Dry Tropical Forests (more widely known today as 'SDTF'; DRYFLOR 2016). The SDTFs extend from the Caatinga of northeastern Brazil, to the Piedmont forests in the Andean foothills of northwestern Argentina, dry Andean valleys of western Bolivia and finally reaching Peru. This arc of seasonally dry forests gets through the Chiquitanía (located in eastern-central Bolivia and a small area in northern Paraguay), and on some calcareous outcrops of southern Brazil and neighboring Paraguay [9, 19, 20]. The SDTFs show a clear-cut leafless period, as it happens in several species of the genera *Anadenanthera* Speg., *Cordia* L., *Handroanthus* Mattos, *Myracrodruon* Allemão, *Pterogyne* Tul., *Ruprechtia* C.A. Mey., and some particular species of *Mimosa* L. and *Brasiliopuntia* (K. Schum.) A. Berger, among others [18, 19, 21]. On the other hand, [22] and [23] distinguished and separated SDTFs from the Chaco *s*.*s*. forests, basically by their distinct floristic composition and dominant species.

The Gran Chaco ecoregion has experienced drastic changes in land use in recent times, leading to a dramatic deforestation throughout its extension. During the first half of the XX century, the forests of *Schinopsis* spp. were devastated to tannin and sleeper production. From the ending of the XX century to present, the Dry Chaco in particular has been suffering intense deforestation by means of cattle and agriculture expansion. This is more visible in Argentina, where it seems to be related to the introduction of RR cultivars of soybean and non–tillage agricultural systems, the increase of annual precipitation and the high prices of commodities [24]. Contrarily, in the Paraguayan Chaco both deforestation and transformation of land use are more related to cattle expansion [25]. Nevertheless, agriculture expansion in Argentina could be associated to cattle expansion in the adjacent Paraguay, Bolivia and Brazil [26]. Only between 2001 and 2012, more than 7 million hectares were deforested in Chaco [25], presenting one of the highest deforestation rates in the world, ca. 2.2% in areas of Central Argentina from 1969 to 1999 [27], and 1% per year in Paraguayan Chaco, from 1997 to 2012 [26]. Future scenarios suggest strong probabilities of further agriculture expansion in the next decades, especially threatening the Gran Chaco areas currently best preserved in all three countries [28].

Leguminosae Juss. is one of the most diversified families of vascular plants in the world and in the Neotropics [29, 30]. In fact, in the Americas it is the third family in number of species [31]. In South America, there are still scarce extensive studies concerning their diversity and distribution in the large units of vegetation of the subcontinent [20, 32, 33]. The recently published on-line checklist of Brazilian Flora, Lista de Espécies do Brasil [34] provides a good example of a database including biogeographical searches. Other databases provide appropriate tools to analyze the flora of different South American regions, but their criterion is administrative (e.g. [35]).

For example, in the so-called Southern Cone of South America (Argentina, Chile, Paraguay, Uruguay and southern Brazil, as defined in [36], this family is the third in species number (1,365), following Asteraceae (2,523) and Poaceae (1,535). Some of the most diversified genera of the region belong to this family, such as *Adesmia* DC. (ca. 200 species) and *Mimosa* (ca 169 species). Concerning endemism, the Southern Cone states that ca. 45% of the Leguminosae species are endemic [36]. Recently, [33] presented a woody Legumes checklist for the dry tropical vegetation types of eastern South America: Brazilian Savannas (= Cerrado), Seasonal Forests (= Caatinga and Paranaense forests) and Chaco. They found that Chaco had the highest richness of woody species among these tropical regions. However, despite its extension, its level of endemism and overall importance of the rapidly disappearing Gran Chaco ecosystems, there is no comprehensive Legume checklist for this region.

In this work, we present an exhaustive checklist of all Legume taxa of all life forms from the Gran Chaco ecoregion, adopting the criterion of [37], with modifications, for the delimitation of this region and its subregions. The present study also allowed to perform biogeographical inferences about Chaco subregions and lineages of its species. In addition, we present the first advances of Chaquenian Legumes conservation, inferring their status based on the available information from distributional data.

## Material and methods

### Delimitation of the area

We mapped the Gran Chaco ecoregion based on the maps of [37] actualized in [38], integrating Humid Chaco and Dry Chaco. This area extensively coincides with our previous definitions of Chaco ecoregion, and mainly includes the biogeographical Chaco and some transitional areas with SDTF in eastern Paraguay and northeastern Argentina.

### Specimen database

A database containing all Legume taxa present in the Gran Chaco was created. These data were obtained from: 1) Mainly herbarium specimens, whose identification was checked by us; 2) available on-line databases of leguminous specimens and taxa; e.g.: Catálogo de las Plantas Vasculares del Cono Sur [35]; Brazilian Flora Checklist [34], TROPICOS [39] and Species Link [40]. Taxonomic identifications and geographical data of specimens from these databases were checked. At least one specimen per specific and infraspecific taxa from the Gran Chaco and per subregion was used to document the database. In particular cases, when we failed to locate some specimens, the reason of this inclusion or exclusion was adequately explained. Taxa that are typical from other ecoregions with occasional occurrence in border contact areas were not included. The studied specimens were deposited mainly inthe following herbaria: BAB, BOL, CGMS, COCH, COR, CTES, FCQ, HUEFS, ICN, INPA, K, LIL, LPB, MBM, MO, NY, SI, UFMS, UNR, US, and USZ (acronyms according to the Index Herbariorum [41]. Data of representative specimens are cited in the supplementary files (S1 File) and the rest are available in the mentioned public databases. The representative specimens were: 1) for non-endemic taxa of Chaco, one to five specimens collected in the Gran Chaco ecoregion to cleary register the simple presence; 2) for endemic and typical taxa from Gran Chaco, all or almost all specimens from all localities where these taxa were registered.

### Classification of taxa, subregions and lineages converging in the Gran Chaco ecoregion

Taxa of categories from subfamily to form were recorded, including genus, species, subspecies and variety. However, in the statistical analysis only the specific and infraspecific levels were considered. When the analyses included taxa both at specific and infraspecific levels simultaneously, they were computed as follows: form, variety and subspecies were registered as the same entity with the corresponding species.

All taxonomic identifications were adopted according to the more recent taxonomic treatments of the genera in the region (Table 1) and validated with the most recent nomenclatural modifications [35]. We adopted the criterion of The Legume Phylogeny Working Group [30] to classify the species in subfamilies. Thus, we considered six subfamilies: Detarioideae, Dialioideae, Cercidoideae, Duparquetioideae, Caesalpinioideae, and Papilionoideae, instead of the traditional classification in three subfamilies (Mimosoideae, Caesalpinioideae, and Papilionoideae). In the particular case of the genus *Acacia* Mill., given thenomenclatural controversies, we considered it as one genus, *Acacia sensu lato*, coinciding with the criterion of [42], instead of the combination or names within *Acaciella* Britton & Rose [43], *Senegalia* Raf. [44, 45] and *Vachellia* Wight & Arn. [46]. The genus *Caesalpinia* L. was recognised here with its recently segregates genera such as *Arquita* Gagnon, G.P.Lewis & C.E.Hughes, *Cenostigma* Tul., *Erythrostemon* Klotzsch, and *Libidibia* Schltdl. [47]. For the genus *Vigna* was followed the names according to the criterion of [48].

**Table 1:** Essential references for the taxonomic identification of Chaco Legumes.

Subfamily	Genus	Basic taxonomic bibliography
Cercidoideae	*Bauhinia*	[134, 135, 136]
Detarioideae	*Copaifera*	[80, 137]
Detarioideae	*Cynometra*	[80, 138]
Caesalpinioideae	*Caesalpinia*	[47, 80, 92]
Caesalpinioideae	*Chamaecrista*	[91]
Caesalpinioideae	*Cercidium*	[92]
Caesalpinioideae	*Gleditsia*	[80, 92]
Caesalpinioideae	*Hymenaea*	[139]
Caesalpinioideae	*Hoffmanseggia*	[92]
Caesalpinioideae	*Libidibia*	[47, 80, 92]
Caesalpinioideae	*Lophocarpinia*	[80]
Caesalpinioideae	*Parkinsonia*	[80]
Caesalpinioideae	*Peltophorum*	[80, 92]
Caesalpinioideae	*Pterogyne*	[80, 92]
Caesalpinioideae	*Senna*	[91]
Caesalpinioideae	*Stenodrepanum*	[92]
Caesalpinioideae	*Acacia*	[77, 78]
Caesalpinioideae	*Albizia*	[85]
Caesalpinioideae	*Anadenanthera*	[77, 89]
Caesalpinioideae	*Calliandra*	[79]
Caesalpinioideae	*Chloroleucon*	[85]
Caesalpinioideae	*Desmanthus*	[87]
Caesalpinioideae	*Enterolobium*	[80, 85]
Caesalpinioideae	*Inga*	[81, 88]
Caesalpinioideae	*Microlobius*	
Caesalpinioideae	*Mimosa*	[74, 141]
Caesalpinioideae	*Mimoziganthus*	[80]
Caesalpinioideae	*Neptunia*	[80, 84]
Caesalpinioideae	*Parapiptadenia*	[77, 80]
Caesalpinioideae	*Prosopidastrum*	[80, 82]
Caesalpinioideae	*Piptadeniopsis*	[80]
Caesalpinioideae	*Prosopis*	[68, 71]
Caesalpinioideae	*Zapoteca*	[88]
Caesalpinioideae	*Zygia*	[86]
Papilionoideae	*Acosmium*	[133]
Papilionoideae	*Adesmia*	[129]
Papilionoideae	*Aeschynomene*	[103, 107]
Papilionoideae	*Amburana*	[80, 117]
Papilionoideae	*Ancistotropis*	[48, 80]
Papilionoideae	*Apurimacia*	[80]
Papilionoideae	*Arachis*	[80, 124, 125]
Papilionoideae	*Astragalus*	[126, 128]
Papilionoideae	*Calopogonium*	[80]
Papilionoideae	*Camptosema*	[123]
Papilionoideae	*Canavalia*	[80, 121]
Papilionoideae	*Centrosema*	[80, 101]
Papilionoideae	*Chaetocalyx*	[106]
Papilionoideae	*Clitoria*	[80]
Papilionoideae	*Cochliasanthus*	[48]
Papilionoideae	*Collaea*	[80]
Papilionoideae	*Cologania*	[80]
Papilionoideae	*Condylostylis*	[48]
Papilionoideae	*Coursetia*	[80]
Papilionoideae	*Crotalaria*	[80, 112]
Papilionoideae	*Cyclolobium*	[116, 119]
Papilionoideae	*Dalbergia*	[80, 132]
Papilionoideae	*Dalea*	[113]
Papilionoideae	*Desmodium*	[97, 99, 100]
Papilionoideae	*Dioclea*	[80]
Papilionoideae	*Discolobium*	[80, 102. 109]
Papilionoideae	*Dolichopsis*	[123]
Papilionoideae	*Eriosema*	[105]
Papilionoideae	*Erythrina*	[120]
Papilionoideae	*Galactia*	[111]
Papilionoideae	*Geoffroea*	[80]
Papilionoideae	*Helicotropis*	[48]
Papilionoideae	*Holocalyx*	[80]
Papilionoideae	*Indigofera*	[114]
Papilionoideae	*Lathyrus*	[80]
Papilionoideae	*Lonchocarpus*	[114, 118]
Papilionoideae	*Luetzelburgia*	[134]
Papilionoideae	*Lupinus*	[80]
Papilionoideae	*Machaerium*	[80]
Papilionoideae	*Macroptilium*	[98]
Papilionoideae	*Medicago*	[80]
Papilionoideae	*Melilotus*	[80]
Papilionoideae	*Myrocarpus*	[80, 115]
Papilionoideae	*Nissolia*	[80]
Papilionoideae	*Muellera*	[80, 114, 118]
Papilionoideae	*Otholobium*	[80]
Papilionoideae	*Phaseolus*	[123]
Papilionoideae	*Poisonia*	[80]
Papilionoideae	*Poiretia*	[80]
Papilionoideae	*Pterocarpus*	[80]
Papilionoideae	*Rhynchosia*	[95]
Papilionoideae	*Sesbania*	[114]
Papilionoideae	*Stylosanthes*	[104, 108]
Papilionoideae	*Sweetia*	[80]
Papilionoideae	*Tephrosia*	[80]
Papilionoideae	*Trifolium*	[80]
Papilionoideae	*Vicia*	[80, 110]
Papilionoideae	*Vigna*	[48, 123]; Delgado-Salinas et al. 2011
Papilionoideae	*Zornia*	[96]

All taxa were considered under these different distribution criteria: a) lineage; b) distribution in subregions within the Gran Chaco; c) Endemism in the Gran Chaco. In addition, the endemic and typicalspecies of Gran Chaco were classified according to their conservation status. The typical species should be non-endemic from the Gran Chaco, but with the majority of their occurrences within Gran Chaco boundaries. All these classifications are explained in the following paragraphs.

#### Lineage

**Fig 1:**
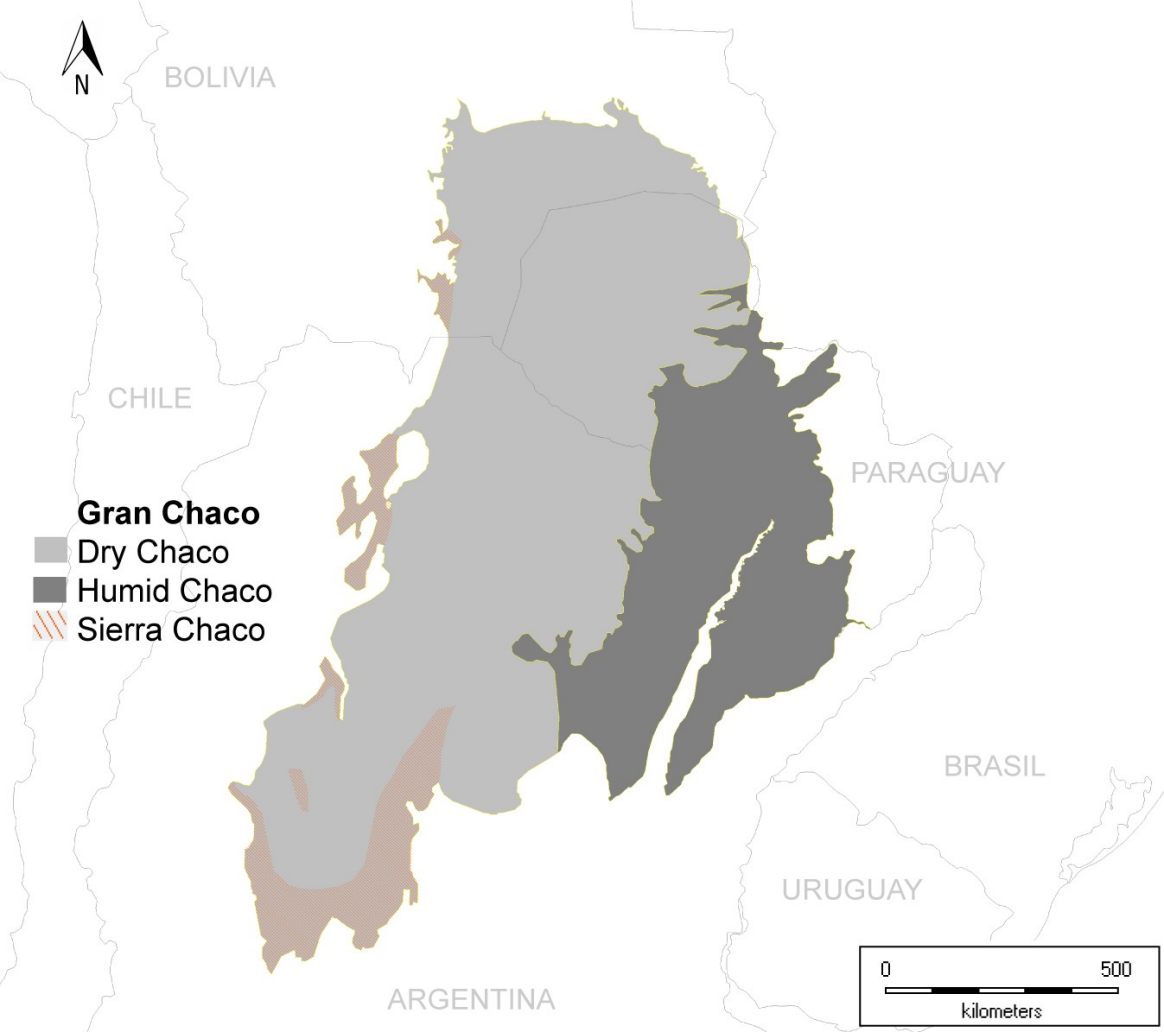
Delimitation of the Gran Chaco ecoregion and subregions.

**Fig 2:**
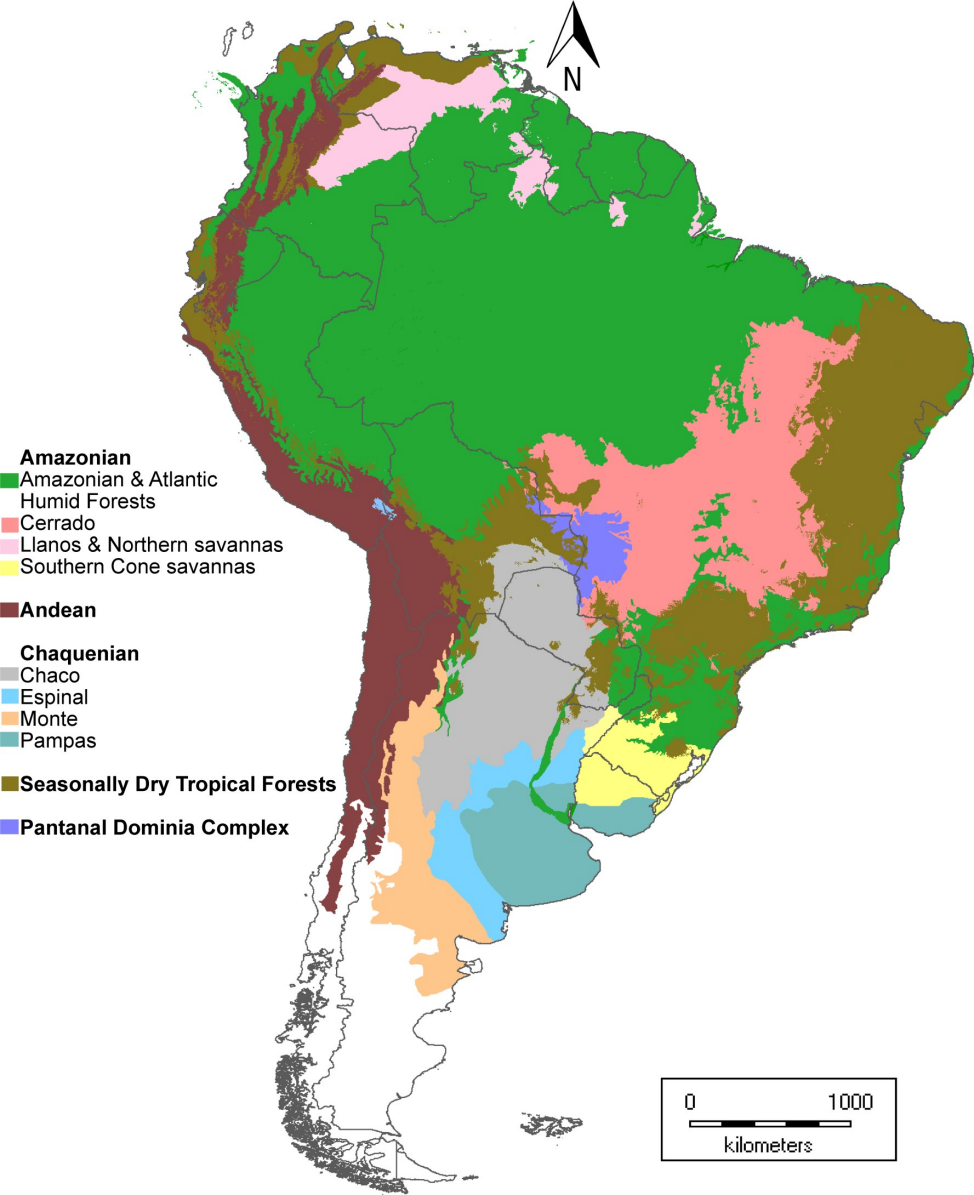
Distribution patterns of the hypothesized lineages (= Domains) of species from Chaco Ecoregion. In total we registered 98 genera, 362 species, and 404 specific and infraspecific Legume taxa occurring in the Gran Chaco ecoregion (Tables 3–5, S1 File). The number of endemisms or exclusive taxa for this region sum up to ca. 17% of the total of recorded species and 17% of total infraspecific taxa (Tables 3–6).

The term lineage for each species refers to the phytogeographical domain where the majority of known localities occur, but also takes into consideration the species distribution pattern, the main vegetation types where it has been registered, plus our own field observations and data from specimen’s labels. We defined the following domains to classify these lineages: 1) Chaco *s*.*s*. (*sensu* Prado 1993b); 2) Neotropical Seasonally Dry Tropical Forest (SDTF) (*sensu* [18]; 3) Amazonian (Southern Cone Savannas or Campos, Amazonian Rainforests, Cerrado); 4) Chaco-Andean (Table 2). The concept of each of these lineages was based in the following geospatial and phytosociological criteria (Table 2):

1. Chaco *s*.*s*.: The Chaco region is taken is a narrow biogeographical sense, consists of forests and woodlands on generally alkaline heavy clayish or silt-sandy soils, suffering seasonal yearly droughts and towards the east frequent floods, with extremely high temperatures in summer and frequent frosts in winter. The dominant species belong to the genera *Schinopsis*, *Prosopis*, *Acacia s*.*l*., *Capparis s*.*l*. and others. The Chaco *s*.*s*. was extensively discussed and redefined in [1, 2], and its flora was proved to be unique in its nature within the South American context [8, 10, 23]. In the Chaquenian lineage, we also included the biogeographical regions with floristic similarities: Monte and Espinal, formed by temperate scrublands, and Pampas, which consist in temperate grasslands mainly lacking native trees [11, 37]; see Table 2).
2. Seasonally Dry Tropical Forests (SDTF): these forests show a specific pattern of distribution in southern South America, which comprises allopatric populations from all or some of these South American regions, denominated “nuclei” [18, 23, 49]: a) Caatinga, in northeastern Brazil; b) the Misiones region of northeastern Argentina and neighboring Paraguay and Brazil; c) Piedmont, in the mountains foothills of northwestern Argentina and southern Bolivia. The three mentioned nuclei are connected by relicts throughout the Chiquitanía region, in Bolivia, and SDTF growing on calcareous, basic or alkaline soils in the Cerrado province [18, 19, 20, 23, 50]. Along western South America, the SDTF continues northward from Piedmont across dry inter–Andean valleys and some coastal dry forests, from Peru to Venezuela ([23]; Fig 1). A reduced transition between Chaquenian and SDTF lineages is present in the eastern extreme of Gran Chaco, as well as in some relicts of Bolivian Montane Dry Forests, but the latter was not included as Chaquenian lineage (Figs 1 and 2).
3. Amazonian: This lineage corresponds to the Amazonian Domain [9]. The genera with this lineage have diversified mainly in the huge Amazonas river basin and neighboring areas of Brazilian Planaltine and adjacent countries. The Amazonian Domain has several provinces. The taxa of this lineage were discriminated according to the province with major diversification.
  - Amazonian and Atlantic Humid Forests: Comprises the areas with humid tropical and subtropical forests: Amazonas basin, the Yungas rainforests in Andean foothills and the Atlantic coastal rainforests in Brazil. Some of its species can eventually disperse along the extended South American river system, such as the Paraguay river (connected to the Pantanal area) and the Paraná river-flooding valley [51], thus marginally reaching the Gran Chaco ecoregion. Extensive areas of the forests of the Paranaense province of [9, 11] include numerous species of clear Amazonian lineage. Recent works [52] suggest that Amazonian and Atlantic Humid Forest could be different provinces, but in the present work we considered them as a unit since the elements reaching the Gran Chaco are scarce.
  - Cerrado: This term refers to the extensive savannas of central Brazil, on strongly acid nutrient-poor red soils, though usually with high Aluminum levels toxic for Angiosperms in general, unless adapted to it, hence the very high level of endemicity (around 4,400 endemic species, according to [53].
  - Llanos and Northern Savannas: This province includes the tropical grasslands and savannas of Orinoco basin and adjacent areas in Colombia and Venezuela, as well as the Guiana Highlands.
  - Southern Cone Savannas or “Campos”: Corresponds to extensive grasslands in southern Brazil (Rio Grande do Sul), northeastern Argentina (eastern Corrientes and southeastern Misiones), northern Uruguay and some smaller areas in southern Paraguay. These grasslands are frequently dominated by the tall grass species *Andropogon lateralis* Nees. This formation has been described by [54] for Argentina and Uruguay, and by [55] for Brazil.
4. Andean. This Domain includes mountainous areas of Argentina, Bolivia, Chile and Peru, extending to the coastal deserts of the last two countries. Species indicated here as Chaco-Andean lineage have a disjunction in their distribution, appearing in Andes mountains, western coast of South America and the Gran Chaco or adjacent ecoregions.
5. Generalist. It refers to species with an ample distribution across several biomes and with not specific geographical pattern identified according to occurrence points.

**Table 2:** Lineages/Distribution patterns comparison with other biographical divisions and ecoregions for the Gran Chaco and adjacent areas.

Pattern/lineage	Cabrera & Willink (1980)	Olson et al. (2001)	DRYFLOR (2016) mapping
Chaco	Chaco Domain (in part)–Chaco, Espinal, Monte, Prepuna and Pampas (in part) provinces.	Humid Chaco and Dry Chaco, Espinal, Monte, Pampas, and Uruguayan Savannas (in part) ecoregions.	-
Seasonally Dry Tropical Forests (SDTF)	Chaco Domain (in part)–Caatinga Province.	Caatinga ecoregion	Caaatinga group/nucleous
	Amazonian Domain (in part)–Yungas, province (in part).	Central Andean Yungas (in part) and Peruvian Yungas (in part) ecoregion	Piedmont, Taparapoto–Quillabamba, Apurimac-Mantaro groups/nucleous.
	Amazonian Domain (in part)–Amazonian province (in part).	Dry Chiquitano Forest ecoregion	Piedmont (in part), Central Brazil groups/nucleous.
	Amazonian Domain (in part)–Paranaense province (in part)	Alto Paraná Atlantic Forests ecoregion (in part)	Misiones group/nucleous
	Pacific province (in part)–Desierto province (in part)	Tumbesian–Andean Valleys Dry Forests ecoregion	Central Andes Coast group/nucleous
	Yungas province (in part)–Pacific province (in part)	Eastern Cordillera Real Montane Forests ecoregion	Central Inter–Andean Valleys group/nucleous
	Pacific province (in part)–Yungas province (in part)	Northwestern Andean montane Forests ecoregion–Cordillera Real montane forests.	Northern inter–Andean valleys
	Sabana province (in part)–Guajira province–Venezuelan province–Amazonas province (in part).	La Costa xeric shrublands, Apure–Villavicencio Dry Forests, Guajira–Barranquilla xeric scrubs, Maracaibo Dry Forests (in part) ecoregions	Central America–Northern South America group/nucleous
Amazonian-Southern Cone Savannas or Campos	Amazonian Domain–Paranaense province (in part)	Southern Cone Mesopotamian Savannas, Uruguayan Savannas (in part) and Alto Paraná Atlantic Forests (in part) ecoregions.	-
Amazonian-Humid rainforests	Amazonian Domain–Amazonian Province	Moist forests of Madeira-Tapajós, Utauma-Trombetas, Japurá-Solimoes-Negro, Guianan piedmont, Southwestern Amazonian, Amazonian River and Flooded Forests, Caquetá, Maranhao-Babaçú, Negro-Branco, Solimoes-Japurá, Napo, Iquitos várzea, Purus-Madeira, várzea, Rio Negro campinarama.	-
		Alto Paraná Atlantic Forests (in part) and *Araucaria* moist forests	
Amazonian-Cerrado	Amazonian Domain–Cerrado province	Cerrado ecoregion	-
Amazonian-Llanos and Northern Savannas	Amazonian Domain–Guyana province–Sabana province	Llanos Savannas (in part); Guianan Savanna ecoregions	
Chaco/Andean	Desert, Chilean, Alto-Andean, Puna, Pacific and Páramo provinces.	Sechura Desert, Atacama Desert, Central Andean dry Puna, Central Andean Wet Puna, Southern Andean Steppe, Chilean Matorral ecoregions.	-

#### Subregions of Gran Chaco

We assumed a division of the Gran Chaco ecoregion in three main ecoregions or subregions: Dry Chaco, Humid Chaco, and Sierra Chaco. We followed the criterion of [37] to delimit Humid Chaco from Dry Chaco, whereas Sierra Chaco was defined by us on the basis of the phytogeographical maps of [11, 12, 13, 56]. Another potential subdivision of Dry Chaco, the so-called Arid Chaco [21], was not used here since it is not usually mapped amongst the ecoregions of the world. In the case of the Sierra Chaco, we followed the criterion of Prado [1] and [57], and only two of the three levels of vegetation (up to 1,750 m above sea level) were analyzed and considered. The remainder levels of vegetation comprise the flora of the highest altitudes of the Sierra Chaco, which differs entirely from the Gran Chaco ecoregion and has been recently raised to the province level under the Comechingones name [58]. These levels exhibit predominantly elements from Patagonian and Andean lineages, with forests where *Polylepis* Ruiz & Pav. is dominant, and grasslands comprising mostly cold-temperate grasses and some Asteraceae, among others [11]. Consequently, these areas were excluded from this study.

#### Endemicity

As regards the area of origin, all taxa were classified in endemic or non-endemic to Chaco. The criterion was strictly based in the exclusive or non-exclusive occurrence of the considered taxa within the boundaries of the ecoregion (Figs 1 and 2). In some particular cases, we considered some taxa as “typical”, when they showed most locations within the Gran Chaco and only minor locations outside (Tables 3–6).

**Table 3:** Checklist of species and infraspecific taxa of Cercidoideae and Detarioideae subfamilies, their distribution in the Gran Chaco, lineage and endemism.

Subfamily	Genus	Specific epithet	Variety	Subregion			Lineage	Chaco-endemic/typical
				Humid	Dry	Serrano		
Cercidoideae	*Bauhinia*	*aculeata*	* *		x		SDTF	no
Cercidoideae	*Bauhinia*	*argentinensis*	*megasiphon*		x	x	Chaco	yes
Cercidoideae	*Bauhinia*	*argentinensis*	*argentinensis*	x	x		Chaco	yes
Cercidoideae	*Bauhinia*	*bauhinioides*		x			Generalist	no
Cercidoideae	*Bauhinia*	*forficata*	*pruinosa*	x		x	SDTF	no
Cercidoideae	*Bauhinia*	*mollis*	*mollis*	x	x		Generalist	no
Cercidoideae	*Bauhinia*	*mollis*	*notophila*			x	SDTF	no
Cercidoideae	*Bauhinia*	*hagenbeckii*		x	x		SDTF/Chaco	yes
Cercidoideae	*Bauhinia*	*pentandra*			x		Generalist	no
Detarioideae	*Copaifera*	*langsdorfii*	*grandifolia*	x			Generalist	no
Detarioideae	*Copaifera*	*langsdorfii*	*laxa*	x			Generalist	no
Detarioideae	*Cynometra*	*bauhiniiifolia*	*bauhiniifolia*	x			Amazonian	no
Detarioideae	*Hymenaea*	*stygonocarpa*		x	x	SDTF	no	no

References: SDTF, Seasonally Dry Tropical Forests

**Table 4:** Checklist of species and infraspecific taxa of Caesalpinioideae subfamily, their distribution in Chaco, lineage and endemism.

Genus	Specific epithet	Subspecies	Variety	Form	Subregion			Lineage	Chaco-endemic/typical
					Humid	Dry/Arid	Sierra		
*Acacia*	*albicorticata*					x		SDTF	no
*Acacia*	*aroma*				x	x	x	Chaco/Andean	no
*Acacia*	*atramentaria*				x	x	x	Chaco	no
*Acacia*	*bonariensis*				x	x	x	Generalist	no
*Acacia*	*caven*		*caven*		x	x	x	Chaco/Andean	no
*Acacia*	*caven*		*dehiscens*			x		Chaco	no
*Acacia*	*caven*		*microcarpa*		x	x		Chaco	yes
*Acacia*	*caven*		*sphaerocarpa*		x			Chaco	no
*Acacia*	*caven*		*stenocarpa*		x	x		Chaco	no
*Acacia*	*curvifructa*				x	x		Chaco	yes
*Acacia*	*emilioana*					x		Chaco	yes
*Acacia*	*etilis*					x	x	SDTF	no
*Acacia*	*farnesiana*				x	x	x	Generalist	no
*Acacia*	*gilliesii*					x	x	Chaco	no
*Acacia*	*martii*				x			SDTF	no
*Acacia*	*monacantha*			*monacantha*	x			SDTF	no
*Acacia*	*monacantha*			*schulziana*	x			SDTF	yes
*Acacia*	*paniculata*				x	x		SDTF	no
*Acacia*	*parviceps*				x		x	SDTF	no
*Acacia*	*polyphylla*				x	x		SDTF	no
*Acacia*	*praecox*				x	x	x	Chaco	no
*Acacia*	*riparia*				x			SDTF	yes
*Acacia*	*tucumanensis*				x		x	SDTF	no
*Acacia*	*visco*					x	x	Chaco/Andean	no
*Albizia*	*inundata*				x	x		Generalist	no
*Albizia*	*niopoides*				x			SDTF	no
*Anadenanthera*	*colubrina*		*cebil*		x	x	x	SDTF	no
*Anadenanthera*	*colubrina*		*colubrina*		x	x		SDTF	no
*Anadenanthera*	*peregrina*				x			SDTF	no
*Arquita*	*mimosifolia*						x	Chaco	no
*Calliandra*	*brevicaulis*		*glabra*		x			Campos	no
*Calliandra*	*foliolosa*				x			Cerrado/Paranaense	no
*Calliandra*	*haematocephala*		*boliviana*			x		SDTF	no
*Calliandra*	*harrisi*					x		SDTF	no
*Cenostigma*	*pluviosum*				x	x	X	SDTF	no
*Cercidium*	*praecox*		*praecox*		x	x	x	Generalist	no
*Cercidium*	*praecox*		*australe*				x	Chaco/Andean	no
*Chamaecrista*	*arachyphylla*					x		Chaco	yes
*Chamaecrista*	*calycioides*				x	x		Generalist	no
*Chamaecrista*	*cordistipula*				x			SDTF	no
*Chamaecrista*	*desvauxii*		*piribebuiensis*		*x*			SDTF	no
*Chamaecrista*	*flexuosa*				x			Generalist	no
*Chamaecrista*	*nictitans*	*brachypoda*			x			SDTF	no
*Chamaecrista*	*nictitans*	*disadena*	*pilosa*		x			SDTF	no
*Chamaecrista*	*nictitans*		*patellaria*		x	x	x	SDTF	no
*Chamaecrista*	*rotundifolia*		*rotundifolia*		x	x		Generalist	no
*Chamaecrista*	*serpens*		*serpens*		x			Generalist	no
*Chamaecrista*	*venturiana*				x	x	x	SDTF	no
*Chloroleucon*	*chacöense*				x	x	x	Chaco	yes
*Chloroleucon*	*foliolosum*				x	x	x	SDTF	No
*Chloroleucon*	*mangense*					x		SDTF	No
*Chloroleucon*	*tenuiflorum*				x	x		SDTF	no
*Denisophytum*	*stuckerti*					x		Chaco	yes
*Desmanthus*	*acuminatus*				x	x	x	Generalist	no
*Desmanthus*	*paspalaceus*				x	x		Generalist	no
*Desmanthus*	*tatuhyensis*		*tatuhyensis*		x			Generalist	no
*Desmanthus*	*tatuhyensis*		*brevipes*		x	x	x	Chaco	no* = *not shown
*Desmanthus*	*virgatus*				x	x	x	Generalist	no
*Enterolobium*	*contortisiliqum*				x	x	x	SDTF	no
*Erythrostemon*	*argentinus*					x	x	Chaco	yes
*Erythrostemon*	*coluteifolius*					x	x	Chaco	yes
*Erythrostemon*	*gilliesii*						x	Chaco	no
*Gleditsia*	*amorphoides*		*amorphoides*		x			SDTF	no
*Gleditsia*	*amorphoides*		*anacantha*		x	x		SDTF	no
*Hoffmanseggia*	*glauca*						x	Generalist	no
*Inga*	*urugüensis*				x			Amazonian	no
*Libidibia*	*paraguariensis*				x	x		Chaco	yes
*Lophocarpinia*	*aculeatifolia*				x	x		Chaco	yes
*Microlobius*	*foetidus*	*paraguensis*			x			SDTF	no
*Mimosa*	*balansae*				x			Chaco	no
*Mimosa*	*bifurca*				x			SDTF	no
*Mimosa*	*bimucronata*				x			Amazonian	no
*Mimosa*	*candollei*				x	x		Generalist	no
*Mimosa*	*castanoclada*					x		Chaco	yes
*Mimosa*	*centurionis*				x			Chaco	yes
*Mimosa*	*chacöensis*					x		Chaco	yes
*Mimosa*	*cordobensis*						x	Chaco	yes
*Mimosa*	*craspedisetosa*					x		Chaco	yes
*Mimosa*	*debilis*		*debilis*		x	x		Generalist	no
*Mimosa*	*debilis*		*angusta*		x			Cerrado	no
*Mimosa*	*detinens*					x	x	Chaco	yes
*Mimosa*	*diplotricha*				x			Generalist	no
*Mimosa*	*distans*	*distans*			x			Cerrado	no
*Mimosa*	*diversipila*		*subglabriseta*		x			Chaco/SDTF	no
*Mimosa*	*diversipila*		*diversipila*		x			SDTF	no
*Mimosa*	*dolens*	*callosa*			x			Generalist	no
*Mimosa*	*dolens*	*rigida*	*foliolosa*		x			Generalist	no
*Mimosa*	*dolens*	*acerba*			x			Generalist	no
*Mimosa*	*ephedroides*					x		Chaco	no
*Mimosa*	*farinosa*					x		Chaco	yes
*Mimosa*	*gracilis*		*leiocarpa*		x			Cerrado	no
*Mimosa*	*guaranitica*				x			Cerrado	no
*Mimosa*	*hexandra*				x	x		Chaco/SDTF	no
*Mimosa*	*invisa*		*invisa*		x			Cerrado	no
*Mimosa*	*morongii*				x			Chaco	yes
*Mimosa*	*oligophylla*				x			Campos	no
*Mimosa*	*petraea*				x			SDTF	no
*Mimosa*	*pigra*		*dehiscens*		x			Generalist	no
*Mimosa*	*pigra*		*pigra*		x			Generalist	no
*Mimosa*	*polycarpa*		*spegazzini*		x			Generalist	no
*Mimosa*	*pseudopetiolaris*				x			SDTF	yes
*Mimosa*	*sensibilis*		*sensibilis*		x	x		SDTF	yes
*Mimosa*	*somnians*				x			Generalist	no
*Mimosa*	*strigillosa*				x	x	x	Chaco disyunta con EEUU	no
*Mimosa*	*subsericea*				x			Cerrado	no
*Mimosa*	*tobatiensis*				x	x		SDTF	yes
*Mimosa*	*troncosoae*					x		SDTF	yes
*Mimosa*	*tweedieana*				x			Amazonian	No
*Mimosa*	*xanthocentra*	*mansii*			x	x		Generalist	no
*Mimosa*	*xanthocentra*	*xanthocentra*			x	x	x	Generalist	no
*Mimozyganthus*	*carinatus*					x	x	Chaco	yes
*Neptunia*	*plena*				x	x		Generalist	no
*Neptunia*	*pubescens*				x	x		Generalist	no
*Parapiptadenia*	*excelsa*				x			SDTF	no
*Parapiptadenia*	*rigida*				x			SDTF	no
*Parkinsonia*	*aculeata*				x	x		Generalist	no
*Peltophorum*	*dubium*				x			SDTF	no
*Piptadeniopsis*	*lomentifera*				x	x		Chaco	yes
*Plathymenia*	*reticulata*				x			Amazonian (Cerrado, Paranaense	no
*Prosopidastrum*	*globosum*					x		Chaco	no
*Prosopis*	*abbreviata*					x	x	Chaco	no
*Prosopis*	*alba*		*alba*		x	x	x	Chaco	no
*Prosopis*	*alba*		*panta*		x	x		Chaco	No
*Prosopis*	*affinis*				x	x		Chaco	no
*Prosopis*	*campestris*						x	Chaco	yes
*Prosopis*	*chilensis*		*chilensis*			x	x	Chaco/Andean	no
*Prosopis*	*elata*					x	x	Chaco	yes
*Prosopis*	*fiebrigii*				x	x		Chaco	yes
*Prosopis*	*flexuosa*					x	x	Chaco	no
*Prosopis*	*hassleri*		*hassleri*		x	x		Chaco	yes
*Prosopis*	*hassleri*		*nigroides*		x			Chaco	yes
*Prosopis*	*kuntzei*				x	x	x	Chaco	yes
*Prosopis*	*nigra*		*longispina*		x	x		Chaco	yes
*Prosopis*	*nigra*		*ragonesei*		x			Chaco	yes
*Prosopis*	*nigra*		*nigra*		x	x	x	Chaco	no
*Prosopis*	*nuda*					x		Chaco	yes
*Prosopis*	*pugionata*					x	x	Chaco	no
*Prosopis*	*reptans*					x		Chaco/Andean	no
*Prosopis*	*rojasiana*					x		Chaco	yes
*Prosopis*	*rubriflora*				x			SDTF	yes
*Prosopis*	*ruscifolia*				x	x		Chaco	no
*Prosopis*	*sericantha*					x		Chaco	no
*Prosopis*	*strombulifera*					x		Chaco/Andean	no
*Prosopis*	*torquata*					x	x	Chaco	no
*Prosopis*	*vinalillo*				x	x	x	Chaco	yes
*Pterogyne*	*nitens*				x			SDTF	no
*Senna*	*aculeata*				x			Generalist	no
*Senna*	*alata*				x			Generalist	no
*Senna*	*aphylla*					x	x	Chaco	no
*Senna*	*bicapsularis*				x			Generalist	no
*Senna*	*cernua*					x		Generalist	no
*Senna*	*chacoënsis*					x		Chaco	yes
*Senna*	*chloroclada*				x	x	x	Chaco	yes
*Senna*	*corymbosa*				x		x	Generalist	no
*Senna*	*hirsuta*		*leptocarpa*		x			Generalist	no
*Senna*	*hirsuta*		*puberula*		x	x		Chaco	no
*Senna*	*morongii*				x	x		Chaco	no
*Senna*	*obtusifolia*				x	x		Generalist	no
*Senna*	*occidentalis*				x	x	x	Generalist	no
*Senna*	*pendula*		*glabrata*		x			Generalist	no
*Senna*	*pendula*		*paludicola*		x	x		Generalist	no
*Senna*	*pilifera*		*pilifera*		x			Generalist	no
*Senna*	*praeterita*					x	x	SDTF	no
*Senna*	*rugosa*				x	x		Cerrado	no
*Senna*	*scabriuscula*				x			SDTF	no
*Senna*	*spectabilis*				x	x	x	Generalist	no
*Senna*	*spiniflora*					x		Chaco	yes
*Senna*	*subulata*						x	Chaco	no
*Stenodrepanum*	*bergii*					x		Chaco	no
*Zapoteca*	*formosa*					x	x	Generalist	no
*Zygia*	*morongii*				x			SDTF	no
*Zygia*	*pithecolobioides*				x	x		SDTF	no

References: SDTF, Seasonally Dry Tropical Forests

**Table 5:** Checklist of species and infraspecific taxa of Papilionoideae subfamily, their distribution in Gran Chaco, lineage and endemism.

Genus	Specific epithet	Subspecies	Variety	Subregion			Lineage	Chaco-endemic/typical
				Humid	Dry/Arid	Sierra		
*Acosmium*	*cardenasii*				x		SDTF	no
*Adesmia*	*bicolor*					x	Generalist	no
*Adesmia*	*cordobensis*					x	Chaco	yes
*Adesmia*	*macrostachya*			x		x	Chaco	no
*Adesmia*	*muricata*	*dentata*		x	x	x	Chaco	no
*Adesmia*	*muricata*	*gilliesii*		x	x	x	Chaco	no
*Aeschynomene*	*americana*			x			Generalist	no
*Aeschynomene*	*denticulata*			x			Chaco/SDTF	no
*Aeschynomene*	*falcata*		*falcata*	x			SDTF	no
*Aeschynomene*	*histrix*		*incana*	x	x		Generalist	no
*Aeschynomene*	*mollicula*				x		SDTF	no
*Aeschynomene*	*montevidensis*			x			Chaco/SDTF	no
*Aeschynomene*	*paraguayensis*			x			SDTF	yes
*Aeschynomene*	*parviflora*			x			Chaco/SDTF	no
*Aeschynomene*	*rudis*			x			Generalist	no
*Aeschynomene*	*sensitiva*			x			Generalist	no
*Aeschynomene*	*viscidula*			x	x		Generalist	no
*Amburana*	*cearensis*			x	x		SDTF	no
*Ancistotropis*	*peduncularis*			x			Generalist	no
*Apurimacia*	*dolichocarpa*					x	Chaco	yes
*Arachis*	*batizocoi*				x	x	SDTF	yes
*Arachis*	*correntina*			x			SDTF	yes
*Arachis*	*duranensis*				x	x	SDTF	yes
*Arachis*	*glabrata*		*glabrata*	x			SDTF/Cerrado	no
*Arachis*	*glabrata*		*hagenbeckii*	x			SDTF/Cerrado	no
*Arachis*	*hassleri*			x			SDTF	yes
*Arachis*	*lignosa*			x			Chaco	yes
*Arachis*	*microsperma*			x			Chaco	yes
*Arachis*	*nitida*			x			SDTF	no
*Arachis*	*paraguariensis*			x			Chaco/SDTF	yes
*Astragalus*	*distinens*			x	x	x	Chaco	no
*Calopogonium*	*sericeum*			x			SDTF	No
*Camptosema*	*ellipticum*			x		x	Generalist	no
*Camptosema*	*paraguariense*		*paraguariense*	x			SDTF	yes
*Camptosema*	*paraguariense*		*parviflorum*	x			SDTF	yes
*Camptosema*	*praeandinum*					x	Chaco/SDTF	no
*Canavalia*	*bonariensis*			x			SDTF	no
*Canavalia*	*brasiliensis*			x			Generalist	no
*Canavalia*	ensiformis*			x			Generalist	no
*Canavalia*	*mattogrossensis*			x			Amazonian	no
*Centrosema*	*angustifolium*			x	x		Generalist	no
*Centrosema*	*kermesi*			x			Chaco	yes
*Centrosema*	*pascuorum*			x			Generalist	no
*Centrosema*	*pubescens*			x			Generalist	no
*Centrosema*	*sagittatum*			x	x		Generalist	no
*Centrosema*	*virginianum*			x		x	Generalist	no
*Chaetocalyx*	*brasiliensis*			x			Generalist	no
*Chaetocalyx*	*chacoensis*				x		Chaco	yes
*Chaetocalyx*	*latifolia*		*setulifera*	x			SDTF	yes
*Chaetocalyx*	*latifolia*		*latifolia*	x			SDTF	no
*Chaetocalyx*	*longiflora*			x			Generalist	no
*Clitoria*	*cordobensis*					x	Chaco	no
*Clitoria*	*epetiolaris*			x			Generalist	no
*Clitoria*	*falcata*			x			SDTF	no
*Cocliasanthus*	*caracalla*			x		x	Generalist	no
*Collaea*	*argentina*					x	Generalist	no
*Collaea*	*stenophylla*			x			Generalist	no
*Cologania*	*broussonetii*					x	Generalist	no
*Condylostylis*	*candida*			x			Generalist	no
*Coursetia*	*brachyrhachis*				x		Generalist	no
*Coursetia*	*hassleri*			x	x	x	SDTF	no
*Crotalaria*	*chaco-serranensis*					x	Chaco	yes
*Crotalaria*	*incana*			x	x	x	Generalist	no
*Crotalaria*	*micans*			x			Generalist	no
*Crotalaria*	*pilosa*			x			Generalist	no
*Crotalaria*	*spectabilis*			x			Generalist	no
*Crotalaria*	*stipularia*			x		x	Generalist	no
*Cyclolobium*	*brasiliense*			x			SDTF	no
*Dalbergia*	*frutescens*			x			Generalist	no
*Dalea*	*elegans*					x	Chaco	yes
*Desmodium*	*affine*			x			Generalist	no
*Desmodium*	*barbatum*			x			Generalist	no
*Desmodium*	*burkartii*			x			Chaco	yes
*Desmodium*	*cuneatum*			x			Generalist	no
*Desmodium*	*distortum*			x			Generalist	no
*Desmodium*	*glabrum*				x		Generalist	no
*Desmodium*	*hickenianum*			x			Chaco/SDTF	no
*Desmodium*	*incanum*			x		x	Generalist	no
*Desmodium*	*intermedium*			x			Chaco	yes
*Desmodium*	*neo-mexicanum*				x	x	Generalist	no
*Desmodium*	*pachyrrhizum*			x		x	Chaco/SDTF	no
*Desmodium*	*polygaloides*			x			Chaco/SDTF	no
*Desmodium*	*tortuosum*			x		x	Generalist	no
*Desmodium*	*uncinatum*			x		x	Generalist	no
*Desmodium*	*venosum*			x			Generalist	no
*Dioclea*	*burkartii*			x			Amazonian	no
*Dioclea*	*violacea*			x			Amazonian	no
*Discolobium*	*leptophyllum*			x			SDTF	no
*Discolobium*	*pulchellum*			x	x		SDTF	no
*Discolobium*	*pauciyugum*			x			Amazonian	no
*Discolobium*	*psolareaefolium*			x			Amazonian	no
*Dolichopsis*	*paraguariensis*			x	x		Chaco	no
*Eriosema*	*platycarpon*			x			Cerrado	no
*Eriosema*	*simplicifolium*			x			Generalist	no
*Eriosema*	*tacuaremboense*			x			Campos	no
*Erythrina*	*crista-galli*		*crista-galli*	x			Chaco	no
*Erythrina*	*dominguezii*			x			SDTF	no
*Erythrina*	*falcata*				x	x	SDTF	no
*Galactia*	*benthamiana*			x	x		Generalist	no
*Galactia*	*dubia*				x		Chaco/Andean	no
*Galactia*	*glaucescens*			x			Generalist	no
*Galactia*	*glaucophylla*				x	x	Chaco	yes
*Galactia*	*latisiliqua*		*chacoensis*			x	Chaco/SDTF	yes
*Galactia*	*latisiliqua*		*latisiliqua*	x	x	x	Chaco/SDTF	no
*Galactia*	*longifolia*			x			Chaco	no
*Galactia*	*marginalis*			x		x	Generalist	no
*Galactia*	*paraguariensis*			x			SDTF	no
*Galactia*	*striata*		*crassirachis*			x	Generalist	no
*Galactia*	*striata*		*striata*	x		x	Generalist	no
*Galactia*	*texana*		*degasperii*	x	x		Generalist	yes
*Galactia*	*texana*		*texana*		x	x	Generalist	no
*Geoffroea*	*decorticans*			x	x	x	Chaco/Andean	no
*Geoffroea*	*spinosa*			x	x		SDTF	yes
*Helicotropis*	*linearis*			x			Generalist	no
*Holocalyx*	*balansae*			x			SDTF	no
*Indigofera*	*asperifolia*			x	x		Generalist	no
*Indigofera*	*guaranitica*			x			Chaco	no
*Indigofera*	*hirsuta*			x			Generalist	no
*Indigofera*	*microcarpa*			x	x		Generalist	no
*Indigofera*	*parodiana*				x	x	Chaco	yes
*Indigofera*	*sabullicola*			x			Generalist	no
*Indigofera*	*suffruticosa*			x	x	x	Generalist	no
*Indigofera*	*spicata*			x			Generalist	no
*Lathyrus*	*macrostachys*			x			Generalist	no
*Lathyrus*	*nigrivalvis*			x			Chaco	no
*Lathyrus*	*pusillus*			x			Generalist	no
*Leptolobium*	*elegans*			x			Cerrado	no
*Leptospiron*	*adenanthus*			x			Generalist	no
*Lonchocarpus*	*nitidus*			x			SDTF/Amazonian	no
*Luetzelburgia*	*sotoi*					x	SDTF	no
*Lupinus*	*gibertianus*		*berroanus*	x		x	Campos	no
*Lupinus*	*gibertianus*		*gibertianus*	x		x	Campos	no
*Lupinus*	*gibertianus*		*reineckianus*	x		x	Campos	no
*Machaerium*	*aculeatum*			x			Generalist	no
*Machaerium*	*eriocarpum*			x	x		Cerrado	no
*Machaerium*	*paraguariense*			x			SDTF/Cerrado	no
*Machaerium*	*pilosum*				x		SDTF	no
*Machaerium*	*scleroxylon*			x			SDTF	no
*Machaerium*	*stipitatum*			x			Paranaense	no
*Macroptilium*	*atropurpureum*				x		Generalist	no
*Macroptilium*	*bracteatum*			x			Generalist	no
*Macroptilium*	*erythroloma*			x			Generalist	no
*Macroptilium*	*fraternum*					x	Chaco/Andean	no
*Macroptilium*	*geophyllum*				x		SDTF	yes
*Macroptilium*	*lathyroides*			x	x		Generalist	no
*Macroptilium*	*longepedunculatum*			x			Generalist	no
*Macroptilium*	*martii*			x			SDTF	no
*Macroptilium*	*panduratum*			x	x		Chaco/SDTF	no
*Macroptilium*	*prostratum*			x			Generalist	no
*Macroptilium*	*psammodes*			x			Campos	no
*Medicago*	lupulina*			x	x	x	Generalist	no
*Medicago*	polymorpha*					x	Generalist	no
*Medicago*	sativa*					x	Generalist	no
*Melilotus*	albus*			x	x		Generalist	no
*Melilotus*	indicus*			x	x		Generalist	no
*Muellera*	*fluvialis*			x			Chaco/SDTF	no
*Muellera*	*nudiflorens*			x			SDTF	no
*Muellera*	*sericea*			x			Amazonian	no
*Myrocarpus*	*frondosus*			x			SDTF	no
*Neonotonia*	wightii*			x			Generalist	no
*Nissolia*	*fruticosa*		*fruticosa*	x	x	x	Generalist	no
*Otholobium*	*higuerilla*					x	Generalist	no
*Phaseolus*	*vulgaris*	*aborigeneus*				x	Generalist	no
*Phaseolus*	*lunatus*		*sylvester*	x			Generalist	no
*Poiretia*	*tetraphylla*			x	x		Chaco/SDTF	no
*Poissonia*	*hypoleuca*					x	SDTF	yes
*Pterocarpus*	*santalinoides*			x			Amazonian/Caribe	no
*Rhynchosia*	*balansae*		*balansae*	x	x		Chaco/SDTF	no
*Rhynchosia*	*balansae*		*psilantha*	x			Chaco/SDTF	no
*Rhynchosia*	*burkartii*			x	x		Chaco/SDTF	no
*Rhynchosia*	*corylifolia*			x			SDTF	no
*Rhynchosia*	*diversifolia*		*diversifolia*	x		x	Generalist	no
*Rhynchosia*	*diversifolia*		*prostrata*	x			Generalist	no
*Rhynchosia*	*edulis*			x	x	x	SDTF	no
*Rhynchosia*	*minima*			x			Generalist	no
*Rhynchosia*	*naineckensis*			x		x	SDTF	no
*Rhynchosia*	*senna*		*senna*	x	x	x	Generalist	no
*Rhynchosia*	*senna*		*texana*		x		Generalist	no
*Sesbania*	*exasperata*			x	x		Generalist	no
*Sesbania*	*virgata*			x	x		Generalist	no
*Stylosanthes*	*guianensis*		*guianensis*	x		x	Generalist	no
*Stylosanthes*	*guianensis*		*subviscosa*	x			Generalist	no
*Stylosanthes*	*hamata*			x			Generalist	no
*Stylosanthes*	*leiocarpa*			x			SDTF/Amazonian	no
*Stylosanthes*	*macrosoma*			x	x	x	Chaco	no
*Stylosanthes*	*maracajuensis*			x			Chaco/Cerrado	no
*Stylosanthes*	*montevidensis*		*montevidensis*	x	x	x	Generalist	no
*Stylosanthes*	*montevidensis*		*intermedia*	x	x		Generalist	no
*Stylosanthes*	*recta*				x		Chaco	yes
*Stylosanthes*	*scabra*				x	x	Generalist	no
*Stylosanthes*	*viscosa*			x			Generalist	no
*Sweetia*	*fruticosa*			x			Amazonian	no
*Tephrosia*	*adunca*			x	x		Chaco/Campos	no
*Tephrosia*	*cinerea*				x		Generalist	no
*Tephrosia*	*hassleri*			x			Chaco	yes
*Trifolium*	pratense*			x			Generalist	no
*Trifolium*	*polymorphum*		*polymorphum*	x			Generalist	no
*Trifolium*	repens*					x	Generalist	no
*Vicia*	*epetiolaris*		*epetiolaris*	x			Chaco	no
*Vicia*	*epetiolaris*		*microcarpa*	x			Chaco	no
*Vicia*	*graminea*		*transiens*	x			Chaco	no
*Vicia*	*graminea*		*graminea*	x			Chaco	no
*Vicia*	*macrograminea*			x			Chaco/Campos	no
*Vicia*	*nana*			x			Chaco	no
*Vicia*	*setifolia*		*setifolia*	x		x	Generalist	no
*Vicia*	*pampicola*		*pampicola*	x			Generalist	no
*Vigna*	*luteola*			x	x	x	Generalist	no
*Vigna*	*longifolia*			x			Generalist	no
*Zornia*	*crinita*			x		x	Generalist	no
*Zornia*	*cryptantha*			x			Generalist	no
*Zornia*	*latifolia*			x		x	Generalist	no
*Zornia*	*multinervosa*			x			Chaco	no
*Zornia*	*pardina*			x	x		Generalist	no
*Zornia*	*reticulata*			x			Chaco/SDTF	no
*Zornia*	*trachycarpa*			x	x	x	Generalist	no

References: SDTF, Seasonally Dry Tropical Forests

*exotic but naturalized, weed or invasive.

**Table 6:** Summary of endemic/typical Leguminosae taxa from Chaco ecoregion.

Subfamily	Species endemic/typical			Specific and infraspecific taxa		
	Total		%	Total	Endemic/typical	%
Cercidoideae	7	2	28	9	3	33
Detarioideae	3	0	0	4	0	0
Caesalpinioideae	150	38	26	174	41	24
Papilionoideae	202	22	11	217	24	11
Leguminosae	362	62	17	404	68	17

#### Conservation status

Once we defined the distribution pattern of each taxon in the Gran Chaco, we performed an assessment about the conservation status in Chaco-endemic and Chaco-typical taxa. We built a database with localities from 1,000–1,100 specimens; all of them were georeferenced and their taxonomic determinations checked.

In order to make a more accurate assessment of conservation for typical and endemic taxa from the Gran Chaco, we made two categorizations. Firstly, we adopted the B criterion of the Red List of International Union for Conservation of Nature [59]. In each case, we used the GEOCAT program [60] to calculate the Extent of Ocurrence (EEO) and Area of Occupancy (AAO). AAO was based in a grid size of 4 km^2^ for woody species and 2 km^2^ for herbaceous species. In some cases, we adjusted the grid size based on our knowledge of population size. Based in both parameters, the program provided a possible status for each taxon that was confirmed by revising the presence of at least two of the following conditions: 1) severely fragmented or few locations; 2) continuing decline in: a) EEO; b) AAO; c) area, extent or quality of the habitat; d) number of locations or subpopulations; and e) number of mature individuals; and 3) extreme fluctuations in a), b), d), or e) from 2).

On the other hand, we elaborated on a categorization based on criteria of PLanEAr (Plantas Endémicas de Argentina) Program [61] with modificactions, since them allow us to generate hierarchies exclusively with herbarium data. We had not enough field and populations data for all taxa, because our work was based mostly in deposited collections; it prevented us to apply the IUCN criteria to define the conservation status.

Based on our available data, we grouped the Chaco-endemic and typical taxa in the following categories:

1. Taxa widely distributed outside Chaco s.s. (i.e., non-typical and non-endemic taxa).
2. Taxa occurring exclusively in Gran Chaco, with more than 15 known localities and at least in two Chaquenian subregions.
3. Taxa present only in one subregion of Gran Chaco, with more than 15 known localities and lineal distance between furthest localities more than 30 km.
4. Taxa with not common occurrence, or restricted to a narrow area within a subregion of Chaco or bordering areas between subregions: 11 to 15 known localities or the lineal distance between furthest localities not so far as 30 km.
5. Taxa with restricted distribution as 4) but up to 10 known localities or occurring in areas with short-term threats (habitat destruction, overexploitation, intensive local use, not protected areas).

This categorization reflects gradually minor to major need to increase information of the Chaquenian taxa, trying to establish real short-term threats in the distribution area, the rarity of taxa and the spatial distribution, assuming that a taxon with allopatric or distant populations will be better preserved than taxa with sympatric, parapatric or closepopulations.

We assessed all Chaco-exclusive taxa, but also included some fewtaxa havinga nucleus of distribution in Gran Chaco with scarce populations in adjacent ecoregions or exhibiting a marked disjunction in their distributions. These exceptions are pointed out in the respective table.

Categorization of taxa with restricted distribution or few localities (which corresponds to 4) or 5) categories) was decided based on the following parameters deforestation rate per administrative area of occurrence for the period 2001–2012 (inferred from maps of [26] assuming a decision level of 0.02; 2) visible environmental local threats; 3) occurrence of taxa in protected areas, and 4) local uses of resources from the taxa (the last three were documented from herbarium label’s annotations or bibliography). When the taxon was positively associated to at least two of the following characters, it was classified as 5): a) annual deforestation rate higher than 0.02; b) present of visible threats; c) not presence of species in protected areas; d) concrete and intensive local uses by humans.

## Results

We mapped the Gran Chaco ecoregion and subregions, integrating spatial information from the literature mentioned in Material and Methods (Fig 1). Additionally, we generated a map of biogeographic provincies and Dominia considered equivalent to the lineages from South America and including SDTF as a separate unit (Fig 2).

**Fig 3:**
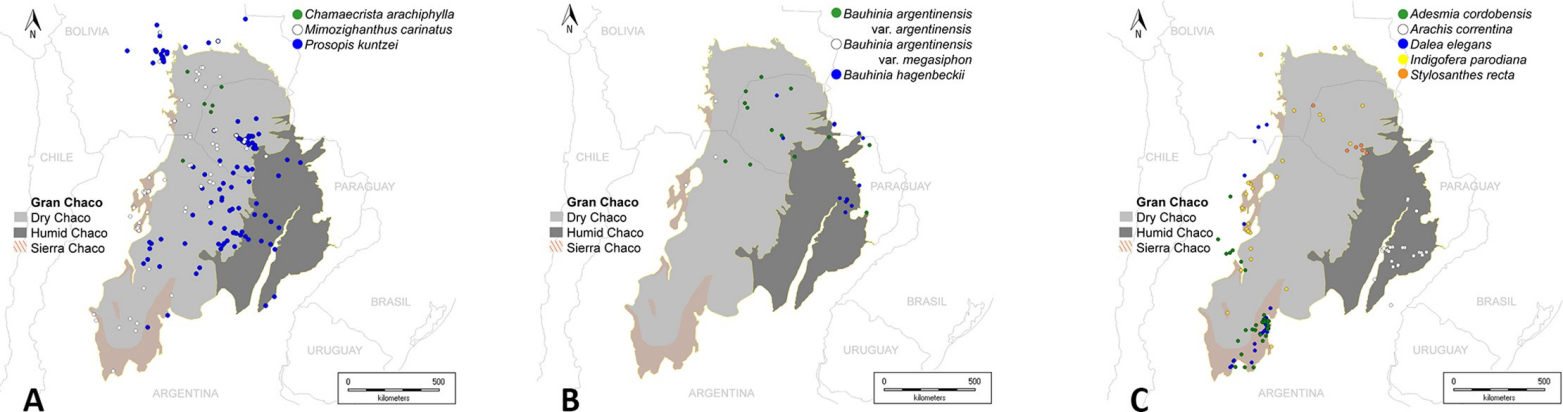
Distribution in South America of some endemic and typical species of Legumes from the Gran Chaco. A. Distribution in South America of some endemic and typical species of Caesalpinioideae subfamily from the Gran Chaco. B. Distribution in South America of some endemic and typical species of Cercidoideae subfamily from the Gran Chaco. C. Distribution in South America of some endemic and typical species of Papilionoideae subfamily from the Gran Chaco.

The most diversified Legume subfamily in Chaco is Papilionoideae, which comprises 202 species and 217 specific and infraspecific taxa occurring in this region. Caesalpinioideae comprises 150 species and 174 infraspecific taxa. In spite of its high diversity, Papilionoideae only comprises 22 species and 24 specific infraspecific exclusive taxa (11%), whereas Caesalpinioideae adds up 38 species and 41 specific and infraspecific taxa as endemic, which represents a higher percentage (ca. 24–26%) than the previous (Tables 3–6). Particularly, the Mimosoideae clade (*sensu* [30]), within Caesalpinioideae, comprised 35% of endemic and typical taxa (calculated from Table 4). We mapped some relevant endemic species of each subfamily (Fig 3).

**Fig 4:**
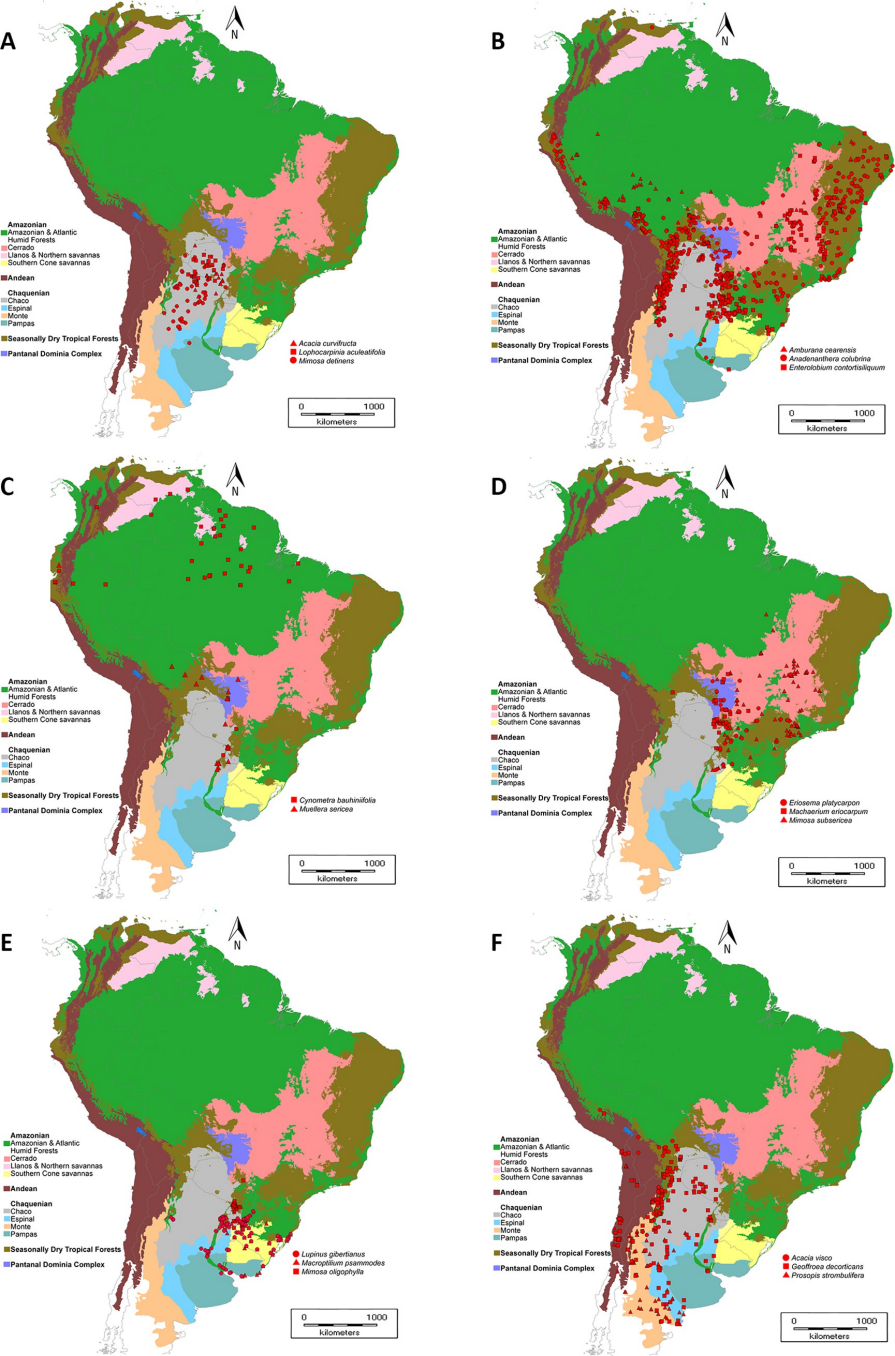
Distribution of typical species of the different lineages present in the Gran Chaco Ecoregion. A) Distribution of typical species of Chaquenian lineage. B) Distribution of typical species of Seasonally Dry Tropical Forests lineage. C) Distribution of typical species of Amazonian lineage–Amazonian and Atlantic rainforests. D) Distribution of typical species of Amazonian lineage–Cerrado. E) Distribution of typical species of Amazonian lineage–Campos. F) Distribution of typical species of Chaquenian/Andean lineage.

As regards the species lineages, the Chaco Legumes are predominantly generalist (139 species, 39%), though Chaco *s*.*s*. and SDTF lineages are also well represented and diverse (82 and 75 species, 23 and 21%, respectively). However, when each subfamily is analyzed separately, the percentages differ markedly since Caesalpinioideae predominantly comprises species with a Chaco *s*.*s*. lineage (51 species, or 34%), while Papilionoideae shows a tendency similar to the whole family, both with 98 species (49%) (Tables 3–5 and 7; S1 File). We mapped the distribution pattern of the most typical species of each lineage (Fig 4).

**Table 7:** Classification of Chaco species according to their lineage.

Subfamily	Total Species	Generalist		Chaco		SDTF		Chaco/ SDTF		Cerrado		Chaco/ Andean		Amazonian		Campos		Other	
		Species	%	Species	%	Species	%	Species	%	Species	%	Species	%	Species	%	Species	%	Species	%
Cercidoideae	7	3	38	1	12	2	28	1	12	-	-	-	-	-	-	-	-	-	-
Detarioideae	3	1	33	-	-	1	33	-	-	-	-	-	-	1	33	-	-	-	-
Caesalpinioideae	150	37	25	51	34	40	27	2	1	6	4	6	4	3	2	2	1	3	2
Papilionoideae	202	98	49	30	15	33	16	16	8	3	1	3	1	7	3	3	1	9	4
Leguminosae	362	139	39	82	23	76	21	19	5	9	2	9	2	11	3	5	1	12	3

The subregion with the highest number of species and infraspecific taxa is the Humid Chaco, comprising 161 species and 182 specific and infraspecific taxa, whilst the Dry Chaco and Sierra Chaco add up to 42 taxa (10%) and the latter, 25 (6%) taxa. Those taxa growing in both Humid and Dry Chaco subregions sum up to 42 species and 66specific and infraspecific taxa (16%), while those growing in all the three subregions include only 40 species as well as 42specific and infraspecific taxa; the rest of the taxa occur in some of two of three Chaco subregions. From all these subregions, the highest percentages of exclusive specific and infraspecific taxa correspond to the Dry Chaco and Sierra Chaco (23 and 12%, respectively, and ca.16% in species growing simultaneously in both subregions), whereas the Humid Chaco holds a lower percentage (ca.22%) (Tables 3–5, 8 and 9); the same tendency is found when the only species level is analyzed, which is not shown here.

**Table 8:** Classification of Chaco species and infraspecific taxa according to occurrence per subregions.

	Total taxa	Humid Chaco		Dry Chaco		Sierra Chaco		Humid/Dry Chaco		Dry/Serrano Chaco		Humid/Serrano Chaco		Three subregions	
		Taxa	%	Taxa	%	Taxa	%	Taxa	%	Taxa	%	Taxa	%	Taxa	%
Cercidoideae	9	1	33	2	22	1	11	3	33	1	11	1	11	-	-
Detarioideae	4	4	100	-	-	-	-	-	-	-	-	-	-	-	-
Caesalpinioideae	174	59	34	26	15	7	4	37	21	16	9	3	2	26	15
Papilionoideae	217	118	55	14	6	19	8	26	12	8	4	16	10	16	7
Leguminosae	404	182	45	42	10	27	7	66	16	25	6	20	5	42	11

**Table 9:** Endemic and typical specific and infraspecific taxa of Legumes from Gran Chaco per subregion (% over total taxa of each subregion).

Humid Chaco		Dry Chaco		Sierra Chaco		Humid Chaco and Dry Chaco		Sierra Chaco and Dry Chaco		Three subregions	
Endemic/typical	%	Endemic/typical	%	Endemic/typical	%	Endemic/typical	%	Endemic/typical	%	Endemic/typical	%
15	22	16	23	8	12	14	21	11	16	4	6

The most diversified genera are: *Mimosa* (35 species and 41 specific and infraspecific taxa), *Prosopis* (21 species and 26 species and varieties), *Senna* Mill. (21 species and 22 species and varieties), *Acacia s*.*l*. (19 species, and 24 species and varieties) and *Desmodium* Desv. (15 species) (Tables 3–5).

As regards the number of endemic and typical taxa, it is interesting to point out the existence of four Chaco-endemic monotypic or ditypic genera: *Mimozyganthus* Burkart *Piptadeniopsis* Burkart *Lophocarpinia* Burkart and *Apurimacia* Harms (Tables 3–5). The most relevant genera in terms of endemism are *Acacia s*.*l*., *Mimosa*, *Prosopis*, *Chamaecrista*, *Senna* and *Caesalpinia* L. group, because all of them include ca.48% of endemic and typical taxa (Table 4). Finally, Papilionoideae shows a low percentage of endemic and typical taxa (11% of its taxa) and its most diversified genera (*Desmodium* Desv., *Galactia* Browne, *Indigofera* L., and *Aeschynomene* L.) comprise only 0–35% of endemic taxa. In this subfamily, it is remarkable thatble that the genus *Arachis* L. shows 87% of Chaco-endemic species (Tables 4–6). The three genera of Detarioideae subfamily did not show endemic and typical taxa in Chaco (Table 3).

### Conservation status of Chaco-endemic and Chaco-typical taxa

According to the asseesment under the Red List criteria of IUCN, 13 species are Critically Endangered, 17 are Endangered, 9 are Vulnerable, 1 is Near Threatened, 3 have Deficient Data and 23 exhibit Low Concern (Table 10). Parameters related to IUCN Criteria of Categorization are shown as Supplementary material (S1 Table).

**Table 10:** Categorization of endemic taxa of Leguminosae in the Gran Chaco ecoregion.

Subfamily	Genus	Species	Variety	Form	Humid	Dry	Sierra	Minimum known localities	Distance between furthest localities	Conservation status	Maximum deforestation rate	Habitat threats	Protected areas	Human use	IUCN category	Observations
Caesalpinioideae	*Acacia*	*caven*	*microcarpa*		x	x		13	377	4	0.14	Deforestation	Partially	Unkwnon	LC	-
Caesalpinioideae	*Acacia*	*curvifructa*	* *		x	x		36	778	2	N/A	N/A	N/A	N/A	LC	-
Caesalpinioideae	*Acacia*	*emilioana*	* *			x		15	532	4	0.14	Deforestation	Partially	Unknown	VU	-
Caesalpinioideae	*Acacia*	*monacantha*	* *	*schulziana*	x			1	-	5	0.01	unknown	No	No	DD	
Caesalpinioideae	*Chamaecrista*	*arachyphylla*	* *			x		6	490	5	0.14	Yes	Partially	No	EN	-
Caesalpinioideae	*Chloroleucon*	*chacöense*	* *		x	x	x	12	805	4	0.09	Unknown	Partially	No	VU	One location in Pantanal
Caesalpinioideae	*Denisophytum*	*stuckerti*	* *			x		10	303	5	0.14	Deforestation	No	Unknown	VU	One population in the Parananse province
Caesalpinioideae	*Desmanthus*	*tatuhyensis*	*brevipes*		x	x	x	17	935	2	N/A	N/A	N/A	N/A	LC	Disjunction, with distribution in North America but in South America occurs strictly in Gran Chaco
Caesalpinioideae	*Erythrostemon*	*argentinus*	* *			x	x	7	625	5	0.07	Yes	No	No	EN	-
Caesalpinioideae	*Erythrostemon*	*coluteifolius*	* *			x	x	11	825	4	0.14	Possible deforestation	No	Unknown	VU	-
Caesalpinioideae	*Libidibia*	*paraguariensis*	* *		x	x		45	1380	2	N/A	N/A	N/A	N/A	LC	-
Caesalpinioideae	*Lophocarpinia*	* aculeatifolia*			x	x		30	480	2	N/A	N/A	N/A	N/A	VU	
Caesalpinioideae	*Mimosa*	*castanoclada*	* *			x		20	359	3	N/A	N/A	N/A	N/A	EN	-
Caesalpinioideae	*Mimosa*	*centurionis*	* *		x			3	124	5	0.04	Possible deforestation	No	No	CR	-
Caesalpinioideae	*Mimosa*	*chacöensis*	* *			x		12	763	4	0.14	Unknown	No	No	EN	-
Caesalpinioideae	*Mimosa*	*cordobensis*	* *				x	3	25	5	0.08	Tourism	No	No	CR	-
Caesalpinioideae	*Mimosa*	*craspedisetosa*				x		4	179	5	0.01	Possible deforestation	No	No	EN	-
Caesalpinioideae	*Mimosa*	*detinens*	* *		x	x	x	36	1107	2	N/A	N/A	N/A	N/A	LC	-
Caesalpinioideae	*Mimosa*	*morongii*	* *		x			4	22	5	0.02	unknown	No	No	CR	-
Caesalpinioideae	*Mimosa*	*pseudopetiolaris*	* *		x			6	64	4	0.01	unknown	Partially	No	EN	-
Caesalpinioideae	*Mimosa*	*sensibilis*	*sensibilis*		x	x		63	935	2	N/A	N/A	N/A	N/A	LC	Some locations in Pantanal and bordering Yungas
Caesalpinioideae	*Mimosa*	*tobatiensis*	* *		x	x		4	935	5	0.1	Deforestation	No	No	EN	-
Caesalpinioideae	*Mimosa*	*troncosoae*	* *			x		1	-	4	0.14	Deforestation	Yes	No	CR	-
Caesalpinioideae	*Mimozyganthus*	*carinatus*	* *			x	x	79	1857	2	N/A	N/A	N/A	N/A	LC	-
Caesalpinioideae	*Piptadeniopsis*	*lomentifera*	* *		x	x		20	602	2	N/A	N/A	N/A	N/A	EN	-
Caesalpinioideae	*Prosopis*	*campestris*	* *				x	7	265	5	0.15	unknown	No	No	EN	Two localitions in High Monte but is typically Chacoan species
Caesalpinioideae	*Prosopis*	*elata*	* *			x	x	18	765	2	N/A	N/A	N/A	N/A	LC	-
Caesalpinioideae	*Prosopis*	*fiebrigii*	* *		x	x		11	748	4	0.09	Possible deforestation	No	Unknown	LC	-
Caesalpinioideae	*Prosopis*	*hassleri*	*hassleri*		x	x		28	899	2	N/A	N/A	N/A	N/A	LC	-
Caesalpinioideae	*Prosopis*	*hassleri*	*nigroides*		x			2	-	5	0.01	unknown	No	No	DD	-
Caesalpinioideae	*Prosopis*	*kuntzei*	* *		x	x	x	44	838	2	N/A	N/A	N/A	N/A	LC	-
Caesalpinioideae	*Prosopis*	*nigra*	*ragonesei*		x			2	175	5	0.04	Deforestation	No	No	VU	-
Caesalpinioideae	*Prosopis*	*nigra*	*longispina*		x	x		2	19	5	0.05	Deforestation	No	Wood	VU	-
Caesalpinioideae	*Prosopis*	*nuda*	* *			x		11	583	4	N/A	N/A	N/A	N/A	VU	-
Caesalpinioideae	*Prosopis*	*pugionata*	* *			x	x	12	473	4	0.09	Deforestation	No	Possible wood	LC	One location in Southern Andean Steppe and other in High Monte
Caesalpinioideae	*Prosopis*	*rojasiana*	* *			x	x	8	390	5	0.14	Deforestation	No	No	EN	-
Caesalpinioideae	*Prosopis*	*rubriflora*	* *		x	x		5	173	5	0.06	Deforestation	No	No	EN	-
Caesalpinioideae	*Prosopis*	*vinalillo*	* *		x	x	x	18	1238	2	N/A	N/A	N/A	N/A	LC	-
Caesalpinioideae	*Senna*	*chacoënsis*	* *			x		14	805	4	0.07	Possible deforestation	No	Possible ornamental	LC	-
Caesalpinioideae	*Senna*	*chloroclada*	* *		x	x	x	57	816	2	N/A	N/A	N/A	N/A	LC	-
Caesalpinioideae	*Senna*	*spiniflora*	* *			x		30	554	3	N/A	N/A	N/A	N/A	LC	-
Cercidoideae	*Bauhinia*	*argentinensis*	*megasiphon*			x	x	3	478	5	0.09	Yes	No	No	EN	-
Cercidoideae	*Bauhinia*	*argentinensis*	*argentinensis*		x	x		21	714	3	N/A	N/A	N/A	N/A	LC	-
Cercidoideae	*Bauhinia*	*hagenbeckii*	* *		x	x		12	707	4	0.07	Possible deforestation	No	Unknown	NT	-
Papilionoideae	*Adesmia*	*cordobensis*	* *				x	33	928	3	N/A	N/A	N/A	N/A	LC	Two isolated populations in Prepuna/Monte
Papilionoideae	*Aeschynomene*	*paraguayensis*	* *		x			2	10	5	0.06	Yes	No	No	CR	-
Papilionoideae	*Apurimacia*	*dolichocarpa*	* *				x	4	23	4	0.01	unknown	No	No	CR	-
Papilionoideae	*Arachis*	*batizocoi*	* *			x	x	28	267	2	N/A	N/A	N/A	N/A	CR	-
Papilionoideae	*Arachis*	*correntina*	* *		x			30	585	3	N/A	N/A	N/A	N/A	LC	-
Papilionoideae	*Arachis*	*duranensis*	* *			x	x	50	712	2	N/A	N/A	N/A	N/A	EN	Ancestor of peanut
Papilionoideae	*Arachis*	*hassleri*	* *		x			3	203	5	0.14	Yes	No	No	CR	-
Papilionoideae	*Arachis*	*lignosa*	* *		x			3	205	5	0.1	Yes	No	No	CR	-
Papilionoideae	*Arachis*	*microsperma*	* *		x			1	-	5	0.04	Yes	No	No	CR	-
Papilionoideae	*Centrosema*	*kermesi*	* *		x			1	-	5	N/A	N/A	N/A	N/A	DD	Doubtful reports from Ecuador
Papilionoideae	*Chaetocalyx*	*chacoensis*	* *			x		11	250	4	0.14	Possible deforestation	Yes	Possible forage	EN	-
Papilionoideae	*Crotalaria*	*chaco-serranensis*	* *				x	31	1261	3	N/A	N/A	N/A	N/A	LC	-
Papilionoideae	*Dalea*	*elegans*	* *				x	32	1317	3	N/A	N/A	N/A	N/A	LC	Only two locations in Prepuna/Yungas, outside Gran Chaco
Papilionoideae	*Desmodium*	*burkartii*	* *		x			2	110	5	0.01	unknown	No	Possible forage	CRcr	-
Papilionoideae	*Desmodium*	*intermedium*	* *		x			2	187	5	0.015	unknown	No	Possible forageCR	-
Papilionoideae	*Galactia*	*glaucophylla*	* *			x	x	12	455	4	0.03	Deforestation	No	Possible forage	VU	-
Papilionoideae	*Galactia*	*latisiliqua*	*chacoensis*				x	15	1204	4	0.09	Deforestation	No	Possible forage	LC	-
Papilionoideae	*Galactia*	*texana*	*degasperii*		x	x		3	444	5	0.03	Yes	No	No	EN	-
Papilionoideae	*Indigofera*	*kurtzii*	* *				x	7	396	5	0.05	Tourism	No	Yes (tinctoria)EN	-
Papilionoideae	*Indigofera*	*parodiana*	* *			x	x	23	943	2	N/A	N/A	N/A	N/A	LC	One location in Bolivia
Papilionoideae	*Stylosanthes*	*recta*	* *			x		6	365	5	0.14	Deforestation	Partially	No	EN	-
Papilionoideae	*Tephrosia*	*hassleri*	* *		x			4	276	5	0.1	Deforestation	No	No	CR	-

According to the assessment that we made, from 66 endemic and typical taxa, 16 we classified in the category 2, 7 in category 3, 17 in the category 4 and 26 in the category 5 (Table 10, S1 File). The taxa with the most critical categories (4 and 5) occurred predominantly in Humid Chaco (14 of 29) and near half of them (20 of 43) belonged to the genera *Arachis*, *Mimosa*, and *Prosopis*.

## Discussion

The importance of the Legume family in the Chaco vegetation is conspicuous and evident. The number of species and infraspecific taxa of Legumes that we found in the present work is comparable to that of recent studies. In fact, [33] carried out a checklist of the woody legumes for the South American Corridor of Dry Vegetation, including the Chaco region, and they allegedly found 515 species, 324 of them exclusive of this vegetation type. However, the Chaco concept adopted by these authors does not match widely with the one proposed here; our conception was taken from well established literature along almost half a century (e.g. [2, 8, 12, 13, 20, 22]. In fact, the map of [33] shows a Chaco delimitation extending to areas devoid of typical Chaco vegetation (e.g. central north Bolivia) and at the same time omitting other typical Chaco vegetation zones (around half the Argentinean Chaco is left aside, no reasons provided).

Besides, [33] considered the gallery forests of the Paraná-Paraguay basins as part of the Chaco, which are either exclusively relicts of SDTF dominated by *Anadenanthera colubrina* [23] or part of the Paranaense province of [11]. In addition, [33] explicitly excluded several areas of central Argentina where Chaco-formations are characteristic and even dominant, such as northern Córdoba and San Luis provinces, a large part of Santiago del Estero province, and the entire region of western Argentina adjacent to pre-Andean foothills or Sierra Chaco (in the provinces of Jujuy, Salta, Catamarca, La Rioja, and San Juan). All these areas have been repeatedly treated as part of Chaco by all authors who have studied the phytogeography of the region [1, 2, 11, 12, 13]. These discrepancies in the delimitation of the Gran Chaco ecoregion have strongly influenced to emphasize the differences between both works on Legume diversity.

Additionally, [33] recorded several genera and numerous species occurring in some areas of the Chiquitanía region (northern Santa Cruz department, Bolivia). However, it has been demonstrated that the Chiquitanía region should be considered as a different ecoregion, the Chiquitano Dry Forest or the SDTF Chiquitano nucleus [20, 62]; here the indicator plant species of Chaco formations do not occur or, at least, they are not dominant. The Chiquitanía (encompassed between 15° and 19°S) is predominantly covered by SDTF with dominance of *Anadenanthera colubrina* and almost complete absence of species of *Prosopis* and *Schinopsis*. In addition, [33] assumed as Chaquenian several species growing in areas which we regard as typically of Amazonian lineage, such as Llanuras Benianas, Yungas rainforests, or the Madidi National Park (Prado 1993a,b; Olson et al. 2001). This is the case of several representatives of genera *Tachigali* Aubl., *Poeppigia* C. Presl., *Senna* Mill., *Bauhinia*, *Copaifera* L., *Martiodendron* Gleason, *Zygia* Benth. and Hook. F., *Zapoteca*, *Piptadenia* Benth., *Machaerium* Pers., *Dalbergia* L., *Dipteryx* Schreb., *Ormosia* Jacks., among other genera.

In the present study we have also segregated the Yungas Piedmont forests from the Chaco *s*.*s*., given that it has been clearly demonstrated that this formation is typically SDTF [10, 18, 23, 63]. The differences between the latter and Chaco *s*.*s*. in structure of vegetation and floristics have been repeatedly remarked by many authors [1, 2, 20, 50, 56]. Therefore, we could exclude from the concept of Chaco *s*.*s*. a number of species and genera that have been erroneously considered within the latter *s*.*s*. [33, 64].

Despite our more restrictive criterion to define the Chaco *s*.*s*., and the Gran Chaco ecoregion, which naturally means a lower number of species than in other works with an excessively wide Chaco definition, the Legume diversity that we found in this contribution is comparable to other several tropical formations of South America, though diversity data of some South American ecoregions is still lacking or incomplete. Nevertheless, there is reliable information available from the Brazilian Flora Checklist [34]; thus, this exhaustive Brazilian checklist allows us to compare the diversity of Chaco *s*.*s*. with other tropical, subtropical, and warm temperate plant formations.

Additionally, the Gran Chaco ecoregion is remarkable by its level of endemic and typical taxa, since we found that ca. 17% of all taxa is endemic. This percentage is similar or even higher than the percentages found by [33] for woody legumes of tropical ecoregions such as 'Thorny Shrublands' (which should be equivalent to Caatingas in [37, 38]) and Brazilian Savannas (equivalent to Cerrado ecoregion in [37, 38]).

It is possible also to compare the Legume diversity of Chaco *s*.*s*. with the diversity of other tropical or subtropical formations, taking into consideration all life forms. For example, Caatinga and Pampa (which is equivalent here to the Campos subregion of Amazonic Domain) have also ca. 25% of the species as endemic or exclusive, among Legumes [34]. It is notable that the Atlantic Forests biome includes a very high percentage of endemic species, ca. 41% [34]; however, there is a possibility that a part of these Atlantic Forests species considered endemic, could possibly occur also in certain parts of eastern Chaco (for example, in eastern Chaco and Formosa political provinces of Argentina), where there are similar formations relatively unexplored [2].

### Considerations per lineage

It is interesting to point out that most of the Gran Chaco Legume species are generalists or show proper Chaco *s*.*s*. or SDTF lineages. The intrusion of floristic elements from Amazonian lineages (from Cerrado, Amazonian forests or Campos provinces), seems to be not highly relevant both in absolute and relative terms and they are concentrated mainly in the Humid Chaco. The Amazonian elements appear to be descending by the major rivers and are restricted to the Paraná/Paraguay basins [65]. The relatively scarce presence of Amazonian elements suggests a clear ecological niche restriction from the true Chaco species, therefore ratifying the Chaco *s*.*s*. biogeographical limits [2]. Our results coincide with [52] clearly separating the Chaco forests from the rest of tropical and subtropical lowlands of South America.

Similarly, the presence of Andean elements reaching the Chaco via its western boundaries is restricted to a few particular taxa occurring in ecotones with arid or semiarid temperate ecoregions, such as the Monte or Prepuna [11]. This could be explained by the high complexity of the environments in the Andean foot-hills covering part of the western extreme of the Gran Chaco ecoregion. In this area, there are strong gradient variations in climatic and edaphic conditions in short distances and covering reduced spaces, thus forming very complex mosaics including strongly contrasting ecosystems, from high-altitude cold-steppes to almost-tropical rainforests [11, 56]. Therefore, the presence of mosaics of ecosystems with contrasting environmental conditions prevents a massive migration of species from the Andean domain to the Gran Chaco ecoregion.

In general terms, the presence of non-exclusive Chaco elements is registered predominantly in the eastern area of Chaco (Humid Chaco, according to [37, 38] and it can be explained by the presence of a mix of different plant formations in that region. In fact, eastern Paraguayan, Argentinean and Brazilian Humid Chaco are composed by mosaics of Chaco-forests, SDTF, and some grasslands floristically linked with southern Brazil. Actually, none of these areas have been deeply studied as yet ([1, 2, 19]; present authors personal observations).

In addition to this, vegetation patches comprising typical SDTF can be observed as relicts within the Dry Chaco, such as the vegetation of Cerro León and Cerro Cabrera [66], near to the Paraguay-Bolivia boundaries, which according to [2] should not be considered as Chaco proper. The Dry Chaco, in its northern sector, comprises also some transitional forests from Chaco to Chiquitanía, where there are mosaics of Chaco and SDTF vegetation [67]. Contrarily to the predominance of generalist species and intrusive lineages in the Humid Chaco, most Legume species from Dry/Arid Chaco and Sierra Chaco are native and exclusive to the ecoregion. This coincides with the predominance of typical Chaco-forests in these subregions, including *Schinopsis* and *Prosopis* species as dominant [1, 2, 11].

It is interesting to point out that the floristic stock of SDTF lineage occurring in either the eastern Humid or western Dry Chaco also includes many endemic and typical taxa, suggesting a remarkable level of diversification (20 out of 76 species, e.g. 26%, obtained from Tables 3–5), thus clearly distinguishing the discontinuous formation of SDTF in southern South America [18] from the Chaco *s*.*s*. [2]. In general terms, it has been demonstrated that each unit of SDTF in Latin America has a sizeable number of exclusive species of vascular plants [23], despite that these units share several common species. The same pattern has been observed specifically among legumes [18, 20, 50]. In this work, we could observe that approximately one third of the specific and infraspecific taxa with SDTF lineage occur in these formations but only within boundaries of the Gran Chaco ecoregion as a biome, but not within the Chaco *s*.*s*.

In this checklist, we included several exotic species of Legumes. Some of them, such as species of *Medicago*, *Melilotus*, *Trifolium*, are traditionally used as forage and they have been extensively naturalized in temperate Argentina [11]. We also included other species observed in the field as naturalized or at least registered as spontaneous, such as *Neonotonia wightii* (Arn.) J. A. Lackey and *Canavalia ensiformis* (L.) DC., since they could become either weed or invasive.

### Considerations per subfamily

Although the most extensive subfamily by number of species is Papilionoideae, the percentage of endemic and typical taxa is higher in Caesalpinioideae, and especially in the Mimosoideae clade, which is currently treated as a clade within Caesalpinioideae [30]. In the Mimosoideae clade, the presence of Chaco-exclusive entities is common in *Prosopis* and *Mimosa*, which are two of the three biggest genera occurring in the ecoregion. Indeed, *Prosopis* has its main diversification center in southern South America, especially in Argentina, Paraguay and Chile [68, 69], where the Chaco *s*.*s*. is included. This genus is particularly diverse in the Chaco by the number of species, presence of infraspecific taxa, frequent hybridization and introgression among its species, which generates new phenotypes, and characterized by the high degree of endemicity [68, 70, 71].

*Mimosa* is the genus with more representatives in the Gran Chaco ecoregion, but the presence of endemic taxa is less remarkable than in *Prosopis*. Even though, according to our present results, ca. 40% of its taxa are endemic or typical from the Gran Chaco, comprising at least six species of the Dry Chaco. It is interesting to point out that there are *Mimosa* species from ancestral and derived clades of the genus [72, 73]. In addition, the genus *Mimosa* comprises exclusive morphotypes forming taxonomical complexes, such as *M*. *debilis* Humb. &Bonpl. ex Willd. and *M*. *dolens* Vell., in the Gran Chaco [74, 75]. The presence of exclusive morphotypes suggests an incipient geographical speciation [75, 76].

The genus *Acacia* Mill. is the third in species number among Mimosoids from the Chaco *s*.*s*., but comprising relatively fewer Chaquenian endemic taxa (ca. 30%) than *Prosopis* and *Mimosa*. Most of their species show a SDTF lineage and they are found in other areas of South America, where these forests are present. The Chaco endemic species include *A*. *curvifructa* Burkart *A*. *emilioana* Fortunato & Ciald., as well as varieties of *A*. *caven* Molina and *A*. *monacantha* DC. [77, 78]. Meanwhile, *A*. *albicorticata* Burkart is endemic to the SDTF Piedmont nucleus and therefore not a typical Chaco species [77].

The remainder Mimosoideae genera are poorly represented in the ecoregion. In spite of their high diversity in other subtropical and tropical ecoregions, *Calliandra* Benth. and *Inga* Mill. have very few species growing in the Gran Chaco and none in the Chaco *s*.*s*. *Calliandra* includes four species [79] which are either generalist or SDTF lineages. In both genera, there are neither species with proper Chaco-lineage nor endemic species. *Inga* is represented by *I*. *urugüensis = I*. *vera* subsp. *affinis* (DC.) T. D. Penn., which is mainly restricted to the gallery forests of Humid Chaco [80, 81]; its presence is indicative of non-Chaco azonal vegetation [1]. It was stated that several species of *Inga* and *Calliandra* as occurring in the Gran Chaco ecoregion [33]. However, we analyzed the distribution pattern of each species cited for the region, and our conclusion is that most of them occur in Yungas rainforests or in the Amazonian region, coinciding with previous authors that analyzed the distribution of these genera [79, 81]. Therefore, both genera exhibit a clear Amazonian lineage, with their main centers of diversification in tropical humid forests from central and northern South America.

*Piptadeniopsis* and *Mimozyganthus* are both monotypic genera, endemic of the Chaco *s*.*s*. *Prosopidastrum* Burkart is a genus with Chaco-lineage but recently described new members occur predominantly in Monte and Espinal ecoregions, which are adjacent to Chaco; only *P*. *globosum* (Gillies ex Hook. &Arn.) Burkart has been mentioned for the Chaquenian area [82, 83]. *Neptunia* Lour. includes only two species with pantropical distribution growing in the Gran Chaco ecoregion [84]. The genera *Parapiptadenia* Brenan, *Chloroleucon* Britton & Rose ex Record and *Zygia* Benth. & Hook. f. comprise species mostly with SDTF lineage [85, 86]. The genus *Desmanthus* comprise in Chaco only three generalist species occurring in subtropical regions of South and North America [87] and *Zapoteca* is poorly represented, only two species grow in the region [88]. Both species of *Anadenanthera*, *A*. *colubrina* and *A*. *peregrina*, occur marginally in the Gran Chaco, and they are revelant because they are clear ecological indicators, the first one of SDTF, the second one of Cerrado and Amazonian savannas [23, 89]. Another monotypic genus present in Chaco is *Microlobius* [90].

The diversity of the remainder Caesalpinioideae in the Gran Chaco is determined mainly by two highly diversified genera: *Chamaecrista* Moench and *Senna*, but they have few Chaco-exclusive species. On the contrary, *Caesalpinia* L. group has fewer species but high percentage of endemic species. Remarkable Chaco *s*.*s*. endemic species of Caesalpinioideae include *Denisophytum stuckertii* (Hassl.) Gagnon & Lewis, *Chamaecrista arachyphylla* Barneby, and *Ch*. *cordistipula* (Mart.) Irwin & Barneby [91, 92], as well as some aphyllous species of *Senna* reaching Chaco from the Monte ecoregion, where this group exhibits a diversification center [90, 93]. It is noteworthy the existence of endemic *Lophocarpinia*, a monotypic genus exclusive to the Chaco *s*.*s*. region, which has been confirmedas a distinct genus in the last phylogenetic works [47, 94].

Most species of Caesalpinioideae are generalist, with several representatives of Chaco and SDTF lineages. A few genera of this subfamily, some of them monotypic, comprise indicator species of SDTF, such as *Pterogyne* Schrad. ex Nees, *Gleditsia*, and *Peltophorum* (Vogel) Benth. [18, 22, 23, 50].

The Papilionoideae appears to be the most diverse subfamily by its number of species and genera. Most species in this subfamily are shrubs to herbs, such as *Desmodium* (the most diverse genus in Chaco *s*.*s*.), *Galactia* P. Browne, *Macroptilium* (Benth.) Urb., *Indigofera* L., *Zornia* J. F. Gmel., and *Rhynchosia* Lour. [80, 95, 96, 97, 98, 99, 100]. Regarding this subfamily, it is very interesting to note that most of its species (half of them) have a generalist lineage, which would involve low morphological specialization in the Gran Chaco ecoregion, therefore coinciding with the low percentage of endemic taxa mentioned above.

Most of the Papilionoideae are exclusive of the Humid Chaco and their most diverse genera occur in temporarily inundated soils, in river coasts, and savannas or grasslands, such as *Aeschynomene*, *Centrosema*, *Chaetocalyx* DC., *Desmodium*, *Discolobium*, *Eriosema*, *Galactia*, *Stylosanthes* Sw., *Zornia* J.F. Gmel., and *Vicia* L. [96, 97, 101, 102, 103, 104, 105, 106, 107, 108, 109, 110, 111]. The genus *Crotalaria* comprises several generalist entities in Chaco, but it is remarkable the existence of a recently renamed endemic species in Sierra Chaco [112]. Some genera of Papilionoideae, either monotypic or poorly represented in the Gran Chaco, such as *Myrocarpus* Allemão, *Amburana* Schwacke & Taub. (a SDTF indicator species [18]), *Machaerium*, *Sesbania*, *Nissolia* Jacq., *Discolobium* Benth., *Lonchocarpus* Kunth, *Muellera* L.f., *Pterocarpus* Jacq., *Cyclolobium* Benth., *Dalbergia* L. f., *Erythrina* L., and *Sweetia* Spreng., are mainly confined to the Paraná and Paraguay basins, or peripheral areas of the Gran Chaco ecoregion [80, 108, 113, 114, 115, 116, 117, 118, 119, 120].

Among the genera with predominant herb and vines, *Dioclea* Kunth, *Canavalia* Adans., the complex of *Vigna* Savi *sensu lato*, and *Phaseolus* L. comprise a few exclusive species [48, 80, 121, 122, 123, 124]. The genus *Arachis* has 8 species in the Gran Chaco ecoregion with economical interest by their involvement in the origin of cultivated peanut and high diversification in Chaco and SDTF [125, 126].

In addition, within this subfamily, several genera predominantly diverse in temperate regions (e.g., Patagonian or High-Andean), such as *Lupinus* L., *Adesmia* DC., *Astragalus* L., *Dalea* L., *Lathyrus* L., *Medicago* L., *Melilotus* L., and *Trifolium* L., are poorly represented in the Gran Chaco, where generally fewer than 10 species occur in each of them [80, 127, 128, 129, 130, 131].

Additionally, several Papilionoid genera such as *Luetzelburgia* Harms, *Acosmium* Schott, and *Dalbergia*, are mostly tropical and they are poorly represented in Gran Chaco by less than ten species [132, 133, 134].

Other Legume subfamilies occurring in Chaco, according to the most recent systematic and phylogenetic treatment [30], are Cercidoideae and Detarioideae. Cercidoideae comprises only one genus in Chaco, *Bauhinia*, which is relevant by the presence of several endemic species, such as *B*. *argentinensis* Burkartand *B*. *hagenbeckii* Harms [135, 136, 137]. Detarioideae is also poorly represented in the region, comprising only three genera, *Copaifera*, *Cynometra*, and *Hymenaea*. The former includes only one species in the Gran Chaco, *C*. *langsdorffii* Desf., which is a widespread generalist [138], while the second one includes only *C*. *bauhiniifolia* Benth., with Amazonian lineage and two varieties recognized [139]. It is interesting to point out that both subfamilies are mainly distributed in equatorial and tropical regions [30], and therefore extending only marginally to the Gran Chaco and never into Chaco s.s. *Hymenaea* only presents one species in Gran Chaco, *H*. *stigonocarpa* Mart. ex Haine [140].

### Considerations per subregion

The Humid Chaco appears to be a biogeographically conflictive subregion, with some problematic entities difficult to classify. Strictly speaking, the Humid Chaco comprises the eastern extreme of the Gran Chaco ecoregion, in northeastern Argentina, central Paraguay and adjacent Brazil, covering approximately the area of the Paraná and Paraguay basins. Some authors [2, 19] have repeatedly statedthe presence of different and contrasting formations in this area in mosaic fashion, including SDTF and Chaco *s*.*s*. vegetations, which seem to be placed in Gran Chaco and establishing a transitional belt between the Chaco *s*.*s*., gallery forests and SDTF formations. Further studies are necessary to clarify the status of this area, which includes also some particular geological formations with interesting endemic species of Legumes, such as the Serranía de Tobatí and areas of the Concepción department, both in Paraguay [141].

Given the proximity to the Paraná-Paraguay river system, several elements with non-Chaco lineages are present in the Humid Chaco [65], including: 1) generalist species; 2) Amazonian, with elements from different provinces: a) Amazonian rainforests, whose genera and species form gallery forests in the Gran Chaco; b) Cerrado; and c) Campos species. All of them are intrusions from adjacent ecoregions and not true Chaco members.

Contrariwise, the Dry Chaco and Sierra Chaco comprise predominantly species with Chaco *s*.*s*. or sometimes SDTF lineages. The remarkablyfew species with non-Chaco lineages in both subregions should be explained partially by the absence of rivers or waterways communicating with other ecoregions, as well as the notable differences in climate in terms of minimum and maximum temperature extremes, and the total amount and strong seasonality of precipitations [2, 22].

The number of endemic and typical species is notoriously higher in Dry Chaco and Sierra Chaco than in the Humid Chaco. In fact, almost 39% of specific and infraspecific taxa in the two former subregions are endemisms, whilst only 16% of total species in the Humid Chaco are endemic. The higher levels of endemism in the western portions of the Gran Chacoecoregioncould be explained by the ecological isolation of these regions (given the scarce of large waterways connecting with adjacent ecoregions), as well as the conditions of extreme absolute minimum and maximum temperatures and dry climate (Prado 1993a). Thus, these environments would have adequate conditions to eco-geographical speciation, which is more evident in the Mimosoideae clade given its high species richness (particularly in woody species) [33, 80], the presence of numerous infraspecific taxa [35, 68, 71, 76], and possible hybridization [71], in the most diversified of its genera, *Acacia s*.*l*., *Mimosa*, and *Prosopis*.

The Sierra Chaco exhibits relatively few species of Legumes, and thiscould be explained by its relatively reduced area, as well as some lack of information as regards its plant diversity. The ecology of the Sierra Chaco requires intensive spatial and phytosociological studies, since its boundaries are not clearly defined in Bolivia and northern Argentina and for this reason the contribution of species to this checklist is still relatively poor. This subregion shows complex ecotones and mosaics of vegetation with other ecoregions, such as Yungas, Monte, Prepuna and Inter-Andean Dry Valleys [37, 56, 67].

### Additional remarks about conservation

Current and future scenarios in the Gran Chaco ecoregion are very complex to face biodiversity conservation, given the strong incidence of agriculture and cattle in the transformation of lands and climatic change. Inclusion of new agriculture technologies and increasing precipitation cycles has lead to drastic changes on land use in the Dry Chaco and Sierra Chaco, especially in the Argentine sector [28]. Coincidently, these subregions concentrate the highest percentage of endemic or exclusive taxa of Legumes family, and maybe also in the main vascular plant families, whose conservation status is essentially unknown. According to our categorization of the Gran Chaco endemic taxa, for most of them there are scarce information as regards its occurrence and distribution, or are restricted to certain areas with concrete short-term treats, being deforestation and expansion of cattle and soybean the most relevant.

Besides, it is remarkable the critical status of species which are relevant because of their economic potential, such as some endemisms of *Arachis* or *Prosopis*, which are important local resources as food, wood and forage [68, 142]. These species are also interesting to inbreeding programs of diverse crops, such as peanuts, *Arachis hypogaea* L. [118, 143]. Many *Arachis* species are in the category of Critically Endangered and they occur in very few localities with short-tem threats. Similar situation happens with some species and varieties of *Prosopis*, a genus with multiple applications among human communities of the Gran Chaco, because of their utility as food, forage, wood and timber; many are Endangered or Critically Endangered.

We here provide a first assessment about the conservation status for all Chaco-endemic Legume taxa. This information is a sound basis for future categorization of taxa under the criteria of IUCN. In fact, IUCN currently provides information of conservation status for only five Chaco-Endemic species [59]. According to IUCN, the only species which is Critically Endangered is *Mimosa morongii*, which we here categorized as 5, the most negative, given its restricted distribution and few known populations. We confirmed this status. Although *Libidibia paraguariensis* (D. Parodi) G. P. Lewis is Vulnerable according to IUCN, here we categorized them as 2 because of their wide distribution in the Gran Chaco. Additionally, *Galactia glaucophylla* Hams ex Kuntze and *Prosopis kuntzei* Harms are Low Concern under the IUCN criteria of IUCN, and our categorization of both of them coincide with 2, therefore expressing low concern given the wide distribution and high number of known populations.

Our categorization allows us to give an alternative assessment about conservation in those taxa with lacking data about distribution or population dynamics. In fact, it is possible to see that *Prosopis nuda*, *P*. *pugionata*, *Denysophyton stuckertii* or *Erythrostemon coluteifolius* have a category 4 or 5, i.e., they are truly endangered or threatened, but we are still unable to suggest a category according to IUCN. In addition, big sectors of the Paraguayan Dry Chaco and adjacent Humid Chaco have suffered considerable recent changes by cattle expansion [25], and situation is similar in Brazilian Chaco. In the Bolivian portion of the Gran Chaco, the knowledge about changes in land use is even more incipient, and reliable information about plant diversity and conservation is urgently needed since the area contains complex mosaics of several plant formations, such as Dry and Sierra Chaco, SDTF, and Cerrado [67].

## Conclusions

This contribution allows elucidating the relevance of Legumes in the biogeography of the Gran Chaco ecoregion. The percentage of endemism of the family in this subtropical ecosystem is very high, especially in the Caesalpinioidae subfamily, which could indeed have a relevant center of diversification in the region in its South American clades.

Our study also collected evidence for different degrees of diversification and intrusion of species from adjacent ecosystems. The arid portion of the Chaco seems to be the richest of Gran Chaco in terms of endemic taxa, and it is explained by its spatial isolation and more extreme climatic and edaphic conditions for the flora. This is especiallyobserved in big and/or Chaco-endemic genera, such as *Prosopis*, *Mimosa*, *Lophocarpinia*, *Piptadeniopsis* and *Mimozyganthus*.

An adequate regionalization of Chaco is still pending, especially in the context of rapid changes in landscape and biodiversity loss that this region is experiencing from several decades ago. The Legumes seem to be good indicators to evaluate regional divisions of Chaco associated to different ecosystems of phytogeographical formations. In fact, we found a high percentage of species with Chaquenian or SDTF lineages, which could explain the phytogeographical differences referring to the recent proposals in this South American ecoregion.

About one third of endemic and typical species of the Gran Chaco exhibits a status of conservation from endangered to critically endangered, or they have serious threatens given the drastical environmental changes in its ecosystems. For this reason, new intensive studies with more abundant data from the field are necesarry to monitore their populations.

## Supporting information

S1 File Lists of specimens from the Gran Chaco ecoregion. List of examined specimens.
(PDF)

S1 Table IUCN Categorization–criteria and parameters. IUCN Categorization—Criteria and Parameters.
(PDF)
