# Supplementary material for: Diversity and conservation of legumes in the Gran Chaco and biogeograpical inferences
Source: PLoS One. 2019 Aug 14;14(8):e0220151. doi: 10.1371/journal.pone.0220151 (PMC6693842; doi:10.1371/journal.pone.0220151)
Supplement: S1 File — List of examined specimens. (PDF) [file pone.0220151.s001.pdf]

S1 File. List of examined specimens

1. Representative specimens examined for all taxa of Legumes from Gran Chaco

Detarioideae

COPAIFERA

Copaifera langsdorfii

PARAGUAY. Cordillera: Tobatí, 28.III.1973, *P. Arenas 292* (CTES 118491).

We could not find material of *C. langsdorfii* var. *glabra,* which is suspected to grow in Paraguarí department (Paraguay), according to Fortunato et al. (2008).

CYNOMETRA

Cynometra bahuiniifolia* var. *bahuiniifolia

PARAGUAY. Concepción: Colonia Yby Yahú, empalme rutas 3 y 5, 14.XII.1983, *R. Vanni et l. 246* (CTES).

Cynometra bahuiniifolia* var. *meridiana

ARGENTINA. Formosa: Formosa, 11.XI.1909, *M. Lillo 313* (SI).

HYMENAEA

Hymenaea stygonocarpa

BOLIVIA. Santa Cruz: Cordillera, Parque Nacional Kaa-Iya del Gran Chaco, Paleodunas 25 Km. al NE de Palmar de las Islas, 12.II.1998, *A. F. Fuentes & G. Navarro 2279* (MO). PARAGUAY. Concepción: 500 m del cruce Barreto–Concepción, en dirección SE hacia Loreto, 23°01’S 57°02’W, 16.XII.1986, *L. Pérez et al. 1527* (BAB).

Cercidoideae

BAUHINIA

Bauhinia aculeata

BOLIVIA. Santa Cruz: Cordillera, Palmar de lãs Islas, Chaco, Ex-9, 19°15’39’’S 60°25’30’’W, 12.II.1998, *A. F. Fuentes & G. Navarro 2276* (MO).

Bahuinia argentinensis* var. *argentinensis

ARGENTINA. Formosa: Matacos, Ing. Guillermo N. Juárez, a 23 km al S por Ruta 39, 07.XI.1989, *R. H. Fortunato et al. 285* (BAB); Patiño, Estero Patiño, Parada 102, IX-1967, *Morello* s.n. (SI).

Bauhinia argentinensis* var. *megasiphon

BOLIVIA. Chuquisaca: Luis Calvo, El Salvador, Cimboc, fl., 27-I-1992, *Toledo* &*Joaquin 10436* (CTES).

Bahuinia bauhinioides

BRAZIL. Mato Grosso do Sul: Porto Murtinho,21°43'02,50"S,57°53'50,90"W, 19.XI.2008, *E.P.Seleme* 118 &*A.L.B.Sartori* (CGMS).

Bahuinia forficata* subsp. *pruinosa

ARGENTINA. Chaco: Colonia Benítez, 1931, *A. G. Schulz 95* (BAB).

NOTE: It is possible its occurence in Sierra Chaco, but we could not find specimens.

Bauhinia hagenbeckii

PARAGUAY. Cordillera: Eastern side of río Piribebuy basin, 17 km N of Arroyos y Esteros, 25°08’S 57°15’W, *E. M. Zardini & E. Velázquez 19200* (BAB).

Bahuinia mollis* subsp. *mollis

PARAGUAY. Alto Paraguay: Estancia Campo Grande, al norte por línea 28, 19°46’54’’S 58°46’54’’W, 10.V.2003, *L. Pérez de Molas & G. Navarro 8270* (BAB). ARGENTINA. Formosa: Pilcomayo, al este a 8 km P. Porteño, fr., 2-IV-1947, *Morel 2810* (LIL).

Bauhinia mollis* var. *notophila

BOLIVIA. Santa Cruz: Cordillera, Alto Parapetí, 04.I.1983, *R. de Michel 212* (BAB).

Bauhinia pentandra

BOLIVIA. Santa Cruz: Velasco, San Ignacio de Velasco, 30.IV.1986, *S. G. Beck & R. Seidel 12432* (BAB).

Caesalpinioideae

ACACIA

Acacia albicorticata

ARGENTINA. Salta: San Martín, 10 km S de Tartagal, 18.VIII.1991, *C. Saravia Toledo 10054* (CTES 178481).

Acacia aroma

ARGENTINA. Chaco: Charata, 30.IX.2004, *C. R. Salgado 371* (CTES55479); Güemes, interfluvio Del río Bermejo, 10 m del rio Bermejo, 15.IX.2006, *C. R. Salgado 371* (CTES 55479). Salta: Chicoana, ruta 68, próximo á divisa com Depto. La Viña, 1300 m, 25.I.2007, *J. Paula – Souza Et al. 7789* (CTES).

Acacia atramentaria

ARGENTINA. Corrientes: Esquina, Estancia La Blanca, 14.III.1975, *Schinini et al. 18206* (CTES106476). San Luis: Capital, Cruz de Piedra, en cercanías del dique homónimo, 03.IV.1987, *L. A. Del Vitto & E. Petenatti 1655* (CTES). Santiago del Estero: Bandera, VIII.1971, *Insfrán 862* (CTES).

Acacia bonariensis

PARAGUAY. Presidente Hayes: Around Pozo Colorado, 02.XI.2001, *E. M. Zardini & M. Vera 57234* (BAB). ARGENTINA. Formosa: Formosa, entre estancia Santa Catalina y Puerto Dalmacia, entre El rio Paraguay y la ruta 11 antigua, 13.XII.1967, *B. G. Piccinini & A. L. García 1142* (BAB). Tucumán: Capital, río Salí, 450 m, 10.I.1924, *S. Venturi 20a* (BAB 40873).

Acacia caven* var. *caven

BOLIVIA. Santa Cruz: Cordillera, Izozog, Cuarirenda, 07.VIII.1998, *A. Roca & E. Vaca 701* (CTES). ARGENTINA. Córdoba: Colón, Villa Allende, 23.II.1996, *A. Lamarque s.n.* (BAB 91947).

Acacia caven var. _dehiscens_

Argentina. Córdoba: Colón, 22.IX.1936, *E. G. Nicora 962* (SI).

Acacia caven var. _microcarpa_

ARGENTINA. Formosa: Formosa, en barrancas del río Paraguay, 06.I.1946, *A. Krapovickas 943* (SI); Bermejo, Fortín Soledad, *A. Krapovickas 1283* (SI).

Acacia caven var. _sphaeropcarpa_

ARGENTINA. Corrientes: Capital, 17.II.1989, *S.G. Tressens 3539* (CTES).

Acacia caven var. _stenocarpa_

ARGENTINA. Chaco: Primero de Mayo, 01.X.1944, *A. T. Hunkiker 5749* (SI). Santiago del Estero: Ojo de Agua, Sierra de Sumampa, Dique de Báez, 22.X.1979, *B. G. Piccinini & J. Hilfer 4060* (BAB).

Acacia curvifructa

ARGENTINA. Chaco: Almirante Brown, M. Jacuboski, 18,27 km ESE de las Cuatro Bocas, 06.XII.1977, *Bordón 526* (CTTES405401). Formosa: Pilcomayo, 12 km al SO de Filipina (Est. Salaberry), 21.XI.1949, *I. Morel 8930* (CTES 68176).

Acacia emilioana

BOLIVIA. Santa Cruz: Cordillera, Izozog, Comuinidad La Brecha, 12.VII.1998, *A. Fuentes 2505* (CTES).

Acacia etilis

BOLIVIA. Tarija: Villamontes, Quebrada de Tampinta, 28.V.1971, *A. Krapovickas et al. 19404* (CTES). ARGENTINA. Jujuy: Santa Bárbara, La Quinta, 24.X.1970, *Legname & Cuezzo 7780C* (CTES).

Acacia farnesiana

BRAZIL. Mato Grosso do Sul: Porto Murtinho, 21°43'02,50"S,57°53'51,30"W, 16.VII.2009, *E.P.*Seleme *et al.*367 (CGMS). PARAGUAY. Presidente Hayes: Estancia Zalazar, 28.VII.1997, *E. M. Zardini & L. Guerrero 46998* (BAB). ARGENTINA. Córdoba: Río Ceballos, VI.1939, *R. Lahitte* (BAB 60295).

Acacia furcatispinna

ARGENTINA. Salta: Rivadavia, Juan Solá (Morillo), 4 km S del pueblo, 28.VIII.1983, *P. Arenas 2556* (CTES143247).

Acacia gilliesii

PARAGUAY. Boquerón, A 3 km del cruce de picada Lobrego y entrada a Don Silvio, 19.V.1994, *R. Degen 3164* (FCQ, MO). ARGENTINA. Salta: La Viña, 28.II.1985, *R. Kiesling 5843* (SI).

Acacia martii

BRAZIL. Mato Grosso do Sul: Porto Murtinho, 21°42'03,50"S,57°41'17,10"W, 28.VIII.2007, *F. M. Alves et al. 464* (CGMS).

Acacia monacantha* f. *monacantha

ARGENTINA. Chaco: Bermejo, Isla del Cerrito , 10.III.1983, *J. J. Neiff 1563* (CTES).

Acacia monacantha* f. *schulziana

ARGENTINA. Chaco: Primero de Mayo, no date, *A. G. Schulz 2012*(SI).

Acacia paniculata

BOLIVIA. Santa Cruz: Cordillera, Puesto 27 de Noviembre, 16.VII.1998, *A. F. Fuentes & G. Navarro 2569a* (MO).

Acacia parviceps

ARGENTINA. Salta: Capital, Cerro San Bernardo, 1990, *Hoc & Lamarque s.n.* (BAB 91928).

Acacia polyphylla

BRAZIL. Mato Grosso do Sul: Porto Murtinho, 21°42'03,50"S,57°41'17,10"W, 09.V.2007, *F. M. Alves et al. 406* (CGMS).

Acacia praecox

PARAGUAY. Boquerón: 34 kim al S del cruce a Teniente Montanía desde Filadelfia, 10.III.2005, *R. H. Fortunato et al. 8644* (BAB). ARGENTINA. Chaco: General Güemes, ruta 5, 6 km SO del A.C.A. de J. J. Castelli, 16.XII.1983, *A. M. Molina 2282* (BAB). Tucumán: Trancas, Vipos, 07.X.1923, *S. Venturi 2477* (BAB).

Acacia riparia

PARAGUAY. Paraguarí: Cerro Palacios, 5 km N of Paraguarí, 26.I.1989, *E. M. Zardini & A. Aguayo 9837* (MO).

Acacia tucumanensis

BOLIVIA. Tarija: Gran Chaco, 30 km N de Yacuiba, Campo de la Tapia, cerca de la Caiza, 2 km E de la ruta a Villa Montes, 07.IV.1977, *A. Krapovickas & A. Schinini 30965* (CTES). PARAGUAY. Cordillera: Yhacá, 09.VII.1993, *E. M. Zardini & T. Tillería 36611* (MO).

Acacia visco

ARGENTINA. Catamarca: Camino de La Quebradita al Rodeo, 1000 m, 15.XI.1972, *Menéndez Sevillano et al. 9337C* (CTES). Salta: Metán, Metán, 19.X.1988, *Del Castillo 1079* (CTES).

ALBIZIA

Albizia inundata

BRAZIL. Mato Grosso do Sul: Porto Murtinho, 21°43'02,50"S, 57°53'51,30"W, 16.XII. 2008, *E.P.Seleme & A.L.B. Sartori190* (CGMS). ARGENTINA. Salta. Rivadavia: Río Teuco, al S de Las Blancas, 16-IX-1980, *M. N. Correa et al. 7563* (BAB).

Albizia niopoides

PARAGUAY. Paraguarí: Palacios Mountain, 25°25'S 57°10'W, 24.VI.1989, *E. M. Zardini & Velázquez 13189* (BAB).

ANADENANTHERA

Anadenanthera colubrina* var. *cebil

BRAZIL. Mato Grosso do Sul: Seleme, E.P. & Sartori, A.L.B. 213 (CGMS). ARGENTINA. Catamarca: Capital, Las Rejas, 07.IV.1974, *E. Ulibarri 879* (CORD, CTES 24606). Santiago Del Estero: Guasayán, Sierra de Guasayán, 26.XI.1974, *B. G. Piccinini & C. A. Petetin 3222* (BAB).

Anadenanthera colubrina* var. *colubrina

ARGENTINA. Corrientes: Itatí, Estancia Tuyutí, 19.VIII.1956, *T. M. Pedersen 3962* (CTES 362892). Santiago del Estero: Capital, ruta hacia Cristo de San Javier, 14.IX.2005, *Keller 3150* (CTES 408447).

Anadenanthera peregrina

PARAGUAY. Paraguarí: Cerro Mbatoví, 02.VII.1988, *E. M. Zardini 5459* (MO).

ANCISTOTROPIS

Ancistotropis peduncularis

E. P. Seleme & A. L. B. Sartori 169 (CGMS). Argentina: Formosa, 28.X.1900, *Kermes 442bis* (BAB 197).

CAESALPINIA GROUP

Arquita mimosifolia

Argentina. Córdoba: Quilpo (Canteras), 17.XII.2009, *J. J. Cantero 5932* (BAB).

Cenostigma pluviosum

BOLIVIA. Tarija: Caigua, 15 km N de Villamontes, 03.VI.1971, *A. Krapovickas et al. 19453* (BAB).

Denisophytum stuckertii

ARGENTINA. Santiago del Estero: Copo, 15 km NNW de Monte Quemado, 08.XII.1979, *A. Schinini 19497* (BAB).

Erythrostemon argentinus

BOLIVIA. Tarija: Villamontes, 28.V.1971, *A. Krapovickas et al. 19353* (BAB). Santa Cruz: Cordillera, Estancia Rancho Chico (puesto nuevo) y alrededores, 22.V.1998, *A. F. Fuentes 2390* (USZ).

Erythrostemon coluteifolius

ARGENTINA. Santiago del Estero: Río Hondo, 22.V.1949, *A. Soriano & Barrett 3572* (BAB). Tucumán: Trancas, entre Tapia y Vipos, 05.XII.1989, *L. Galetto 132* (CORD).

Erythrostemon gilliesii

ARGENTINA. Córdoba: San Albe3rto, Mina Clavero, a 2 km Del pueblo por El camino de lós Artesanos hacia Río Hondo, 01.III.1996, *A. Lamarque s.n.* (BAB 91953). Santiago del Estero: Ojo de Agua, ruta 9, 20 km N de Ojo de Agua, 19.X.1988, *R. L. Pérez Moreau & C. A. Petetin 4151* (BAB).

Libidibia paraguariensis

ARGENTINA. Formosa: Pilcomayo, Parque Nacional Pilcomayo, La Alegría, 11.XI.1991, *R. H. Fortunato et al. 2237* (BAB). Santiago del Estero: Figueroa, ruta provincial 5, puesto El Saladillo, salina, 04.III.1986, *A. M. Molina & J. Hilfer 2759* (BAB).

CALLIANDRA

Calliandra brevicaulis* var. *glabra

PARAGUAY. Paraguarí: Entre Florida y Caapucú, 04.II.1966, *A. Krapovickas et al. 12405* (CTES).

Calliandra foliolosa

PARAGUAY. Paraguarí, 35 km N of Caapucú, arroyo Apichapa, 29.IV.1994, *W. J. Hahn 2386*

Calliandra haematocephala

PARAGUAY. Boquerón: Colonia Fernheim, 14.IX.1990, *R. O. Vanni Et al. 2116* (MO).

Calliandra harrisii

PARAGUAY. Alto Paraguay: Cerro León, oeste del cerro, 15-XI-1992, *L. Ramella et al. 3016* (BAB).

(MO 3614031).

CERCIDIUM

Cerdicium praecox* var. *australe

ARGENTINA. Santiago del Estero: Belgrano, Bandera, camino a Cuatro Bocas, km10, 18.X.1972, *M. Elisetch & Cano 2* (BAB). Tucumán: Tapia, 31 km N de Tucumán, 06.XI.1948. *Dawson & Calastremé 1808* (BAB 71312).

Cercidium praecox* var. *praecox

BRAZIL. Mato Grosso do Sul: Porto Murtinho,21°41'03,50"S, 57°41'17,10"W, 08.V.2007, *F.M.Alveset al.* 346 (CGMS). BOLIVIA. Santa Cruz: Yanaigua, 30 km W of Paraguay border, 29.X.1991, *A. H. Gentry 75371* (MO). ARGENTINA. Formosa: Bermejo, Laguna Yema, alrededores del pueblo, 29.IX.1998, *R. Vanni 4267* (BAB). Tucumán: Trancas, Tapia, 14.XI.1922, *S. Venturi 1037* (BAB 40548).

CHAMAECRISTA

Chamaecrista arachyphylla

PARAGUAY. Boquerón: 1 km O de General Eugenio Garay, 08.V.1988, *A. Charpin & L. Ramella 21426* (BAB).

Chamaecrista calycioides

BOLIVIA. Santa Cruz: Cordillera, Territorio Comunal pampas de Isoporenda, 21.VII.1998, *A. F. Fuentes & G. Navarro 2639* (MO). ARGENTINA. Corrientes: Lavalle, 27.II.1961, *T. M. Pedersen 5824* (SI).

Chamaecrista cordistipula

PARAGUAY. Concepción: Loreto – Paso Horqueta, 6 km N of Loreto, 18.XI.1993, *E. M. Zardini et al. 37402* (BAB).

Chamaecrista desvauxii* var. *pirebebuiensis

PARAGUAY. Cordillera: Tobaty, september, *Hassler 6118* (G).

Chamaecrista flexuosa

ARGENTINA. Corrientes: Bella Vista, XII.1904, *N. Rojas Acosta s.n.* (BAB 13467).

Chamaecrista nictitans* subsp. *brachypoda

ARGENTINA. Chaco: General Güemes, Zaparinqui, 13 km N de J. J. Castelli, 20.I.1980, *B. G. Piccinini & J. Hilfer 4228* (BAB).

Chamaecristanictitans* subsp. *disadena

BRAZIL. Mato Grosso do Sul: Porto Murtinho,21°41'03,50"S, 57°41'17,10"W, 16.II.2007, F.M. Alves *et al.*11 (CGMS)

Chamaecrista nictitans* var. *patellaria

Paraguay. Alto Paraguay: Ea. Campo Grande al N por Línea 28, 11.V.2003, *L. Pérez de Molas & G. Navarro 8307* (BAB). ARGENTINA. Corrientes: Concepción, 10 km E de Tata Cuá, 15.III.2001, *DeMatteis et al. 949* (BAB). Formosa: Bermejo, alrededores de Bosch por ruta 81, 10.XI.1983, *R. H. Fortunato Et al. 413* (BAB). Salta: Rosario de Lerma, Quebrada Del Toro, 7 km al N de Campo Grande ingresando a La Quebrada Del Toro, 1661 m, 06.III.2001, *R. H. Fortunato et al. 6931* (BAB).

Chamaecrista rotundifolia

ARGENTINA. Chaco: General Güemes, alrededores de Wichi, camino a Sauzal, 29.II.2000, *R. H. Fortunato Et al. 6491* (BAB). Formosa: Estancia Bouvier, alrededores del puesto Luna, 21.XI.1991, *R. H. Fortunato et al. 2528* (BAB).

Chamaecrista serpens* var. *serpens

BRAZIL. Mato Grosso do Sul: Porto Murtinho, 21°43'02,50"S, 57°53'50,90"W, 20.XI.2008, *A.K.D. Salomão* 337 &*F.M. Alves* 337. ARGENTINA. Chaco: 25 km S de Campo Grande, en dirección a Sauzalito, *R. H. Fortunato et al. 6550* (BAB).

Chamaecrista venturiana

ARGENTINA. Formosa: Pirané, Ea. Irigoyen cerca de El Colorado, III.2000, *A. Perticari s.n.* (BAB 92108).

CHLOROLEUCON

Chloroleucon chacoënse

BRAZIL. Mato Grosso do Sul: Porto Murtinho, 04.XII.2007, *F.M. Alves* &*A.L.B.Sartori* 486 (CGMS). BOLIVIA. Tarija: O'Connor, 3 km by road W of Palos Blancos, at highest point on this part of the road from Palos Blancos to Entre Ríos, 1350 m, 22.III.2007, *M. H. Nee 54858* (MO). PARAGUAY. Alto Paraguay: Parque Nacional Defensores del Chaco, alrededores de Madrejón, 17.VII.1985, *D. R. Brunner 1235* (MO).

Chloroleucon foliolosum

BRAZIL. Mato Grosso do Sul: Porto Murtinho, 15.II.2007, *F.M. Alves* 536 &*A.L.B. Sartori* (CGMS). BOLIVIA. Santa Cruz: Cordillera, Parque Nacional Kaa-Iya del Gran Chaco, 30 km al O del campamento petrolero San José, 17.VI.1998, *A. Fuentes & G. Navarro 2438* (CTES). ARGENTINA. Jujuy: Santa Bárbara, 22.XI.1970, *A. L. Cabrera & H. A. Fabris 21110* (CTES).

Chloroleucon mangense

BOLIVIA. Chuquisaca: Luis Calvo, Iguembe, 20°27'04''S 63°46'33''W, *M. Jiménez 430* (MO).

Chloroleucon tenuiflorum

ARGENTINA. Chaco: Primero de Mayo, Colonia Benítez, 16.IX.1969, *A. G. Schultz 17139* (BAB).Corrientes: Mburucuyá, Estancia Santa Teresa, 05.VI.1954, *R. Carnevali 326* (CTES).

Formosa: Matacos, CEA Ing. Juárez, 10.X.1982, *A. O. Bordón s.n.* (CTES 406495).

DESMANTHUS

Desmanthus acuminatus

ARGENTINA. JUJUY. Dpto. Capital: Camino a al aeropuerto El Cadillal, a Palo Marcado, A. L. Cabrera 27479 (SI). SALTA: Anta, El Líbano, 10 km W sobre ruta, 15.XI.1987, C. Saravia Toledo 1460 (SI).

Desmanthus paspalaceous

ARGENTINA. Corrientes: Concepción, Paraje Rincón de Luna, entre Batel y Batelito, 01.II.1963, *E. Cano & Cámara Hernández 583* (BAB).

Desmanthus tatuhyensis* var. *brevipes

PARAGUAY. Presidente Hayes: km 54 de la ruta 9, Carlos Antonio López en dirección a Filadelfia, 09.III.2005, *R. H. Fortunato et al. 8561* (BAB). ARGENTINA. JUJUY. Dpto. Capital: Ruta 66, 10 km SE de San Salvador de Jujuy, Schinini 22370 (SI).

Desmanthus tatuhyensis* var. *tatuhyensis

PARAGUAY. Paraguarí: National Park Ybicuy, 3 km S of NW corner of park, 22.VI.1991, *E. M. Zardini & R. Velázquez 27801* (BAB).

Desmanthus virgatus

ARGENTINA. Corrientes: Capital, Laguna Brava, II-IV.1944, *T. Ibarrola 219* (BAB 70856). Jujuy: Capital, Camino al Aeropuerto El Cadillal, Arroyo Palo Marcado, 23.I.1976, A. L. Cabrera et al. 27487 (BAB). PARAGUAY. Alto Paraguay: Fortín Teniente Montanía, 05.II.2002, *E. M. Zardini & L. Guerrero 57879* (BAB).

ENTEROLOBIUM

Enterolobium contortisiliqum

PARAGUAY. Central: Compañía Jykyty, ribera Del Arroyo Ka'a ñave, 14.III.1985, *L. Pérez Et al. 453* (CTES). ARGENTINA. Salta: Rivadavia, El Colgado, 23.X.2009, *M. E. Suárez 298* (CTES); General

Güemes, trayecto por ruta provincial 11, km 20-30 entre 5 km al E de estación ferroviária de Mojotoro y Campo Santo, pasando por Betanía, 22.IV.2000, *J. A. Tolaba 2627* (CTES).

GLEDITSIA

Gleditsia amorphoides* var. *amorphoides

ARGENTINA. Corrientes: Capital, Arroyo Riachuelo, puente Pessoa, 03.VIII.1973, *M. N. Correa 5109* (BAB).

Gleditsia amorphoides* var. *anacantha

This unarmed variety was successively reported but we could find specimens of reference.

HOFFMANSEGGIA

Hoffmanseggia glauca

ARGENTINA. Santiago del Estero: Banda, El Rincón, X.1951, *no collectior s.n.* (BAB); Copo, Monte Quemado, 07.III.1986, *A. M. Molina & J. Hillfer 3044* (BAB).

INGA

Inga affinis

ARGENTINA. Chaco: La Loma, Margarita Belén, 11.I.1948, *Schultz 15101* (CTES90350).

LOPHOCARPINIA

Lophocarpinia aculeatifolia

ARGENTINA. Chaco: General Güemes, a 12 km de El Sauzalito, en dirección E hacia Nueva Pompeya, 20.XII.1990, *R. H. Fortunato et al. 1487* (BAB).

MICROLOBIUS

Microlobius foetidus* subsp. *paraguensis

ARGENTINA. Formosa: Pilcomayo, Estancia Bouvier, alrededores del puesto Lima, 28.III.1993, *R. H. Fortunato et al. 4115* (BAB).

MIMOSA

Mimosa balansae

PARAGUAY. Paraguarí:Ayo. Ñaiungua, zona Achotei, 2 km al E de la Ea. Ypoá, 07.III.2005, *R. H. Fortunato et al. 8520* (BAB).

Mimosa bifurca* var. *hassleriana

PARAGUAY. Cordillera: Al pie del cerro Tobatí, XII.1970, *A. Schinini 3661* (SI).

Mimosa bimucronata

PARAGUAY. San Pedro: Around Ybapobó, 02.III.2001, *E. M. Zardini & L. Guerrero 56455* (MO).

Mimosa candollei

PARAGUAY. Alto Paraguay: Estancia Campo Grande, al N por Línea 28, 11.V.2003, *L. Pérez de Molas & G. Navarro 8315* (BAB). ARGENTINA. Formosa: Pilcomayo, Ea. Bouvier, alrededores del puesto Lima, 28.III.1993, *R.H. Fortunato et al. 4117* (BAB).

Mimosa castanoclada

PARAGUAY. Alto Paraguay: 20 km al N del Puesto 4 de Mayo, por Línea 6, 24.X.1992, *R. H. Fortunato et al. 3621* (BAB)

Mimosa centurionis

Paraguay. Concepción: Centurión, no date, *K. Fiebrig 4001* (G).

Mimosa chacoënsis

PARAGUAY. Alto Paraguay: Palmar de las Islas, 12.III.1989, Mereles 2849 (FCQ).

Mimosa cordobensis

ARGENTINA. Córdoba: Totoral, Sierra Chica, Arroyo Chiliorco, entre Santa Catalina y Ascochinga, 26.X.1982, *A. T. Hunziker et al. 24372* (CORD).

Mimosa craspedisetosa

Bolivia. Santa Cruz: Cerro San Miguel, 09.II.1989, *L. Ramella & F. Mereles 2632* (BAB, MO).

Mimosa debilis* var. *angusta

BRAZIL. Mato Grosso do Sul: Porto Murtinho,16.II.2007, *F.M. Alves et al. 28* (CGMS)

Mimosa debilis* var. *debilis

PARAGUAY. Boquerón: 18,2 km al E de Loma Plata por ruta de acceso en direcc. a R.N. 9, 200 m s.n.m., 10.III.2005, *R. H. Fortunato & al. 8620* (BAB). ARGENTINA. Corrientes:

Mimosa detinens

PARAGUAY. Boquerón: 3 km NE of Campo Loa Projecto Tagua on the road to Fortín Toledo, 17 Feb 2002, *M.Luckow et al. 4491* (BAB). ARGENTINA. Tucumán: Trancas, 810 m, *S. Venturi 960* (CTES).

Mimosa diplotricha

ARGENTINA. Corrientes: San Miguel, III.1990, *Garino s.n.* (BAB 91743).

Mimosa distans* var. *distans

BRAZIL. Mato Grosso do Sul: Porto Murtinho,16.II.2007, *F. M. Alves et al. 269* (CGMS).

Mimosa diversipila* var. *subglabriseta

Paraguarií: 1 kmN of Villa Florida on Tebicuary River, 25 May 1993, *Zardini and Guerrero* 35827 (BAB)

Mimosa diversipila* var. *diversipila

PARAGUAY. *Cordillera:* Tobatí, Ybytú Silla Mesa, 03 Mar 1991, *Zardini and Velázquez* 26739 (BAB).

Mimosa dolens* subsp. *callosa

ARGENTINA. Corrientes: Ea. Millán, R17, 7 km E de Santa Rosa, 30.XI.1978, *M. M. Arbo & S. Ferrucci 2165* (CTES 91866).

Mimosa dolens* subsp. *rigida* var. *foliolosa

ARGENTINA. Corrientes: San Miguel, Ruta Provincial 118, a 54 km de la intersección con RN12, 07.III.2007, *M. Morales & G. Seijo 277* (BAB).

Mimosa dolens* subsp. *acerba* var. *latifolia

ARGENTINA. Corrientes: Mburucuyá, Cerca Mburucuyá, Ea. Sta. Teresa, 30.IX.1990, *R. H. Fortunato et al. 1797* (BAB).

Mimosa ephedroides

ARGENTINA. La Rioja: Los Llanos, Barreal, 19.II.1940, *A. Castellanos s.n.* (BA 33672).

Mimosa farinosa

ARGENTINA. Santiago del Estero: Guasayán, Ruta 64, sierra de Guasayán, 24 Oct 1988, *Pérez-Moreau and Petetin s.n.* (BAB)

Mimosa gracilis* var. *leiocarpa

PARAGUAY. Central:Estero del Ypoá. Villeta - Puerto Guyrati. 7 km S of Villeta, 18.XI.1992, *E. M. Zardini & T. Tillería 33667* (MO).

Mimosa guaranitica

PARAGUAY. Cordillera: Altos, 23.I.1903, *K. Fiebrig 784* (LIL 54416).

Mimosa hexandra

PARAGUAY. Central: Puesto de Peaje Emboscada, ca. del Río Salado, 25º07'S 57º26'W, 6.III.2008, fr, *R, H. Fortunato et al. 9176* (BAB).

Mimosa invisa

ARGENTINA. Chaco: Antequera, 27.XII.1942, *A. G. Schulz 3754* (SI).

Mimosa morongii

PARAGUAY. Central: On grand campo near Luque, no date, *T. Morong 728* (NY).

Mimosa oligophylla

ARGENTINA. Corrientes: Mburucuyá, Parque Nacional Mburucuyá, 28-XII-2004, M. S. Ferrucci *et al.* 2198 (BAB)

Mimosa petraea

PARAGUAY. Paraguarí: Piraretá – Piribebuy, VI.1969, *A. Schinini* (SI).

Mimosa pigra* var. *pigra

ARGENTINA. Formosa: Pilagá, costa del río Paraguay, 12 km desde Mojón

Mimosa pigra* var. *dehiscens

PARAGUAY. Paraguarí: Esteros del Ypoá, 25°37'S 57°24'W, 18.III.1992, *E. M. Zardini & T. Tilleria 31267* (BAB).

Mimosa polycarpa* var. *subandina

BOLIVIA. Chuquisaca: El Salvador, Cimboc, 06-V-1992, Nelson Joaquín 10879 (BAB). ARGENTINA. Jujuy: El Carmen, Alrededores de Perico, 2-V-1992, R. H. Fortunato 3806 (BAB).

Mimosa polycarpa* var. *spegazinii

ARGENTINA. Formosa: Patiño, Estanislao del Campo, X.1972, *P. Insfrán 1175* (BAB).

Mimosa pseudopetiolaris

PARAGUAY. Paraguarí: Between Nueva Italia and Carapeguá, 30.5 km SE of Nva. Italia, 14.XII.1989, *E. M. Zardini & C. Velázquez 16812* (MO 4605118).

Mimosa sensibilis* var. *sensibilis

BRAZIL. Mato Grosso do Sul: Porto Murtinho, 08.V.2007, F.M. Alves *et al.*341 (CGMS). PARAGUAY. Alto Paraguay: Parque Nacional Defensores Del Chaco: Cerro León, 17.V.2003, *L. Pérez & G. Navarro 8902* (BAB).

Mimosa somnians* var. *somnians

PARAGUAY. Central: Tavarory, 1 km Southeast from entrance, 25º28’24’’S 57º31’56’’W, 16.VI.1994, fl-fr, *E. M. Zardini & M. Vera 39501* (BAB).

Mimosa strigillosa

BRAZIL. Mato Grosso do Sul: Rio Paraguai, próximo do Porto de Embarque, 15-III-2004, G. Hatschbach *et al.* 77176 (BAB, MBM293913). PARAGUAY. *Dpto. Alto Paraguay:* Mayor Pedro Lagerenza, cauce seco del río Timane, 04-IV-1978, A. Schinini & E. Bordas 14861 (CTES91831). ARGENTINA. *Tucumán: Dpto. Capital*, río Salí, 15-XI-1927, S. Venturi 5625 (MO960016).

Mimosa subsericea

ARGENTINA. Corrientes: San Miguel, RP118, a 2 km del cruce con RN12, 07.III.2007, *M. Morales & G. J. Seijo 266* (BAB).

Mimosa tobatiensis

PARAGUAY. CORDILLERA: Tobatí, 09.I.1951, fl, *Sparre & Vervoorst 1504* (Paratipo: BAB).

Mimosa troncosoae

PARAGUAY.ALTO PARAGUAY. Parque Nacional Defensores del Chaco, Cerro León, 20º30’20’’S 60º05’20’’W, 17.V.2003, *L. Pérez de Molas & G. Navarro 8890* (BAB)

Mimosa tweedieana

ARGENTINA. Chaco: Primero de Mayo, río Tragadero y camino a Antequeras, 02.V.1994, *G. Seijo 1015* (BAB).

Mimosa xanthocentra* var. *mansii

ARGENTINA. Formosa: Patiño, 33 km NNW de Las Lomitas, por ruta 32, 30.III.1992, *R. H. Fortunato et al. 3435* (BAB).

Mimosa xanthocentra* var. *xanthocentra

PARAGUAY. Concepción: Camino a Paso Mbutú, Karaza Bola, no date, *R. H. Fortunato et al. 845* (BAB). ARGENTINA. Jujuy: Capital, alrededores de la capital, pasando Pta. Pérez, II-1936, A. G. Schulz 2016 (SI).

MIMOZYGANTHUS

Mimozyganthus carinatus

ARGENTINA. Chaco: Almirante Brown, ruta nacional 16, 66 km NO de Monte Quemado, 06.III.1986, *A. M. Molina & J. Hifler 2961* (BAB). JUJUY: San Pedro de Jujuy, camino de San Pedro a Ledesma, ±20 km de San Pedro de Jujuy, 04.XI.1971, *P. R. Legname et al. 8677* (CTES).

NEPTUNIA

Neptunia plena

PARAGUAY. Presidente Hayes: Ea. Zalazar, Laguna Zalazar, scrub on salt flats and on border of lake and patche of dry forest, 90 m, 12-XII-1996, *E. M. Zardini & L. Guerrero 45909* (BAB).

Neptunia pubescens

ARGENTINA. Formosa: Patiño, 20 km al O de Ibarreta por Antigua ruta 81, 12.XI.1983, *R. H. Fortunato et al. 490* (BAB).

PARAPIPTADENIA

Parapiptadenia rigida

PARAGUAY. Cordillera: Caacupé, 53 km de Asunción, cerro, *L. Pérez 225* (BAB). Paraguarí: Cerro Mbatoví, 29.V.1983, *E. M. Zardini & N. Soria 4426* (BAB).

Parapiptadenia excelsa

PARAGUAY. Central: Acosta Ñu, affluent of Río Paraguay, 25°26’S 57°32’W, 10.VI.1993, *E. M. Zardini & L. Guerrero 36225* (BAB); Acosta-Ñú Creek, 10-VI-1993, *E. M. Zardini & L. Guerrero 36225* (BAB).

PARKINSONIA

Parkinsonia aculeata

ARGENTINA. Chaco: Barranqueras, 15.XI.1913, *Muniez Et al. 31* (BAB 92042). Salta: Rivadavia, Ruta 81, 3 km W de Las Blancas, 15.IX.1980, *M. Correa et al. 7555* (BAB).

PELTOPHORUM

Peltophorum dubium

ARGENTINA. Santa Fe: Puerto Ocampo, 13-XI-1941, *Ragonese s.n.* (BAB72879). BRAZIL. Mato Grosso do Sul: Porto Murtinho, 5.XII.2007, F.M.Alves & A.L.B.Sartori 500 (CGMS).

PIPTADENIOPSIS

Piptadeniopsis lomentifera

PARAGUAY. Boquerón: 2 km N of Buena Vista, on the road to Laguna Capitán, 22°33’09’’S 59°42’45’’W, 18.II.2002, *M. Luckow et al. 4505* (BAB). Presidente Hayes: Between the Río Verde and Tacuara, 14.XII.1996, *E. M. Zardini & M. Vera 46115* (BAB).

PLATHYMENIA

Plathymenia reticulata

BOLIVIA. Santa Cruz: Ángel Sandoval, Santo Corazón, ex-8, 18°24’S 55°00’W, nacientes Del rio Santo Corazón, 7 km NO de Santo Corazón, cerro aislado com cerrado em La cima, 01.V.1997, *A. F. Fuentes et al. 1822* (MO).

PROSOPIDASTRUM

This genus has been mentioned for the Bolivian Chaco, but we failed to find representative specimens. In Argentina this genus does not occur in Gran Chaco, but in sorrounding Monte ecoregion.

PROSOPIS

Prosopis abbreviata

ARGENTINA. Santiago del Estero: Ojo de Agua, km 80, campos al oeste de Sierra de Ambargasta, 17.XII.1981, *E. A. Ulibarri 1346* (SI).

Prosopis affinis

ARGENTINA. Corrientes. San Luis del Palmar: 1km W de San Luis del Palmar, 12-IX-1995, *A. Schinini et al. 31295* (BAB). Formosa: Patiño, Pozo Navagán, reducción de indígenas pilagás, 18.I.1982, *P. Arenas 1957* (CTES).

Prosopis alba* var. *alba

PARAGUAY. Ñeembucú: Paraje costa paraguaya, frente a la boca del río Bermejo, zona Pilar, 14.XII.1950, *A. G. Schulz 7834* (CTES). ARGENTINA. Salta: Chicoana, Las Moras, ruta 68 km 145, 5-6 km N de Osma, entre El Carril y Moldes, 1150 m, 26.XI.2005, *J. A. Tolaba & M. Fabbroni 3728* (CTES). Santiago del Estero: Copo, Matoque, 18.X.1947, *F. E. Luna 343* (CTES).

Prosopis alba* var. *panta

PARAGUAY. Alto Paraguay: Kilómetro 160, Sector Puerto Casado, X.1938, *T. Rojas 8452* (SI). ARGENTINA. Salta: Anta, Pozo Largo, 10 km SSE de Joaquín V. González, 02.X.1987, *C. Saravia Toledo* 1430 (CTES).

Prosopis campestris

ARGENTINA. Córdoba: Pampa de Pocho, 16.XII.1963, *Ragonese & Piccinini 9124* (CTES101351).

Prosopis chilensis

ARGENTINA. La Rioja: Chamical, ciudad de Chamical, aledaños de la Estación de FFCC, 27.X.1988, *F. Biurrun & E. Pagliari 2506* (CTES145829). San Luis: General Belgrano, Villa General Roca, en inmediaciones del embalse homónimo, 27.XII.1994, *L. A. Del Vitto et al. 8291* (CTES).

Prosopis elata

ARGENTINA. Salta: Anta, Los Colorados, 110 km NNE de Joaquín V. González, 21.XII.1986, *C. Saravia Toledo 1268*[a] (CTES135777).

NOTE: We could not find material of this species in Sierra Chaco but it grows in the adjacent foothills, and its occurrence there is probable.

Prosopis fiebrigii

PARAGUAY. Presidente Hayes: Fuerte Gral. Bruguez, 03.I.1980, *P. Arenas* (CTES ex BACP 1548).

Prosopis flexuosa

ARGENTINA. La Rioja: Belgrano, entre Chamical y Chañar a 20 m del primer campo experimental INTA Las Vizcacheras; alrededores del jardín de introducción, 26.X.1988, *F. Biurrun 2504* (CTES147938). San Luis: La Capital, El Chorrillo, 17.I.1989, *L. A. Del Vitto 3317* (CTES).

Prosopis hassleri

PARAGUAY. Alto Paraguay: Fortín Madrejón, 06-X-1979, *A. Schinini & E. Bordas 18147* (BAB). ARGENTINA. Formosa: Pilcomayo, ruta 86 km 75, 09.XI.1948, *I. Morel 6560* (CTES 101421).

Prosopis hassleri* var. *nigroides

ARGENTINA. Santa Fe: General Obligado, Estancia Las Camelias, 15.I.1937, *A. Ragonese 2423* (SI).

Prosopis kuntzei

ARGENTINA. Chaco: General Donovan, La Escondida, predio de la empresa Indunor, 15.X.2014, *W. A. Medina 815* (CTES). General Güemes, camino de La Esperanza a Taco Pozo, 30.X.1986, *A. Schinini & S. M. Pire 24917* (CTES). Tucumán: Leales, 3 km antes de Estación Tacanas, 06.III.1966, *Vaca & Villa Carenzo 2806* (CTES).

Prosopis pugionata

ARGENTINA. Córdoba: Camino entre La Majadilla y La Esperanza, al pie de la ladera occidental de la Sierra Chica del Norte, XI.1951, *M. Sayago 1685* (CTES). La Rioja: Banda del Cura, 09.XII.1957, *Ruiz Leal & F. A. Roig 18860* (CTES15001).

Prosopis nigra

BOLIVIA. Chuquisaca: Luis Calvo, 13.5 km S de Carandaity, camino a Campo Azul, 08.IV.1993, *C. Saravia Toledo et al. 11470* (CTES). PARAGUAY. Presidente Hayes: Colonia Paratodo, 18.IX.1993, *R.*

Degen & F. Mereles 2998 (CTES). ARGENTINA. Formosa: Estancia “Guaycolec”, 25 km N de la ciudad de Formosa, sobre ruta nacional 11, 30.X.1989, *G. Placci & S. Arditi* (CTES).

Prosopis nigra **var.** ***ragonesei***

ARGENTINA. Santa Fe: Vera, no date, *A. Burkart 5933* (SI).

Prosopis nigra **var.** ***longispina***

ARGENTINA. Corrientes: Capital, 23.IX.1954, *T. M. Pedersen 2808* (SI).

Prosopis nuda

PARAGUAY. Boquerón : Mariscal Estigarribia, 16.IV.1978, *Schinini & Bordas 15222* (CTES).

Prosopis reptans

ARGENTINA. La Rioja: R. V. Peñaloza, 31°02’S 67°06’W, 13.XI.1970, *Piccinini et al. 1672* (CTES 235342).

Prosopis **Rojasiana**

PARAGUAY. Boquerón: Mariscal Estigarribia, 16.IV.1978, *A. Schinini & E. Bordas 15222* (CTES).

Prosopis rubriflora

BRAZIL. Mato Grosso do Sul: Porto Murtinho, 20.XI.2008, *E.P.Seleme&A.L.B.Sartori* 139 (CGMS).

NOTE: This species was reported from Amambay department in Paraguay (Fortunato et al. 2008), which is ecologically included mainly in Cerrado and Paranaense ecoregions. We had not opportunity to see material from Amambay of this species. Thus, its endemicity in Gran Chaco could be discussed if new records or specimens are found in the future.

Prosopis ruscifolia

BOLIVIA.Santa Cruz: Ñuflo de Chávez, región del Lomerío, Comunidad Salinas, 31.I.1994, *M. Toledo & I. Surubí 358* (CTES263438).PARAGUAY. Presidente Hayes: Ruta a General Díaz, desvío a Colonia Nivaclé, 24.IX.1993, *F. Mereles & R. Degen 5405* (CTES223266). ARGENTINA. Chaco: 5 km S de Resistencia, 24.IX.1986, *Pire 2304* (CTES).

Prosopis sericantha

ARGENTINA. Formosa: Ingeniero Juárez, 07.XI.1962, *Bordón 41* (CTES104547).

Prosopis strombulifera

ARGENTINA. Tucumán: Tafí, Tipunco, ruta 307, 7 km N de Amaicha del Valle, entre mojones 125 y 126; camino vecinal, 11 km desde la ruta, 22.II.2000, *L. Anzoategui & L. Mautino 363* (CTES 319966).

Prosopis torquata

ARGENTINA. Córdoba: Punilla, Las Chacras. 06.XII.1958, *J. B. Rossi s.n.* (CTES). Salta: Between Macapillo and N. S. de Talavera, 11.XI.1974, *T. M. Pedersen 10830* (CTES).

Prosopis vinalillo

PARAGUAY. Presidente Hayes: 65 km sobre ruta a General Díaz, camino a Estancia Casamada, 21.IX.1993, *R. Degen & F. Mereles 2991* (CTES227025). ARGENTINA. Chaco: Primero de Mayo, Colonia Benítez, XII.1956, *A. G. Schulz 1373* (CTES). Tucumán: Capital, río Salí, 450 m, 03.III.1925, *S. Venturi 3879* (BAB).

PELTOPHORUM

Pelthophorum dubium

ARGENTINA. Formosa: Formosa, Arroyo Mirador, ruta 11 antigua, 16.III.1979, *B. G. Piccinini & C. A. Petetin 3620* (BAB).

PTEROGYNE

Pterogyne nitens

ARGENTINA. Chaco: Bermejo, General Vedia, II.1928, *Schulz s.n.* (BAB 55324).

SENNA

Senna aculeata

PARAGUAY. Cordillera: Río Salado, 11.III.1988, *I. Basualdo 1452* (MO).

Senna alata

ARGENTINA. Formosa: Pilcomayo, Isla Buey Muerto, costa sobre el río Paraguay, 26.III.1993, *R. H. Fortunato et al. 4041* (BAB).

Senna aphylla* var. *aphylla

ARGENTINA. Córdoba: Río Primero, Santa Rosa por Socorro, 05.XI.1952, *V. J. Mazzucconi 143* (BAB). Santiago del Estero: Ojo de Agua, arroyo Lito, 12.II.1996, *R. L. Pérez Moreau et al. 3827* (BAB).

Senna bicapsularis

ARGENTINA. Chaco: Primero de Mayo, Camino al Cerrito, 19.IV.2001, *V. Solís Neffa 451* (BAB).

Senna cernua

BOLIVIA. Chuquisaca: Hernando Siles, Canton Rosadio del Ingre, comunidad Villa Hermosa, 01.I.2008, *A. Flores 39* (MO).

Senna chacoënsis

ARGENTINA. Santiago del Estero: Copo, ruta 5, 10 km S de Monte Quemado, 05.III.1986, *A. M. Molina & J. Hifler 2699* (BAB).

Senna chloroclada

BOLIVIA. Santa Cruz: Cordillera, Camiri, 70 km hacia Yacuiba, 03.X.1983, *S. G. Beck & M. Liberman 9389* (SI). ARGENTINA. Chaco: Comandante Fernández, E.E.A. INTA Sáenz Peña, 21.XII.1983, *A. M. Molina & M. Sánchez 2499* (BAB); General Güemes, Paraje San Sebastián, 3 km de El Colorado hacia el N por ruta a Wichi, 17.XI.1980, *R. H. Fortunato et al. 1398* (BAB).

Senna corymbosa

ARGENTINA. Córdoba: Colón, Sierra Chica (Falda E) : Entre Cabana y Los Quebrachitos, 24.V.1964, *A. T. Hunziker 17432* (MO). Formosa: Estancia Guaycolec, 22 km N de Formosa, sobre ruta 11, 15.II.1995, *M. G. López Et al. 90* (BAB).

Senna hirsuta* var. *puberula

PARAGUAY. Paraguarí: Estero Del Ypoá, Paraguarí – Carapeguá, Arroyo Mbaey, 27.I.1993, *E. Zardini & T. Tillería 34902* (BAB). ARGENTINA. Tucumán: Famaillá, San Ramón, Bella Vista, 450 m, 20.I.1924, *S. Venturi 2753* (BAB).

Senna hirsuta* var. *leptocarpa

PARAGUAY. Central: Asunción, Barrio Santísima Trinidad, 07.I.1981, *P. Arenas s.n. 5* (SI).

Senna morongii

ARGENTINA. Chaco: Presidencia La Plaza, Parque Nacional Chaco, 25.XI.1990, *R. H. Fortunato et al. 1675* (BAB). Santiago Del Estero: Guasayán, sierra de Guasayán y ruta 34, 14.V.1983, *A. M. Molina et al. 1463* (BAB).

Senna obtusifolia

ARGENTINA. Formosa: Laishi, camino entre Tatané (antigua Ruta 1) y Herradura (Río Paraguay), 12.III.1979, *B. G. Piccinini & C. A. Petetín 3525* (BAB); Matacos, Ing. Juárez, Barrio Obrero, orillas del pueblo, 27.II.1983, *P. Arenas 2354* (BAB).

Senna occidentalis

BRAZIL. Mato Grosso do Sul: Porto Murtinho, 04.XII.2007, *F.M.Alves&A.L.B.Sartori* 491 (CGMS). ARGENTINA. Formosa: Bermejo, El Silencio, a orillas del Teuco, frente a Sauzalito, 19.II.1990, *R. H. Fortunato 1475* (BAB). Tucumán: Leales, 43 km al SE de Sna Miguel de Tucumán, 03.V.1981, *R. Rossow et al. 572* (BAB).

Senna pendula* var. *glabrata

PARAGUAY. Central: Itá, 29.IV.1984, *W. J. Hahn 2365* (MO).

Senna pendula* var. *paludicola

PARAGUAY. Presidente Hayes: Around Pozo Colorado, 02.XI.2001, *E. M. Zardini & M. Vera 57618* (BAB). ARGENTINA. Formosa: Pilcomayo, Estancia Bouvier, alrededores del riacho Jodido, 25.III.1993, *R. H. Fortunato et al. 3960* (BAB).

Senna pilifera

BRAZIL. Mato Grosso do Sul: Porto Murtinho, 29.VIII.2007, F.M.Alves *et al.* 466 (CGMS)

Senna praeterita

BOLIVIA. Santa Cruz: Cordillera, Charagua, 5 km N del pueblo, trayecto del Río Ovai, 13.IV.1990, *I. G. Vargas 479* (MO).

Senna rugosa

BOLIVIA. Santa Cruz: Cordillera, Chovoreca, no date, *W. J. Hahn 1590* (MO).

Senna scabriuscul*a*

ARGENTINA. Corrientes: Mburucuyá, between Mburucuyá and Manantiales, 15.I.1953, *T. M. Pedersen 1923* (MO).

Senna spectabilis

BOLIVIA. Santa Cruz: Cordillera, Cerca Cuevo adentro la sierra por la planchada antigua de Chevron, 29.XII.1994, *A. Jardim & N. Rosas-Hurtado 1525* (MO). Tarija: Gran Chaco, 4 km N of center of Villa Montes, along dirt road and gas pipeline, 30.V.2005, *Nee 53262* (MO). PARAGUAY. Central: Jardín Botánico y Zoológico, Trinidad, Asuncion. Reserva Natural, 20.VI.1990, *B. Pérez 26* (MO).

Senna spiniflora

ARGENTINA. Formosa: Matacos, Ing. Juárez, 3 km N del pueblo, 25.II.1983, *P. Arenas 2331* (BAB).

Senna subulata

ARGENTINA. Jujuy: Santa Bárbara, Santa Clara, 16.II.1995, *N. B. Deginani & A. M. Cialdella 911* (MO).

Senna trichosephala

ARGENTINA. Santiago del Estero: Ojo de Agua, 30.XI.1944, *B. Balegno 211* (BAB 71759).

STENODREPANUM

Stenodrepanum bergii

ARGENTINA. Santiago del Estero: Jiménez, ruta 37, 17 km S de Las Delicias, 19.V.1983, *A. M. Molina et al. 1861* (BAB).

ZAPOTECA

Zapoteca formosa

PARAGUAY. Cerro León, 20°20'S 60°25'W, oeste del cerro, 13.XI.1992, L. R. Ramella et al. 2975 (BAB). ARGENTINA. Jujuy: Santa Bárbara, 12.XII.1998, *O. Ahumada 8925* (SI).

ZYGIA

Zygia morongii

PARAGUAY. Cordillera: Road from Limpio to Arroyos y Esteros at río Piribebuy, 20.VII.1995, *L. R. Landrum 8595* (CTES).

Zygia pithecolobioides

BOLIVIA. Santa Cruz: Germán Busch, 18°30’S 59°00’W, no date, *R. Guillén 5107* (BOL). ARGENTINA. Chaco: Primero de Mayo, 10.XI.1936, *T. Meyer 2131* (SI).

Papilionoideae

ACOSMIUM

Acosmium cardenasii

PARAGUAY. Alto Paraguay: Línea 28, próximo a Ea. Choroveca, 27.VII.2002, *F. González Parini 1043* (FCQ).

ADESMIA

Adesmia bicolor

ARGENTINA. Corrientes: Esquina, VI.1906, *J. Bolla s.n.* (BAB 16248).

Adesmia cordobensis

ARGENTINA. Córdoba: Ischilín, ruta provincial 17 al E de Ongamira (Sierra Chica), 19.I.2004, *R. Pozner & M. J. Belgrano 312* (MO, SI).

Adesmia macrostachya

ARGENTINA. Córdoba: Colón, Ascochinga, 17.XI.1935, *Giardelli 164* (SI). Santa Fe: Colastiné: Capital, IX-1983, *M. N. Correa 8788* (BAB); General Obligado, NE de Reconquista, Más o menos rara. Pajonal cerca del palmar de Copernicia, 09.XI.1933, *A. Burkart 5884* (SI).

Adesmia muricata

ARGENTINA. Chaco: Comandante Fernández. EEA Sáenz Peña, alrededores, 16-XII-1983, *A. M. Molina & M. Sánchez 2392* (BAB). Formosa: Matacos, Ing. Guillermo Juárez, 33 km al S por ruta 39, 07.XI.1983, *R. H. Fortunato et al. 312* (BAB). Tucumán: Estancia Sacrificios, Estación Graneros, 11-XII-1925, *S. Venturi 1558* (BAB40847).

Adesmia muricata* var. *dentata

ARGENTINA. Chaco: Primero de Mayo, Ea. Varela, 25.XI.1950, *A. G. Schultz 7637* (BAB); Mayor L. J. Fontana, Enrique Urién, 15.XI.1959, *A. G. Schulz 10847* (SI). Córdoba: V. Reartes, 1917-1919, *Castellanos 27* (SI). Tucumán: Leales, La Pirhua, X.1919, *S. Venturi 527* (SI).

Adesmia muricata* var. *gilliesii

ARGENTINA. Córdoba: Ascochinga, 03.X.1936, *E. G. Nicora 1011* (SI).

NOTE: Ulibarri and Burkart (2000) suggest that *A. Muricata* var. *Muricata* is restricted to Pampa and Patagonia region and consequently that should not occur in Gran Chaco. This variety is indicated for Argentine provinces of Gran Chaco in Fortunato et al. (2008) updated in Zuloaga et al. 2017. We included this variety here but doubtfully since we did not see material from this region.

AESCHYNOMENE

Aeschynomene americana

ARGENTINA. Chaco: *General Donovan*, 2 Km N de Makallé, 19-II-1980, Schinini *et al.* 20024 (CTES).

Aeschynomene denticulata

ARGENTINA. Corrientes: General Paz, Capillitas, 8-II-1979, T. M. Pedersen 12390 (CTES).

Aeschynomene falcata

ARGENTINA. Formosa: Pilcomayo, Ruta 86, Km 52, 5-IV-1948, I. Morel 5337 (CTES).

Aeschynomene histrix

BOLIVIA. Santa Cruz: Cordillera, 6 km S de Lagunillas, 16.IV.1977, *Krapovickas Et al. 31424* (CTES). ARGENTINA. Corrientes: Berón de Astrada, 15 Km W de Itá ibaté, arroyo Santa Isabel, 16-I-1977, Schinini *et al.* 14097 (CTES).

Aeschynomene mollicula

BOLIVIA. Santa Cruz: Chiquitos, Parque Nacional Kaa-Iya del Gran Chaco18º29´20"S, 61º7´6"W, 17, VI, 1998, Fuentes *et al.* 2440 (CTES).

Aeschynomene montevidensis

BOLIVIA. Santa Cruz: Chiquitos, Parque Nacional Kaa-Iya del Gran Chaco18º29´20”S, 61º7´6”W, 17, VI, 1998, Fuentes *et al.* 2440 (CTES). ARGENTINA. CORRIENTES: *Esquina*, Ruta 27, 3 Km N de Esquina, 1-XII-1974, Krapovickas *et al.* 26978 (CTES).

Aeschynomene paraguayensis

PARAGUAY. Paraguarí:Chololó, 14.II.1969, T. M. Pedersen 9301 (CTES).

Aeschynomene parviflora

ARGENTINA. Formosa: Pilcomayo, Riacho Negro, 14-V-1947, Morel 2826 (CTES).

Aeschynomene rudis

ARGENTINA. Chaco: Primero de Mayo, Colonia Benítez, III-1956, A. Schulz 9061 (CTES)

Aeschynomene sensitiva

ARGENTINA. Corrientes: San Cosme, Ruta 12, 3 Km W de San Cosme, 20-III-1975, Arbo *et al.* 709 (CTES).

Aeschynomene viscidula

Paraguay. Presidente Hayes: Estancia Zalazar, 12.II.1993, *L. Pérez et al. 2877* (CTES). ARGENTINA. Formosa: Patiño, Las Lomitas, ruta 28, 6 Km al N de las Lomitas, II-1972, Insfran 995 (CTES).

AMBURANA

Amburana cearensis

BRAZIL. Mato Grosso do Sul: Porto Murtinho, 08.V.2007, F.M. Alves *et al.* 342 (CGMS). PARAGUAY. Chaco, Cerro León, 24.VIII.1981, *A. Schinini et al. 21141* (CTES).

APURIMACIA

Apurimacia dolichocarpa

ARGENTINA. Córdoba: Entre Tanti y Taminga, no date, *A. Ragonese 9297* (BAB 77953).

ARACHIS

Arachis batizocoi

BOLIVIA. Santa Cruz: Cordillera, Ipati, 16.IV.1977, *A. Krapovickas et al. 30080* (BAB).

Arachis correntina

ARGENTINA. Corrientes: Capital, ruta 12, 1 km S puente sobre el río Riachuelo, 18.VI.2002, *G. Seijo et al. 2885* (BAB).

Arachis duranensis

ARGENTINA. Salta: Anta, Cabeza de Anta, ruta 5 y puente sobre arroyo, 09.V.2002, *G. Seijo & Solís Neffa 2849* (BAB); Capital, Barrio El Milagro, 11.V.2002, *G. J. Seijo & V. G. Solís Neffa 2854* (CTES).

Arachis glabrata* var. *glabrata

PARAGUAY. Paraguarí: Road to Yeré, near río Tebicuary, 24.II.1994, *E. M. Zardini & L. Guerrero 38617* (BAB).

Arachis glabrata* var. *hagenbeckii

ARGENTINA. Corrientes: Mburucuyá, cerca de Mburucuyá, estancia Santa Teresa, propiedad del Sr. Pedersen, 29.XI.1990, *R. H. Fortunato et al. 1781* (BAB).

Arachis hassleri

PARAGUAY. Concepción: A 20 km ao nordeste da cidade de Concepción, na periferia da cidade de Loreto, 02.II.1997, *G. P. Silva Et al.3818* (CTES).

Arachis lignosa

BRAZIL. Mato Grosso do Sul: Porto Murtinho, 05.XII.2007, *F. M. Alves & A. L. B. Sartori 504* (CGMS).

Arachis microsperma

BRAZIL. Mato Grosso do Sul: Porto Murtinho,24.II.2011,*T.G.Freitas & C.S.Souza45* (CGMS).

Arachis nitida

BRAZIL. Mato Grosso do Sul: Fazenda Retiro Conceição, 27.II.2010, fl., *C.S. Souza et al. 17* (CGMS); Fazenda Santo Antônio, cerca de 5 km oeste da rodovia BR-267, na estrada vicinal de acesso à Fazenda Amonguijá, 16.II.2007, fl., *V.J. Pott 9099* (CGMS).

Arachis paraguariensis

ARGENTINA. Córdoba: Estación Experimental Agropecuaria INTA Manfredi, cultivo procedente de Paraguay: San Bernardino, 16.III.1988, *A. Krapovickas 42276* (BAB).

ASTRAGALUS

Astragalus distinens

ARGENTINA. San Luis: Pedernera, Ruta 148, camino a El Morro, 17.XI.2016, *M. Morales et al. 1778* (BAB). Santa Fe: Vera, próximo a Laguna El Palmar, 23.IX.1983, *R. H. Fortunato 192* (BAB). Santiago del Estero: Figueroa, Salitral El Saladillo, ruta provincial 5, 18.XI.1978, *B. G. Piccinini & C. A. Petetin 3020* (BAB).

CALOPOGONIUM

Calopogonium sericeum

ARGENTINA. Formosa: Ea. Bouvier, Puesto Santa Rosa, Reserva de Flora y Fauna, 20.XI.1991, *R. H. Fortunato et al. 2486* (BAB).

CAMPTOSEMA

Camptosema ellipticum

BRAZIL. Mato Grosso do Sul: Porto Murtinho, 25.VIII.2004, *G.P.Nunes et al. 80* (CGMS). BOLIVIA. Santa Cruz de la Sierra: Alto Parapetí, 08.I.1982, *R. de Michel 147* (BAB).

Camptosema paraguariense* var. *paraguariense

ARGENTINA. Chaco: Primero de Mayo, Campo Antequera, Laguna La Mora, 25.XI.1966, *A. G. Schulz 9627* (BAB).

Camptosema paraguariense* var. *parviflorum

ARGENTINA. Chaco: San Fernando, 9 km de Basail, hacia el N por ruta nacional 11, 12.XI.1990, *R. H. Fortunato et al. 1223* (BAB).

Camptosema praeandinum

ARGENTINA. Salta: Rosario de Lerma, Quebrada del Toro, 7 km al N de Campo Quijano, ingresando a la quebrada, 06.III.2001, *R. H. Fortunato et al. 6936* (BAB).

CANAVALIA

Canavalia brasiliensis

ARGENTINA. Corrientes: San Cosme, costa del río Paraná, 20.VI.1973, *A. Krapovickas & C. L. Cristóbal 23637* (BAB).

Canavalia ensiformis

PARAGUAY. Alto Paraguay: Chaco, Cerro León (Lagerenza), oeste del cerro, 18.XI.1992, *L. Ramella et al. 3092* (BAB). ARGENTINA. Formosa: Pozo del Tigre, IX.1934, *T. Meyer 6981* (SI).

Canavalia mattogrossensis

PARAGUAY. Paraguarí: National Park Ybicu'í, 22.vi.1991, *E. Zardini & H. Velázquez 27881* (BAB).

CENTROSEMA

Centrosema angustifolium

BOLIVIA. Santa Cruz: Cordillera, Parque Nacional Kaa-Iya, Fortín Ravelo, 08.II.1998, *A. F. Fuentes & G. Navarro 2226A* (MO).

Centrosema kermesi

ARGENTINA. Chaco: río Bermejo, 23.I.1901, *E. Kermes 539* (SI).

Centrosema pasquorum

ARGENTINA. Formosa: Nuevo Porteño, 26.I.1972, *A. G. Schultz 17980* (CTES 137460).

Centrosema sagittatum

ARGENTINA. Corrientes: San Cosme a Itatí, VII.1938, *A. G. Schultz 1177* (CTES 137360). Jujuy: Santa Bárbara, 1 km al S de La Quinta, 07.III.1983, *J. H. Hunziker et al. 10642* (SI).

Centrosema virginianum

ARGENTINA. Chaco: Colonia Benítez, en cultivo proveniente de El Colorado, crece a orillas del río Bermejo, Estación Experimental del Colorado, 9.III.1963, *A. G. Schultz 12188* (CTES 137298). Jujuy: Santa Bárbara, 12.XII.1998, *O. Ahumana 8943* (SI).

CHAETOCALYX

Chaetocalyx brasiliensis

ARGENTINA. Chaco: Bermejo, Pto. Las Palmas, 26-IV-1985, Schinini *et al.* 24329 (CTES).

Chaetocalyx chacoensis

BOLIVIA. Santa Cruz: Cordillera, Parque Nacional Kaa-Iya, del Gran-chaco Paleodunas, 25 Km al NE de Palmar de las Islas, 19º 39´S, 60º 25´30´´ W, 12-II-1998, *Fuenteset al. 2383* (CTES, USZ).

Chaetocalyx latifolia* var. *setuligera

Tarija: *Gran Chaco*: Quebrada de Chimeo, 8 Km N de Villa Montes, camino a Camiri, 6-V-1983, Krapovickas *et al.* 39231(CTES).

Chaetocalyx latifolia* var. *latifolia

ARGENTINA. Corrientes: Empedrado, Estancia Las Tres Marías, 5-IV-1979, *Vanni 55* (CTES, ICN, F, MEXU, MO).

Chaetocalyx longiflora

ARGENTINA. Corrientes: *San Cosme*: San Cosme, 20-VI-1973, Krapovickas *et al.* 23684 (CTES).

CLITORIA

Clitoria falcata

PARAGUAY. Cordillera: Tobatí, "Ybytú Silla" mesa, 09.III.1991, *E. M. Zardini & R. Velázquez 27192* (BAB).

Clitoria cordobensis

ARGENTINA. Córdoba: Salsipuedes, VI.1939, *R. Lahitte s.n.* (BAB 60292).

Clitoria epetiolaris

ARGENTINA. Corrientes: Mburucuyá, cerca de Mburucuyá, estância Santa Teresa, propiedad Del Sr. Pedersen, 29.XI.1990, *R. H. Fortunato Et al. 1772* (BAB).

COCLIASANTHUS

Cocliasanthus caracalla

PARAGUAY. Paraguarí : Parque Nacional Ybycuí, camino que conduce al límite del parque hacia Mboyacapucú, 06.X.1984, *L. Pérez 368* (CTES115013).

COLLAEA

Collaea argentina

ARGENTINA. Córdoba: Punilla, subida al cerro La Banderita, 08.X.2009, *H. G. Bach et al. 297* (BAB).

Collaea stenophylla

ARGENTINA. Chaco: Isla del Cerrito, 10.X.1971, *A. Krapovickas & C. L. Cristóbal 20060* (BAB).

COLOGANIA

Cologania broussonetii

ARGENTINA. Córdoba: (unclear text), Alta Gracia, III.1930, *D. O. King s.n.* (BAB).

CONDYLOSTYLIS CANDIDA

ARGENTINA. Corrientes: Carambola, 20.III.1987, *T. M. Pedersen 14847* (UPCB16087).

COURSETIA

Coursetia brachyrachis

BOLIVIA. Tarija: Gran Chaco, Boca Chica, Carapari , Serranía de Aguarague, 04.X.2007, *A. Llyully 1026* (MO).

Coursetia hasslerii

ARGENTINA. Salta: 1 km S de Guachipas, en dirección a El Cebilar, 16.III.2001, *R. H. Fortunato et al. 7179* (BAB). Santiago del Estero: Guasayán, 4 km al NNW de Santa Catalina por ruta nacional 64 en dirección a Lavalle, 31.XI.1995, *R. H. Fortunato et al. 5176* (BAB).

CROTALARIA

Crotalaria chaco-serranensis

ARGENTINA. Santiago Del Estero: Choya, camino de Frías a La Punta, 23.I.1986, *R. L. Pérez Moreau & C. L. Petetin 5367* (BAB).

Crotalaria incana

ARGENTINA. Formosa: Pilagás, 18 km al Este del Espinillo por ruta Nac. 86, 25º01'S 58º27'W, 25.XI.1999, *R. H. Fortunato & al. 6262* (BAB). Salta: Rosario de Lerma: Camino a corralito a ½ km del puente sobre el Río Rosario, 5.V.1995, *P. Hoc & A. Lamarque s.n.* (BAB 91922). Santiago del Estero: Copo, Mataque, 10.VII.1947, *F. E. Luna 246* (LIL).

Crotalaria micans

ARGENTINA. Santa Fe**:** Gral.Obligado, entre Ocampo y Pto. Ocampo, 10.II.1938, *A. Ragonese 3233* (BAB)

Crotalaria pilosa

ARGENTINA. Chaco: Primero de Mayo: Colonia Benítez, 21.I.1959, *A. G. Schulz 10319* (BAB)

Crotalaria stipularia

BOLIVIA. Chuquisaca: Iguembe, 17.X.2007, *M. Jiménez 501* (MO). ARGENTINA. Chaco: Colonia San Miguel, Ea. Varela ,12.I.1951, *A. G. Schulz 8019* (BAB).

CYCLOLOBIUM

Cyclolobium brasiliense

PARAGUAY. Paraguarí. Parque Nacional Ybycuí, límite N del Parque, camino a Ayo. Corrientes, 6-X-1984, Pérez 402 (CTES, PY, SI).

DALBERGIA

Dalbergia frutescens

PARAGUAY. Paraguarí: National Park Ybycuí, 31.X.1989, *E. M. Zardini & M. Velázquez 15635* (MO).

DALEA

Dalea elegans

Argentina. Córdoba: Colón, Saldán, V.1939, *R. Lahitte s.n.* (BAB 60286).

DESMODIUM

Desmodium affine

ARGENTINA. Chaco: Primero de Mayo, Colonia Benítez, 02.V.1994, *G. Seijo 1013* (CTES).

Desmodium barbatum

ARGENTINA. Corrientes: CApital, Santa Ana, 11.II.1976, *A. Schinini 12559* (CTES).

Desmodium cuneatum

ARGENTINA. Corrientes: Lavalle, Yataytí, calle por Ruta Provincial 120 em dirección a La Ruta nacional 12, 29°01’S 58°23’W, 31.I.2003, *R. H. Fortunato Et al. 7924* (BAB).

Desmodium distortum

ARGENTINA. Formosa: Pilcomayo, 45 km W de Clorinda sobre ruta a Laguna Blanca-Colonia Primavera, 12.VI.1984, *M. M. Arbo Et al. 2606* (CTES93693).

Desmodium glabrum

PARAGUAY. Boquerón: 29 km SE de Nueva Asunción, 13.V.1989, *A. Krapovickas et al. 45359* (BAB).

Desmodium hickenianum

ARGENTINA. Corrientes: Empedrado, Estación Experimental Del INTA, 07.XII.1978, *A. Schinini 12510* (CTES).

Desmodium incanum

ARGENTINA. Catamarca: Paclín, Balcosna, 1250 m s.m., 14.I.1928, *Venturi 7161* (SI). Formosa: Pirané, Palo Santo, Ruta 81, km 1317,2, 16.II.2012, *M. M. Cabrera 24* (CTES).

Desmodium intermedium

ARGENTINA. Formosa: Guaycolec, II-1919, *Joergensen 2721* (SI).

Desmodium neo-mexicanum

BOLIVIA. Chuquisaca: El Salvador-CIMBOC, El Panta, 04.V.1996, *C. Saravia Toledo 13879* (CTES 261527). ARGENTINA. Catamarca: El Alto,Albigasta, 21-IV-1983, *Renolfi 304* (BAB, CTES).

Desmodium pachyrizum

ARGENTINA. Formosa: El Colorado, Puerto Veloz, a 18 km de éste, 6.III.1979, *J. G. Fernández 554* (CTES 67691). Tucumán: Tafí Viejo, Los Chamicos, I-1924, *Venturi 2795* (BA, SI).

Desmodium polygaloides

ARGENTINA. Corrientes: El Sombrerito, Estación Experimental del INTA, 24.I.1974, *C. Quarín et al. 1975* (CTES 67673).

Desmodium tortuosum

ARGENTINA. Córdoba: Punilla, 1 km antes de Copina y primer ouente por ruta provincial 14, 31°33'S 64°42'W, 14.III.1996, *R. H. Fortunato et al. 5334* (BAB). Tucumán. Corrientes: Capital, orillas del río Paraná, Corrientes, (escapado de cultivo), 18-VI-1972, *Schinini et al. 4800* (CTES)

Desmodium venosum

ARGENTINA. Chaco: Primero de Mayo, Colonia Benítez, I-1934, *Schulz 1176* (CTES).

Desmodium uncinatum

ARGENTINA. Chaco: Primero de Myo, Colonia Benítez, camino viejo a C. Benítez, 1 km antes del puente, 19.IV.2001, *V. Solís Neffa 509* (CTES326953). Córdoba: Calamuchita, Altos Pampa, 1200 m s.m., 16.II.1953, *Krapovickas et al.* (CTES, LIL, SI)

DIOCLEA

Dioclea burkartii

ARGENTINA. Chaco: Bermejo, 11,2 km al N del ingreso a la ruta en dirección a la Isla del Cerrito, 01.II.2003, *R. H. Fortunato et al. 7950* (BAB, MO).

Dioclea violacea

PARAGUAY. Paraguarí: Macizo Acahay, 14.VII.1988, *E. M. Zardini 5760* (MO).

DISCOLOBIUM

Discolobium junceum

ARGENTINA. Formosa: Pilcomayo, Parque Nacional Pilcomayo, cerca de Pto. Santa María, 09.XI.1991, *R. H. Fortunato Et al. 2110* (BAB).

Discolobium psoraleaefolium

ARGENTINA. Formosa: Ruta 11, 30 Km al sur de Formosa, 19.IV.1987, *C. L. Cristóbal & A. Krapovickas 2167* (IAC, LIL);

Discolobium pulchellum

ARGENTINA. Chaco: Puerto Bermejo, 07 March 1901, *Kermes 662* (BAB).

DOLICHOPSIS

Dolichopsis paraguariensis

ARGENTINA. Chaco: Sargento Cabral, alrededores de Colonias Unidas, 25.XI.1990, *R. H. Fortunato Et al. 1687* (BAB). Formosa: Patiño, Soldado Sánchez, 09.XII.1981, *A. Cabral & A. M. Molina 779* (BAB).

ERIOSEMA

Eriosema tacuaremboense

ARGENTINA. Chaco: Colonia Benítez, I.1931, *Schultz 132* (BAB).

Eriosema platycarpon

PARAGUAY. Cordillera: Tobatí, Ybytú Silla Mesa, 25°12'S 57°07'W, 03.III.1991, *E. M. Zardini & U. Velázquez 26791* (BAB).

Eriosema simplicifolium

ARGENTINA. Chaco: *Krapovickas 32280* (CTES).

ERYTHRYNA

Erythrina christa-galli

PARAGUAY. Central: Estero del Ypoá, cerro Pé, 23.X.1992, *E. M. Zardini & P. Aquino 33270* (BAB).

Erythrina dominguezii

PARAGUAY. Ñeembucú: Abundante en bosques residuales, XI.1978, *L. Bernardi 18518* (G).

Eryhtrina falcata

Tucumán: Capital, río Salí, 27.X.1925, *S. Venturi 3531a* (BAB).

GALACTIA

Galactia benthamiana

PARAGUAY. Presidente Hayes: Between Puerto Galileo and Fortín Nowak, 10 km W of Puerto Galileo, 16.II.1994, *E. M. Zardini & B. Bonifacia Benítez 38400* (MO).

NOTE: This species was reported for Dry Chaco in Argentina, but we could not find material from this region.

Galactia glaucescens

BRASIL. Mato Grosso do Sul: Porto Murtinho: Beira de estrada, 25 Agosto 2004, fl., fr., *G.P. Nunes et al. 63* (CGMS)

Galactia glaucophylla

ARGENTINA. Córdoba: Punilla, ruta provincial 17, base del Cerro Uritorco, 20.I.2004, *R. Pozner & M. Belgrano 319* (BAB). Santiago del Estero: Quebrachos, Sumamp0061 Viejo, 27.X.1946, *A. Ragonese 6307* (BAB).

Galactia latisiliqua

Argentina. Chaco: General Güemes, alrededores de Wichi, camino a El Sauzal, 29.II.2000, *R. H. Fortunato et al. 6490* (BAB); Primero de Mayo, Colonia Benítez, 10.XI.1963, *A. G. Schulz 12615* (BAB). Córdoba: Punilla, Villa Carlos Paz, a 300 m del Puente Negro, 11.II.1996, *Lamarque s.n.* (BAB 91956).

Galactia longifolia

PARAGUAY. Presidente Hayes: Km. 192 de la ruta 9, 9.III.2001, *R. H. Fortunato et al. 8543* (BAB9.

Galactia marginalis

ARGENTINA. Formosa: Guaycolec, X.1918, *P. Jörgensen 3219* (SI). Tucumán: Cruz Alta, Campo Alegre, 29.X.1992, *S. Venturi 1918* (BAB).

Galactia paraguariensis

BRAZIL. Mato Grosso do Sul: Porto Murtinho, 16.II.2007, *F.M. Alves et al. 258* (CGMS)

Galactia striata* var. *striata

ARGENTINA. Corrientes: Capital, Molina Punta, 04.III.1998, *S. G. Tressens & A. Schinini 5969* (BAB). Jujuy: Palma Sola, 30.III.1983, *O. Ahumada 4644* (SI).

Galactia striata* var. *crassirachis

ARGENTINA. Tucumán: Graneros, 31.VIII.1968, *S. G. Tressens 68* (SI).

Galactia texana* var. *texana

Argentina. Salta: Rivadavia Banda Norte, 17 km al W de Hito 1 por ruta provincial 54 en dirección a Campo Durán, 04.III.2002, *R. H. Fortunato et al. 7577* (BAB). San Luis: Capital, 15 km de Villa de La Quebrada hacia el desvío a El Suyuque, 07.I.1980, *R. Rossow & C. Canale 31* (BAB).

Galactia texana *var. degasperii*

ARGENTINA. Formosa: Ing. Juárez, 14.I.1957, *A. Burkart 20288* (SI).

GEOFFROEA

Geoffroea decorticans

ARGENTINA. Chaco: Col. Resistencia (El Tragadero, camino a Col. Benítez), 09.IX.1966, *A. G. Schulz 15754* (BAB). Santiago Del Estero: Capital, 16.V.1938, *N. R. Ledesma s.n.* (BAB). Salta: La Viña, Coronel Moldes, costados de la Ruta Nacional 68, 5-6 km de Coronel Moldes, 26.XII.1998, *J. Tolaba 1304* (BAB).

Geoffroea spinosa

ARGENTINA. Chaco: Puerto Antequera, II.2001, *J. Greppi 37* (BAB). Salta: Rivadavia Banda Norte, camino a 100 m del desvío a San Matías, 1,5 km del desvío a Hito 1 en dirección E, 04.III.2002, *R. H. Fortunato et al. 7572* (BAB).

HELICOTROPIS

Helicotropis linearis

This species was cited in Maréchal et al. (1978) but we could not find material of reference from Gran Chaco ecoregion.

HOLOCALYX

Holocalyx balansae

ARGENTINA. Chaco: Colonia Benítez, 04.X.1942, *A. G. Schulz 3547* (BAB).

INDIGOFERA

Indigofera asperifolia

ARGENTINA. Chaco: Colonia Benítez, 11.X.1964, *A. G. Schultz 14123* (BAB). Tucumán: Cruz Alta, Las Cajas, 400 m, 30.XI.1924, *S. Venturi 1480a* (BAB).

Indigofera guaranitica

ARGENTINA. Chaco: Sargento Cabral, Parque Nacional Chaco, 23.XI.1991, *R. H. Fortunato Et al. 2590* (BAB).

Indigofera hirsuta

BRAZIL. Mato Grosso do Sul: Porto Murtinho, 05.IV.2001, *A.L.B. Sartoriet al.470* (CGMS).

Indigofera parodiana

ARGENTINA. Chaco: Bermejo, 8 km al E de Bosch, por ruta 81 camino a Las Lomitas, 10.XI.1983, *R. H. Fortunato Et al. 426* (BAB). Jujuy: Capital, camino al aeropuerto El Cadillal, Arroyo Palo Marcado, 23.I.1976, *A. L. Cabrera 27480* (BAB).

Indigofera spicata

BRAZIL. Mato Grosso do Sul: Porto Murtinho, Estrada para Fazenda Campo Florido, 15.XII.2011, *A.L.B. Sartori et al. 1109* (CGMS).

Indigofera microcarpa

PARAGUAY. Concepción: Itapucú Guazú, playas río Paraguay, I.1917, *Rojas 2885* (SI).

Indigofera sabulicola

ARGENTINA. Corrientes: San Cosme, Ensenada Grande, 06.X.1971, *A. Krapovickas Et al. 19993* (BAB).

Indigofera suffruticosa

BOLIVIA. Tarija: Ruta Tarija – Villamontes, Entre Ríos, 1330 m, 21.V.1971, *A. Krapovickas et al. 19085* (BAB). ARGENTINA. Corrientes: Itatí, 13.V.1945, *A. Soriano 1635* (BAB). Formosa: Ibarreta, 15.IV.1992, *A. Bordón s.n.* (BAB).

LATHYRUS

Lathyrus macrostachys

ARGENTINA. Chaco: Ruta 11, salida Del camino a Gob. Vedia, X.1970, *A. G. Schultz 17901* (CTES 124407). Formosa: Ruta 11, rio Salado, 4 km S de Tatané, 22.IX.1967, *O. Boelcke et al. 13325* (BAB).

Lathyrus nigrivalvis

ARGENTINA. Chaco: Sargento Cabral, Parque Nacional Chaco, alrededores del río Negro, 22.XI.1991, *R. H. Fortunato Et al. 2542* (BAB).

Lathyrus pusillus

ARGENTINA. Chaco: Colonia Benítez, X.1934, *A. G. Schulz 1144* (SI).

LONCHOCARPUS

Lonchocarpus nitidus

PARAGUAY. Cordillera: Road Emboscada-Nueva Colombia, 5 km SE of Emboscada, 04.VIII.1990, *E. M. Zardini & H. Velázquez 22623* (AS, MO).

LEPTOLOBIUM

Leptolobium elegans

PARAGUAY. Cordillera: Serranía de Tobatí, Meseta Ybytú Silla, 23.X.1988, *E. M. Zardini 7604* (MO).

LEPTOSPIRON

Leptospiron adenanthum

ARGENTINA. Chaco. Roca, 01.V.1996, *B. Pengelly et al. 131* (CTES 261925).

BOLIVIA. Tarija: Entre Narváez y Entre Ríos, 24.X.1980, *F. Zuloaga et al. 1303* (SI). PARAGUAY. Cordillera: Salto Pirharetá del río Piribebuy, 01.II.1980, *M. Vázquez Avila 184* (SI).

LUETZELBURGIA

Luetzelburgia sotoi

BOLIVIA. Santa Cruz: Chiquitos, entre Robore´ y El Carmen, aproximadamente 20 km, antesde llegar la comunidad de Candelaria, 18_23031.500S, 59_39039.300W, 8.Vi.2009 (fl, bud), D. Soto et al. 1131 (CTES

LUPINUS

Lupinus gibertianus

ARGENTINA. Corrientes: San Cosme, Paso de la Patria, 19.IX.1971, *S. G. Tressens et al. 150* (CTES).

Lupinus gibertianus* var. *berroanus

ARGENTINA. Chaco: Primero de Mayo, Colonia Benítez, sobre albardón de la laguna del río Tragadero, 30.IX.1971, A. Martínez et al. s.n. BAA 9504 (SI). Corrientes: Concepción, Carambola, 17.XII.1974, *A. Burkart et al. 30888* (SI).

Lupinus gibertianus* var. *gibertianus

ARGENTINA. Santa Fe: Capital, San José del Rincón, 02.XI.1939, *Ragonese 4117* (SI).

Lupinus gibertianus* var. *redbeckianus

ARGENTINA. Corrientes: Capital, Escuela de Agricultura, 06.X.1967, *M. M. Arbo* 68 (CTES). Tucumán: Leales, lecho Del río Salí, XI.1919, *S. Venturi 630* (SI).

MACHAERIUM

Machaerium aculeatum

PARAGUAY. Concepción: Around Horqueta, 25.VI.2002, *E. M. Zardini & R. Gómez 58732* (MO).

Machaerium acutifolium

BOLIVIA. Santa Cruz: Within the ""Flora de la Region del Parque Nacional Amboro"", but above the 700 m contour, no date, *M. H. Nee 38908* (MO).

Machaerium eriocarpum

BOLIVIA. Santa Cruz: *J. R. Wood 17654* (MO). PARAGUAY. Central: Trinidad, II.1944, *Pavetti 11051* (AS).

Machaerium paraguariense

PARAGUAY. San Pedro: Around San Pedro de Ycuamandiyú, 26.VI.2001, *E. M. Zardini & M. Vera 56869* (MO).

Machaerium pilosum

BOLIVIA. Santa Cruz: Cordillera, Charagua, 7 km NW del pueblo, orillas del Río Ovai, 12.IV.1996, *I. Vargas 446* (MO).

Machaerium scleroxylon

BOLIVIA. Chuquisaca: Luis Calvo, Parque Nacional y Area de Manejo Integrado "Serranía del Iñao", Comunidad Ticucha. Serranías intermedias de la Serranía del Iñao, 25.III.2003, *M. Serrano et al. 4140* (MO).

Machaerium stipitatum

PARAGUAY. Cordillera: Atyra, 6 – 7 km de Altos, no date, *L. Pérez de Molas 981* (MO).

MACROPTILIUM

Macroptilium atropurureus

ARGENTINA. Salta: Rivadavia, ruta 81, 3 km W de Los Blancos, 08.V.1998, *G. Seijo & A. Krapovickas 1957* (BAB).

Macroptilium bracteatum

PARAGUAY. Presidente Hayes: Puerto Militar, puente sobre el río Paraguay, frente a Concepción, 10.XII.1989, *R. Vanni et al. 1275* (CTES 177127).

Macroptilium erythroloma

ARGENTINA. Formosa: Pirané, Estación Experimental Agropecuaria El Colorado, 03.III.1963, *Bordón s.n.* (CTES 408767).

Macroptilium fraternum

ARGENTINA. Salta: 16 km E de Salta, 01.V.1996, *B. Pengelly et al. 62* (CTES261917).

Macroptilium geophyllum

ARGENTINA. Salta: Rivadavia, 18 km al E de Dragones, 12.II.1992, *C. Saravia Toledo & R. Neumann 10782* (BAB).

Macroptilium lathyroides

PARAGUAY. Alto Paraguay: Puerto Diana a 5 km de Bahia Negra, I.1976, Arenas 1369 (SLBACP); Central: San Lorenzo, ciudad universitaria, 24.X.l974, Arenas 939 (SI). ARGENTINA. Formosa: 55 km

O de Formosa, borde ruta 81, 5.XII.1972, *V. Maruñak et al. 410* (CTES 136887).Salta: Rivadavia, Los Blancos, cañada a 3 km al W del pueblo, 26.I.1983, *A. Maranta & P. Arenas 201* (BAB).

Macroptilium longipedunculatum

PARAGUAY. Central: Esteros del Yopá, 18 km S of Puerto Guyratí, 04.III.1993, *E. M. Zardini & T. Tillería 35400* (BAB).

Macroptilium panduratum

BOLIVIA. Santa Cruz: Cordillera, Paja Colorada (28 km S de Camiri, camino a Boyuibe), 14.IV.1977, *A. Krapovickas & A. Schinini 31334* (CTES 112581).

PARAGUAY. Central: San Lorenzo, Capilla del Monte, 28.II.1985, Bordas 3648 (CTES).ARGENTINA. Salta: Anta, Pozo Largo, a 18 km SSE de Joaquín V. González, 21.V.1985, *C. Saravia Toledo 1147* (CTES 123580).

Macroptilium prostratum

ARGENTINA. Corrientes: Saladas, Estancia La Clívia, 20.III.1968, *R. Carnevali 1061* (CTES123617).

Macroptilium psammodes

ARGENTINA. Corrientes: Esquina, río Guayquiraró, Paso Yunque, 13.III.1975, *A. Krapovickas et al. 27617* (CTES 137012).

MEDICAGO

Medicago lupulina

ARGENTINA. Tucumán: Tapia, 15.XII.1911, *Rodríguez 183* (SI). Mentioned for Humid and Dry Chaco areas but we could not obtain reference specimens.

Medicago polymorpha

ARGENTINA. Catamarca: Capital, 05.IX.1909, *no collector 22* (SI).

Medicago sativa

ARGENTINA. Córdoba: Valle los Reartes, no date, *A. Castellanos 416* (SI).

MELILOTUS

Melilotus albus

ARGENTINA. Córdoba: Entre San Pedro y El Chico, camino a Chancaní, 04.I.1956, *A. T. Hunziker 502* (SI). Santiago Del Estero: Ojo de Agua, ruta 9, alrededores de Arroyo Lito, 21.IX.1989, *R. L. Pérez Moreau et al. 4598* (BAB).

NOTE: This species was mentioned for Humid Chaco areas but we could not obtain reference specimens. Since the extensive distribution of this naturalized species overall Argentina, we considered it as occurring in all subregions from Gran Chaco.

Melilotus indicus

ARGENTINA. Córdoba: Sierra Chica, Estancia La Reducción, a 700 m, 27.XII.1935, *A. Burkart 7338* (SI). Santa Fe: Zona río Salado, ruta Las Colonias, 24.XI.1957, *Martínez Achenbach 342* (SI).

MUELLERA

Muellera fluvialis

ARGENTINA. Formosa: Estancia Guaycolec, 25 km N de Formosa, Ruta Nacional n°11, 20.X.1989, *G. Placci & S. Arditi 138* (CTES 157071).ARGENTINA. Formosa: Estancia Guaycolec, 25 km N de Formosa, ruta nacional 11, 20.X.1989, *G. Placci & S. Arditi 138* (BAB).

Muellera nudiflorens

BRAZIL. Mato Grosso do Sul: Porto Murtinho, Fazenda Santa Vergínia, caminho para Fazenda Toro Pampa, 22Outubro2013, fl., *T.R.F. Sinaniet al.160* (CGMS). BOLIVIA. Santa Cruz: Cordillera, Parque Nacional Kaa-Iya Del Gran Chaco, 58 km O del campamento San José, sobre el gasoducto, 13.VI.1998, *A. Fuentes & G. Navarro 2425* (CTES 392338).

Muellera sericea

ARGENTINA. Chaco: Primero de Mayo, Colonia Benítez, ribera del río Tragadero, 13.XI.1968, *A. G. Schultz 16487* (BAB).

MYROCARPUS

Myrocarpus frondosus

PARAGUAY. Central: Jardín Botánico y Zoológico, Trinidad, Asunción, VIII.1991, *B. Pérez 971* (BAB).

NEONOTONIA

Neonotonia wightii

ARGENTINA. Formosa: Pilcomayo, estancia Bouvier, camino viejo, alrededores Del puente sobre el riacho Negro, 30.III.1993, *R. H. Fortunato ET AL. 4174* (BAB).

NISSOLIA

Nissolia fruticosa

ARGENTINA. Formosa: Ruta 11 antigua, Dalmacia–Riacho Monte Lindo Grande (inmediaciones), 24.I.1981, *C. A. Petetín & A. M. Molina 1483* (BAB). Salta: Cerro San Bernardo, 08.III.1905, *C. Spegazzini s.n.* (BAB 14420).

OTHOLOBIUM

Otholobium higuerilla

ARGENTINA. Catamarca: Las Juntas, 15.IV.1910, *P. L. Spegazzini s.n.* (BAB 33408).

PHASEOLUS

Phaseolus vulgaris* subsp. *aborigeneous

ARGENTINA. Salta: Rosario de Lerma, Quebrada del Toro, 8 km al N de Campo Quijano desde el ingreso, 06.III.2001, *R. H. Fortunato et al. 6939* (BAB).

Phaseolus lunatus* var. *silvester

ARGENTINA. Chaco: Col. Benítez. Cultivado, procede de Chaco, pte. río de Oro, ruta 90, entre El Zapallar Y El Colorado, 02.IV.1974, *A. G. Schulz 18760* (MO).

POIRETIA

Poiretia tetraphylla

ARGENTINA. Chaco: Las Palmas, no comprehensive date, *P. Jörgensen 2138* (SI). Santa Fe: Mocoví, no date, *S. Venturi 52* (SI). Formosa: Formosa apparently, 06.XII.1900, *Kermes 448* (BAB 1284bis). Santiago del Estero: J. F. Ibarra, laguna Saladas, camino entre ruta provincial 56 y Los Juríes, 10.III.1986, *A. M. Molina & J. Hilfer 3290* (BAB).

POISSONIA

Poissonia hypoleuca

ARGENTINA. Salta: Moldes, El Dique, 14.XI.1947, *Dawson 1995* (BAB 71315).

PTEROCARPUS

Pterocarpus santalinoides

ARGENTINA. Chaco: Boca Río de Oro, destacamento de gendarmería, 17.XII.1950, *A. G. Schulz 939* (BAB).

RHYNCHOSIA

Rhynchosia balansae* var. *balansae

PARAGUAY. Boquerón: 18,2 km al E de Loma Plata, por ruta de acceso em dirección a ruta 9, 10.III.2005, *R. H. Fortunato et al. 8608* (BAB). Cordillera: Cerro Tobatí, 11.XII.1987, *E. M. Zardini 3982* (BAB).

Rhynchosia balansae* var. *psilantha

ARGENTINA. Corrientes: Concepción, 28 km SE de Concepción, Ea. El Tránsito, 20.V.1976, *A. Schinini et al. 13037* (SI).

Rhynchosia burkartii

ARGENTINA. Formosa: Patiño, 3 km de Sargento Leyes por ruta 86 en dirección a San Martín 2, 24.III.1992, *R. H. Fortunato Et al. 3090* (BAB); 8 km al N de Las Lomitas por ruta 28, 10.XI.1994, *R. H. Fortunato 4392* (BAB).

Rhynchosia corylifolia

ARGENTINA. Chaco: Colonia Benítez, 28.X.1960, *A. G. Schulz 11384* (BAB).

Rhynchosia diversifolia* var. *diversifolia

Argentina. Formosa: Pilcomayo, Parque Nacional Pilcomayo, alrededores de Paratodo, camino a Ea. La Angela, 17.III.1992, *R. H. Fortunato et al. 2765* (BAB). Tucumán: Tucumán y alrededores, verano 1907 – 1908, *Dinelli 709* (BAB 26108).

Rhynchosia diversifolia* var. *prostrata

ARGENTINA. Santa Fe: Garay, Colonia Mascías, 24 – 30.XI.1942, *R. A. Spegazzini 38* (BAB 64527).

Rhynchosia edulis

ARGENTINA. Chaco: Sargento Cabral, 4 km al O de Colonia Benítez hacia Salto de La Vieja, 15.XI.1983, *R. H. Fortunato et al. 703* (BAB). Córdoba: La Balsa, rio Anisacate, 12.XII.1963, *Ragonese & Piccinini 9465* (BAB 78207). Formosa: Patiño, Ibarreta, 15.IV.1992, *A. O. Bordón 113* (BAB).

Rhynchosia minima

ARGENTINA. Chaco: Bermejo, Isla del Cerrito, costa cerca del ingreso a la isla, 06.III.2005, *R. H. Fortunato et al. 8502* (BAB).

Rynchosia naineckensis

BOLIVIA. Tarija: Gran Chaco, 15 km hacia el norte de Yacuiba, 630 m, 23.IX.1985, *S. Beck et al. 11470* (BAB). ARGENTINA. Formosa: Pilcomayo, Laguna Naineck, km 1334, 12.XII.1981, *A. M. Molina et al. 976* (BAB).

Rhynchosia senna* var. *senna

ARGENTINA. Chaco: San Fernando, A 9 km de Basail hacia el N por RN 11, 12.XI.1990, *R. H. Fortunato et al. 1226* (BAB). Jujuy: San Pedro, Ruta Nacional 34, entre El Arrayanal y El Quemado, 10.X.2002, *F. Zuloaga et al. 7456* (SI, MO). Santiago del Estero: Copo, ruta nacional 16, 7 km al SE de El Caburé, 07.III.1986, *A. M. Molina & J. Hilfer 3070* (BAB).

Rhynchosia senna* var. *texana

ARGENTINA. Santiago del Estero: Guasayán, 43 km al S de San Pedro por ruta nacional 157, 31.XI.1995, *R. H. Fortunato & R. Micheli 5182* (BAB).

SESBANIA

Sesbania exasperata

ARGENTINA. Formosa: Parque Nacional Pilcomayo, alrededores del Puesto de Gendarmería La Ángela, 18.III.1992, *R. H. Fortunato et al. 2814* (BAB); Matacos, Ing. Juárez, Barrio Obrero, 25.II.1983, *P. Arenas 2314* (BAB).

Sesbania virgata

ARGENTINA. Chaco: Bermejo, Las Palmas, en monte cerca del puerto, 14.XI.1983, *R. H. Fortunato et al. 570* (BAB). Santiago del Estero: Capital, lecho del río Dulce, 21.V.1949, *A. Soriano & W. Barrett 3552* (BAB).

STYLOSANTHES

Stylosanthes guianensis

PARAGUAY. Paraguarí: Caapucú, 08.III.1979, *J. G. Fernández 560* (CTES 107860). ARGENTINA. Córdoba: Santa María, 07.I.1958, *Ariza Espinar 951* (CORD).

Stylosanthes hamata

BRAZIL. Mato Grosso do Sul: Porto Murtinho, 5.IV.2001, *A.L.B. Sartoriet al.471* (CGMS).

Stylosanthes leiocarpa

ARGENTINA. Corrientes: Mburucuyá, 25 km de Mburucuyá, 26.XII.1976, *A. Krapovickas 29953* (CTES25066).

Stylosanthes macrosoma

PARAGUAY. Alto Paraguay: Parque Nacional Defensores Del Chaco, Cerro León, 23.XI.1984, *R. Duré 464* (MO). ARGENTINA. Corrientes: Capital, camino a Santa Ana, Laguna Soto, 01.XI.1975, *A. Schinini 12124* (CTES 19807).

NOTE: The presence of *S. macrosoma* in Sierra Chaco would be doubtful, since it was reported Dr. Burkart (1939) but Vanni (2017) expressed dudes because of the absence of fruits in the known specimens from this region.

Stylosanthes maracajuensis

BRAZIL. Mato Grosso do Sul: Porto Murtinho, Beira de estrada, 13.I.2005, *L.E.A.M. Lescanoet al.102* (CGMS).

Stylosanthes montevidensis

ARGENTINA. Corrientes: Esquina, 30 km de Esquina, Estancia Santa Bárbara, 12.II.1974, *R. Carnevali 3390* (CTES 19722). Santiago Del Estero: Belgrano, camino interno Bandera – Guardia Escolta, 10 km, 25.II.1973, *M. Elisetch 321* (BAB).

Stylosanthes recta

PARAGUAY. Parque Valle Natural, 25 km S de Filadelfia, 7.XII.1992, *Krapovickas & C. L. Cristóbal 44219* (CTES, G, SI).

Stylosanthes scabra

BOLIVIA. Tarija: PARAGUAY. Boquerón: Filadelfia, Isla Poí, 27.I.2006, *Glatzle s.n.* (CTES). ARGENTINA: Salta: Capital, lomadas 1 ayo. ruta Salta a San Lorenzo, 21 Mar. 1979, J. Fernández s.n. (CTES).

Stylosanthes viscosa

PARAGUAY. Cordillera: Emboscada, Segunda Compañía, 23.II.1987, *E. Bordas & G. Sehmeda 4256* (CTES115837).

SWEETIA

Sweetia fruticosa

PARAGUAY. Central: Asunción, X.1929, *T. Rojas 8659* (MO).

TEPHROSIA

Tephrosia adunca

PARAGUAY. Boquerón: Proposed National Park Médanos Del Chaco, 13.XII.1998, *E. M. Zardini & N. Duarte 47912* (BAB). ARGENTINA. Corrientes: Esquina, 26.III.1953, *A. Mutinelli s.n.* (BAB 61870).

Tephrosia cinerea

PARAGUAY. Boquerón: Proposed National Park Medanos del Chaco, 12.XII.1998, *E. M. Zardini & N. Duarte 49673* (BAB).

Tephrosia hassleri

PARAGUAY. Boquerón: Proposed National Park Médanos Del Chaco, 12.XII.1998, *E. M. Zardini & N. Duarte 49673* (MO).

TRIFOLIUM

Trifolium polymorphum

ARGENTINA. Corrientes: Mburucuyá, Estancia Santa Teresa, 12.XII.1954, *A. Burklart 19490* (SI).

Trifolium pratense

ARGENTINA. Chaco: Primero de Mayo, Colonia Benítex, 06.XI.1961, cultivo experimental, *A. G. Schultz 11650* (BAB).

Trifolium repens

ARGENTINA. Tucumán: Capital, rio Salí, 15.IX.1970, *S. Venturi 907* (SI).

VICIA

Vicia epetiolaris* var. *epetiolaris

ARGENTINA. Formosa: Ruta 11 y arroyo Salado, 02.IX.1971, *A. Krapovickas et al. 19625* (CTES, SI).

Vicia epetiolaris* var. *microcarpa

ARGENTINA. Chaco: Bermejo, Campo Veláz, 10.XI.1966, *A. G. Schultz 15674* (CTES).

Vicia graminea* var. *graminea

ARGENTINA. Chaco: Isla Antequera, 12.XI.1958, *A. G. Schulz 10229* (SI).

Vicia graminea* var. *transiens

ARGENTINA. Formosa: Pilcomayo, Ruta Nacional 86, 3 km al E de Palma Sola, 19.X.1975, *B. G. Piccinini & A. Leguizamón 2676* (BAB).

Vicia macrograminea

ARGENTINA. Formosa: El Colorado, no date, *A. Bordón s.n.* (BAB).

Vicia pampicola* var. *pampicola

ARGENTINA. Chaco: Libertador General San Martín, 14 km al N de La Eduviges por ruta 90, 05.XI.1983, *R. H. Fortunato et al. 215* (BAB).

Vicia setifolia* var. *setifolia

ARGENTINA. Córdoba: Colón, Ea Ayala, 1100 m s.m., 30-XI-1976, *Alessandria 6948* (CTES). Formosa: Laishi, Colonia Presidente Irigoyen, 14.XII.1981, *A. Cabral & A. M. Molina 1024* (BAB).

VIGNA

Vigna longifolia

ARGENTINA. Corrientes: San Roque, Santo Domingo, próximo al río Santa Lucía, 10.IV.1970, *R. Carnevali 2215* (CTES111466).

Vigna luteola

ARGENTINA. Corrientes: Esquina, 26 km SE de Libertador, Estancia La Blanca, 12.III.1973, *A. Krapovickas et al. 27506* (108481). Santiago del Estero: Puente sobre el río Salado sobre ruta provincial ex92, 6,5 km al SW de Añatuya, 26.II.2004, .*R. Pozner & M. J. Belgrano 361* (CTES, SI).

ZORNIA

Zornia crinita

BOLIVIA. Chuquisaca: Luis Calvo: Entrada al Valle Itargua, 11.IV.1993, *C. Saravia Toledo et al. 11680* (MO). PARAGUAY. Boquerón: Estación Experimental Isla Poí, 30 km SE de Loma Plata, 25.II.1991, R. Vanni et al. 2242 (CTES 172291).

Zornia cryptantha

ARGENTINA. Corrientes: Mburucuyá, Estancia Santa Teresa, 07.I.1955, *T. M. Pedersen 3112* (MO).

Zornia diphylla

ARGENTINA. Corrientes: Capital, Perichón, 18.XII.1975, *A. Schinini & R. Martínez Crovetto 12254* (CTES 47484).

Zornia gracilis

ARGENTINA. Corrientes: Capital, Arroyo Riachuelo y Ruta 12, 05.XII.1976, *C. Quarín 3506* (CTES 47464).

Zornia latifolia

PARAGUAY. Paraguarí: Frente a Villa Florida, arroyo Tebicuary, 16.VI.1977, *A. Kravpickas & A. Schinini 32503* (CTES 47666). ARGENTINA. Córdoba: Sierra Chica, Estancia La Reducción, 05.I.1936, *A. Burkart 7381* (SI).

Zornia multinervosa

ARGENTINA. Chaco: Bermejo, Las Palmas, 14.XI.1983, *R. H. Fortunato et al. 613* (BAB).

Zornia pardina

ARGENTINA. Formosa: Laishi, Reserva Ecológica El Bagual, 15.I.2000, *A. Di Giacomo 457* (CTES320445). BOLIVIA. Santa Cruz: Cordillera, 6 km S de Lagunillas, 16.IV.1977, *A. CV. Krapovickas & A. Schinini 31434* (MO).

Zornia reticulata

PARAGUAY. Cordillera: Itacurubí, X.1970, *A. Schinini 3179* (CTES318147).

Zornia trachycarpa

ARGENTINA. Córdoba: Calamuchita, 1450 m s.m., Cumbrecita, 17-11-1953, Krapovickas 7598 (CTES). Corrientes: Empedrado, 16.I.1973, *R. Carnevali 3384* (CTES). Formosa: Patiño, 15 km NNW de Las Lomitas, 26.IX.1984, *A. Krapovickas & C. L. Cristóbal 46417* (BAB). Santiago del Estero: 28 de Marzo, Añatuya, 19.II.1947, *C. L. Schulz 1341* (LIL).

2. Key species per lineage

CAMPOS

LUPINUS GIBERTIANUS

PARAGUAY. Itapúa: Encarnación, IX.1915, *Hassler 1470* (G). Ñeembucú: Humaitá, 09.XI.1978, *T. Rojas 1470* (G). URUGUAY: Colonia: Artilleros, 17.XII.1943, *Bartlett 21221* (US); Cerro Carmelo, 06.XII.1934, *A. L. Cabrera 3198* (LP); Colonia, 11.XI.1919, *Castellanos s.n.*(BA56695); Riachuelo, X.1949, *Fabris 37* (LP). Montevideo: Montevideo, 20.VII.1866, *Gibert 467* (K). Salto: Salto, 04.XI.1902, *Berro 1775* (K). ARGENTINA. Chaco: Bermejo: Las Palmas, X.1917, *Jörgensen 2139* (SI) – Capital: Resistencia, campo alto, 10.X.1948, *Vega 885* (CTES) – Primero de Mayo: Colonia Benítez, no date, *Bacigalupo s.n.*(BAA9504); Margarita Belén, 07.XI.1947, *Aguilar s.n.*(LIL). Corrientes: Bella Vista: Bella Vista, 05.X.1976, *Fernández Velazco s.n.*(BA68959) – Capital: Camino a San Ana, 29.XI.1993, *A. M. Planchuelo 609* (ACOR; Parada Medina, 28.IX.1975, *Schinini & Martínez Crovetto 12213* (G); Perichón, 29.IX.1974,

A. Krapovickas 1248 (CTES) – Concepción: Carambola, 17.XII.1974, *Burkart 30888* (US) – Empedrado: Empedrado, 26.IX.1971, *A. Krapovickas 19958* (CTES) – Mburucuyá: Estancia Santa Tecla, no date, *Schwarz 8048* (LIL); Santa Teresa, 04.IX.1946, *T. M. Pedersen 1* (CTES) – General Paz: Puissoye, 26.X.1945, *T. Ibarrola 3618* (LIL) – Itatí: Itatí, 08.X.1964, *T. M. Pedersen 7073* (LP) – Ituzaingó: Ituzaingó, 30.IX.1993, *A. M. Planchuelo 618A,B* (ACOR) – Paso de los Libres: Río Miriñay, 04.XI.1973, *A. G. Schulz 18646* (CTES) – Saladas: Laguna Soto Saladas, 26.IX.1944, *Schwarz 59* (LIL) – San Cosme: Paso de la Patria, 03.X.1945, *Würth 158* (LIL); same locality, 21.II.1984, *A. Schinini & Martínez Crovetto 22785* (LIL) – San Luis del Palmar: Ruta 5 y Riachuelo, 05.X.1975, *Cristóbal 1331* (G) – San Martín: Tres Cerros, 16.IX.1979, *A. Schinini 18582* (CTES) – San Roque: Ea. Caaguazú, road to Tacuaritas, 25.X.1996, *Arbo et al. 6855* (LIL) – Santo Tomé: Estancia San Francisco, 05.XII.1970, *A. Krapovickas et al. 17195* (BAA, CTES); Garruchos, 20.IX.1974, *A. Krapovickas 25780* (LIL). Entre Ríos: Concordia: Concordia, IX.1917, *Alazraqui s.n.*(BA63399); INTA Concordia, 04.10.1993, *Planchuelo 658* (ACOR) – Gualeguaychú: Paranacito, XI.1917, *Hauman s.n.*(BA) – Islas del Ybicuy: Brazo Largo, 23.X.1980, *Troncoso & Bacigalupo 2880* (US); Médanos, no date, *Parodi 9462* (BAA). Misiones: Apóstoles: 10 km de Azara, 31.VIII.1974, *M. M. Arbo 2300* (CTES) – Candelaria: Mártires, 12.XII.1947, *Bertoni 3408* (LIL) – Capital: Posadas, 02.X.1911, *Muniez 6* (BAF) – Eldorado: Eldorado, 27.X.1949, *Schwindt 2151* (LIL); Puerto Victoria, 12.XI.1949, *Schwindt 2831* (CTES) – San Ignacio: San Ignacio, no date, *Hassler 444* (G); Santo Pipó, 03.X.1947, *Schwarz 4874* (CTES) – San Martín: Capiovy, 18.X.1948, *Schwindt 771* (CTES); Puerto Mineral, 24.VIII.1950, *Schwarz 10685* (LIL). Santa Fe: Capital: Acceso al túnel subfluvial Hernandarias, 03.XI.1995, *A. M. Planchuelo 812* (ACOR); Colastiné, no date, *Álvarez 924* (LIL); San José del Rincón, 05.X.1911, *Álvarez 868* (LIL); La Guardia, 19.XI.1946, *Huidobro 3069* (LIL) – Castellanos: Rafaela, 15.XI.1946, *Huidobro 3423* (CTES); Las Colonias: Esperanza, 15.XI.1946, *Huidobro 3262* (LIL) – General Obligado: Guadalupe, 21.XI.1946, *Huidobro 3411* (LIL) – San Jerónimo: Isla Campo Rico, 22.X.1977, *Franceschi 59* (SI) – Garay: Cayastá, 18.X.1980, *Perrone s.n.*(BA); same locality, 03.XI.1995, *A. M. Planchuelo 816* (ACOR). Tucumán: Simoca: Chicligasta, 14.X.1913, *Monetti 1245* (LIL).

MACROPTILIUM PSAMMODES

BRAZIL. Rio Grande do Sul: Atlântida: Entre Noiva do Mar e Atlântida, no date, Phadenhauer 209 (ICN) – Tramandaí: Playa de Tramandaí, 19.XII.1940, *I. Augusto s.n.*(ICN) – Encruzilhada do Sul: Passo da Guarda; Estrada (Uruguaiana) Harmonia-Livramento, km 34, 24.XI.1972, *D. Lima et al. s.n.*(ICN) – Guaiba: Fazenda São Maximiliano, 07.IV.1976, *S. T. Miotto s.n.*(ICN) – Itaquí: 42 km após Uruguaiana, para Itaquí, 19.XII.1972, *Pott et al. s.n.*(ICN); Fazenda Três de Outubro, 12.V.1985, *S. T. Miotto 1043* (ICN); Fazenda Três Figueiras, 05.IV.1977, *S. T. Miotto 421* (ICN) – São Borja: 1º banhado a 3 km de São Borja, 05.XII.1973, *B. E. Irgang et al. s.n.*(ICN); Banhado Grande, no date, *F. Cortés s.n.*(ICN) – Capão da Canoa, 20.II.1974, *N. I. Matzenbacher s.n.*(ICN) – Rio Grande: Taim, Capão a 23 km sul da sede, 05.XII.1983, *Almeida Rego et al. S.n.*(ICN) – Rosário do Sul: 30 km após Cacequí - via Rosário, 11.XII.1976, *M. Fleig 295* (ICN) – Santa Vitória do Palmar: 15 km Norte do Chuí, Hermenegildo, no date, *Phadenhauer 673* (ICN) –Santana do Livramento: Livramento-Quarai. BR 293, km 28, 07.I.1991, *Longhi-Wagner et al. 2396* (ICN) –Santiago: BR 453, km 158, 04.IV.2013, *J. F. M. Valls et al. 4616* (ICN) – São Francisco de Assís: Estrada de Santiago para São Francisco de Assis (25 km após Santiago), 10.XII.1976, *S. T. Miotto 323* (ICN); São Francisco de Assis para Alegrete, 14.XI.1975, *M. L. Porto et al. 1805* (ICN) – São Leopoldo, 06.XII.1934, *J. Dutra 787* (ICN) – Torres: Itapeva, 28.X.1985, *J. R. Stehmann 760* (ICN); pastizal en la cumbre de la Torre do Meio, alrededores de la lagoinha dos suspiros, 02.III.1990, *R. H. Fortunato et al. 1194* (BAB); Torres, 30.X.1976, *M. L. Porto 2226* (ICN) – Uruguaiana: BR 290 km 606-a 30 km de Uruguaiana, 03.IV.1977, *S. T. Miotto 402* (ICN); Uruguaiana, Entrada da cidade, no date, *I. Boldrini s.n.*(ICN) – Xangri-lá: Praia Rainha do Mar, no date, *Normann 931* (ICN). PARAGUAY. Cordillera: Valenzuela, 20.XII.1950, Vervoorst 1151 and 1169 (LIL); Lago Ypacaraí, 6.XII.1950, *Vervoorst 867* (LIL); Tobatí, 13.XII.1983, *Vanni 193* (CTES); ltacurubí, 26.IX.1967, Krapovickas 13296 (CTES); Eusebio Ayala, 26.IX.1967, *Krapovickas 13659* (CTES). Central: Ayo. Mboy, l/JAN/l973, Schininí 5699 (CTES); ltá, granja lsapoy, orilla Ayo. Lazarillo, 30.I.1966, Krapovickas, Cristobal y Palacios 12226 (SI). Paraguarí: 6.IX.1950, Burkart 18221 (SI); 26.XI. 1950, Sparre 589 (LIL); 6.IX.1885, Kurtz 302 (CORD); Villa Florida, 4.II.1966, Krapovickas et al. 12372 (SI); Cerro Sto Tomás, 29.XI.1950, Vervoorst 752 (LIL); no date, Hassler 1009(G); Barrerito, Caapacú, 20.XI.1951, Ramírez 1236 (SI); Carapeguá, In campo Duarte, no date, Hassler 1239 (G); Ybytimí a La Colmena, XI.1970, *A. Schinini 3481* (SI); de Ypacaray a Pirayrí, XI.1971, A. Schinini 3847 (CTES); Quindy, III.1969 and III.1971, *A. Schinini 2808 and 3826* (CTES). Guairá:

Independencia, 13.XI.1945, Rojas 13001(LIL); Villarrica, XI.1941, Rojas 9272 (SI). Caaguazú: Ruta 2 Km 592, entre Eusebio Ayala y Coronel Oviedo, 26.IX.1967, Boelcke 13402 (BAA). Itapúa: Encarnación, 19.XI.1911, Schoek 113 (LIL); .IX.1915, Hassler 1379 (SI); Trinidad, .XI.1917, Rojas 3139 (LIL, SI). Misiones: Santiago, Estancia La Soledad, 7.II. 1965, Pedersen 7677 (SI); Sapucay,XI.1970, *A. Schinini 3480* (SI); .VIII.1891, *Gotzche 497* (CORD); "Valle entre Cerro Patony y Cerro Negro", 5.IX.1885, *Kurtz 276* (CORD). URUGUAY. Rivera: Estación Tranqueras, no date, *Legrand 2414* (SI). Rocha: Cabo Polonio, 20.II. 1960, *Praderí 685* (LIL); 24.II. 1935, *l-losseus l 16* (CORD); La Paloma, 10.II.1948, *Castellanos 18570* (LIL). Canelones: Atlántida, S.II.1939, *Rosengurt 2822* (LIL); Balneario Parque del Plata, Ayo. Solís Chico, 4.II.1948, *A. Burkart 17527* (SI). Montevideo: Carrasco,.IV.1926, *Herter 967* (BAB,LIL,SI); 04.IV.1949, *Rosengurt 5289* (LIL); Cerro de Montevideo, no date, *Feliponei 4681* (SI). ARGENTINA. Corrientes: Ituzaingó: 40 km N de Ituzaingó, 23.X.1974, *Tressens 449* (CTES); Ruta Nac. 41, 40 km NE de Galarza, 08.XII.1974, *A. Schinini 2727* (CTES);Ruta 12, a 10 km S del Ayo. Itaembé, 25.I.1976, *Romanzuk 370* (SI) – Mercedes: A 3 km Empalme R14 con la R119, 05.XI.1973, *Sánchez 1052* (MACN); EEA INTA,09.XII.1965, *O. Royo 132* (CTES);Estancia Yragueri, 14.XI.1986, *Fernández s.n.*(BAFC); Ruta 7, km 798, 07.I.1947, *Huidobro 4099* (CTES) – Monte Caseros: Mocoretá, 25.II.1984, *Tressen*s 2510 (CTES) – Paso de los Libres: Paso de los Libres, XII.1956, *M. Crovetto 8472* (BAB) – San Miguel: Estancia Santa Ana Ñú; 12 km NE de San Miguel, 27.IV.1975, *Schinini 11442* (CTES) – Santo Tomé: 100 km E de Virasoro, río Aguapey, 2 km W de dicho rio, 23.V.1981, *Hoc et al. 1090* (BAFC); Arroyo Garabí, 22.III.1982, *Hoc et al. 1071* (BAFC); Establecimiento Las Marías, 22.III.1982, *Hoc et al. 1083* (BAFC); Estancia Timbaúva, 21.I.1976, *Romanzuck 184* (BAB); Gob. Virasoro, 23.I.1987, *Fernández 1018* (BAFC); Santo Tomé, XII.1949, *Crovetto 6315* (BAB). Misiones: Apóstoles: Apóstoles, 26.I.1926, *Clos 1913* (BAB) – Candelaria: Bonpland, 19.IX.1909, *Jorgensen 198* (BAB); Loreto, 14.III.1931, *Grüner 711* (BAB); Profundidad, 29.IX.1972,*A. Schinini 5409* (CTES); Santa Ana, 28.XI.1909, *Rodríguez 89* (BAB,MACN,SI)– Capital: 14 km al N de San José, 23.II.1989, *Palacios and Bravo 1282* (BAFC,CTES); Garupá, 07.X.1945, *Bertoni 2165* (CTES); Posadas I.1907, *Spegazzini 10513 (LP) and 20534 (BAB)*; Ruta 12 y Arroyo Itaembé, 17.I.1976, *A. Krapovickas & Cristóbal 12113 and 12116* (SI). – San Ignacio, no date, *Quiroga 1913* (MACN).

MIMOSA OLIGOPHYLLA

BRAZIL. Paraná:Ipiranga, Rodovia BR-277, 03.XII.1969, *G. Hatschbach 23064* (SI). Rio Grande do Sul: São Borja, Arroyo Ivaí, 15.XI.2009, *E. Barbosa et al. 2589* (BAB, MBM355626) – São Luis Gonzaga, São Luiz Gonzaga, margem da estrada, 17.XII.2008, J. *R. V. Iganci et al. 583* (ICN172206). PARAGUAY. Caazapá: Tapytá, III.1931, *P. Jorgënsen 4628* (LP13113). Cordillera: Piribebuy, III.1991, *F. Mereles 3981* (SI). Guairá: Azucarera de Tebicuary, río Tebicuary, 12.I.1973, *A. Schinini 5887* (SI). Paraguarí:Parque Nacional Ybycu'í, campo cerrado, 3 km N of Salto Cristal, 27.I.1989, *E. M. Zardini & forest ranger 10206* (MO4275689). ARGENTINA. Corrientes.Mburucuyá: Parque Nacional Mburucuyá, 28.XII.2004, *M. S. Ferrucci et al.* 2198 (BAB) – San Martín*:* Colonia Pellegrini, XII.1926, *R. A. Spegazzini 10015* (BAB) – San Miguel: Carambola, 30.XII.1982, *T. M. Pedersen 13470* (SI); Ruta Nacional 118 al NE de Loreto, 10.IV.2008, *M. Múlgura de Romero et al. 4450* (SI) – Santo Tomé: 16,2 km por ruta provincial 37 del cruce con ruta provincial 94 en dirección a Virasoro, 10.III.2006, *R. H. Fortunato et al. 9085* (BAB). Entre Ríos.Diamante:Diamante, I.1945, *M. M. Job 99* (LP). Misiones. No identified department:Arroyo San Juan, entre Candelaria y Santa Ana, X.1947, *E. Grondona & R. Spegazzini 1454* (BAB) – Candelaria:Loreto, 28.I.1907, *C. Spegazzini s.n.* (BAB 19192) – Capital:Garupá, camino al balneario, 20.I.1992, *G. J. Seijo 115* (BAB); Posadas, 14.I.1907, *C. Spegazzini s.n.* (BAB19881, 19883).

CERRADO

MACHAERIUM ERIOCARPUM

BRAZIL. Mato Grosso do Sul: Corumbá: estrada para Forte Coimbra, 14.XII.1986, *C. N. Da Cunha et al. 2113* (CGMS); Fazenda Acurizal–Nabileque, 31.VIII.1987, *A. Pott et al. 3267* (CGMS) – Ladário: Morro Ladário, 16.XI.1996, *M. A. O. Bezerra & J. L. Peixoto 7* (CGMS). Miranda: Margem da estrada do Carandazal, 03.XI.1990, C.A. Conceição 2737 (CGMS); BR-262, 40km de Miranda. Solo arenoso fértil, 18.XII.1990, *U. M. Resende et al. 360* (CGMS) – Nioaque: Assentamento Andalucia caminho do Taquarusul, X.2008, *F. Matos-Alves & L. C. S. Magalhães 103* (CGMS) – Porto Murtinho: Fazenda Anaí, 30.VIII.2008, *I. K. Mori 4* (CGMS); Fazenda Andrea I., 18.XII.2004, *L. E. A. M. Lescano 209* (CGMS);

Fazenda Boa Esperança, rodovia BR 267, km 20, leste de Porto Murtinho, 04.IV.2001, *A. L. B. Sartori et al. 453* (CGMS); Estrada 6, *G. P. Nunes et al. 111* (CGMS); Fazenda Agro Comercial Aubi, 09.V.2007, *F. Matos-Alves et al. 395* (CGMS); Fazenda Retiro Conceição, 16.XII.2008, *E. P. Seleme & A. B. L. Sartori 16* (CGMS). PARAGUAY. Central: Trinidad, I.1944, *Pavetti 11051* (AS). Concepción: 12 km. NE de Loreto, camino a Paso Barreto,17.XII.1983, *R. Vanni et al. 398* (MO); Puerto Fonciere, 29.XII.1947, *T. Rojas 14060* (MO); San Lázaro, I.1931, *Rojas 5507* (AS).

ERIOSEMA PLATYCARPON

BRAZIL. Distrito Federal: Chapada de Contagem, legume garden, experimental station, campus Univ. Brasilia, *Irwin 19504* (MO). Mato Grosso do Sul: Uncertain department (between Taquarussu, Jateí and Naviraí): Parque Estadual das Várzeas do río Ivinhema, 18.IV.2009, *R. B. Chaboco 9* (CGMS) – Aquidauana: Estância Betânia, 16.XII.2012, *P. I. Martins 37* (CGMS) – Bataguacú: Margem direita do rio Paraná, 08.IV.1999, *A. Amaral Junior 354* (CGMS) – Cáceres: Fazenda São Joao Lisito, próximo a Fazenda Santa Cecília, sub-regiao de Cáceres, Pantanal, 15.XI.2005, *A. Pott 13630* (CGMS) – Campo Grande: Reserva EMBRAPA, CNPGC, 29.I.2002, *J. F. Santos 94* (CGMS) – Corumbá: Fazenda Xaraés, 16.I.2000, *A. C. Araujo 748* (CGMS); Reserva do Amolar, Fazenda Acurizal, 08.V.2003, *A. Pott 11057* (CGMS); Capão 37, Pantanal de Miranda/Abobral, 14.XI.1997, *R. S. de Arruda 42* (CGMS); RRPPN Acurizal: Riacho Fundao, Reserva Ecotrópica, 28.XI.2010, *T. H. D. Leandro 126* (CGMS) – Inocencia: Fazenda Santa Claudia, próxima a rio Sucuriú, 12.II.2011, *A. Pott 16039* (CGMS) – Jateí: Parque Estadual das Várzeas do rio Ivinhema, 18.IV.2009, *R. B. Caboco 9* (BOTU) – Miranda: Agachi, rio Agachi, 17.III.2003, *G. Hatschbach et al. 74821* (MBM) – Nhecolandia: Fazenda Nhumirim (EMBRAPA), Reserva Biológica, 15.X.1990, *A, Pott 5690* (CGMS) – Tacurú: Tacuru, no date, *T. M. Pedersen 15972* (MBM). São Paulo: Novo Horizonte: Fazenda Rio Morto, Seter Figueira Blanca, no date, *H. T. Sujuki s.n.*(BAB). Tocantins: São Judas: Porto Nacional, 15.VIII.2016, *D. B. das Chagas 98* (HUEFS). PARAGUAY. Amambay: 43 km por Ruta 3, lugar del cruce con Ruta 5, *Schinini & DeMatteis 33749* (BAB). Canendiyú: 10 km S de Igatimí, 06.XII.1997, *Schinini & DeMatteis 33370* (BAB). Cordillera: Cordillera de Piribebuy, XII.1876-I.1877, *B. Balansa* 1541ª (MO); Ybytú Silla, 03.II.1991, *E. M. Zardini & U. Velázquez 26791* (MO). Itapúa: Isla Yacyretá, 21.I.1997, *E. M. Zardini & L. Villate 46240* (MO). San Pedro: Río Tapiracuay, 8 km Sa Estanislao, camino a Rosario, 27.II.1968, *A. Krapovickas 14291* (MO). ARGENTINA. Corrientes: San

Miguel: 12 km NE de San Miguel, 27.II.1990, *R. Vanni 1461* (MO). Misiones: Candelaria: Yabebyry, 23.IV.1945, *J. E. Montes 825* (MO).

MIMOSA SUBSERICEA

BRASIL. Distrito Federal: Ca. 1 km Barragem Paranoá, DF–9, 27.II.1970, *S. Irwin 26684* (NY) – Chapada da Contangem, ca. 10 km E of Brasília, 13.IX.1965, *H. S. Irwin 8263* (NY). Goiás. Río Tiquira, 18.IV.1967, *H. E. Heringer 11411* (NY) – Alto Paraíso do Goiás: Chapada dos Veadeiros, Ca. 16 km S of Alto Paraíso, 20.III.1969, *H. S. Irwin 24731* (NY) – Caldas Novas: At headwaters of the creek, Rio Quente, near hotel, "Pousada do Rio Quente", at foot of west slope of the Serra de Caldas (a flat-topped Batholithic dome), 13 km due WSW of city of Caldas Novas, 20.XII.1974, *E. P. Heringer 14107* (NY) – Calcavante: Chapada dos Veadeiros, ca. 10 km S of Calcavante, 08.III.1969, *H. S. Irwin 24055* (NY); Chapada dos Veadeiros, Ca. 37 km N of Veadeiros, 14.III.1969, *H. S. Irwin 24371* (NY); Serra do Morcego, Córrego Estrema, ca. 38 km NE of Formosa, 21.IV.1966, *H. S. Irwin 15206* (NY); Serra do Morcego, Córrego Estrema, ca. 42 km NE of Formosa, 20.IV.1966, *H. S. Irwin 15132* (NY) – Cocalzinho de Goiás: 15 km N of Corumbá de Goiás, on road to Niquelândia, in valley of rio Corumbá, Serra dos Pirineus, 16.I.1968, *H. S. Irwin 18652* (NY) – Corumbá do Goiás: Fazenda Cuiabá, Caverna dos Ecos, 23.I.1994, *V. V. Mecenas 51* (NY) – Cristalina: Serra dos Cristais, 20 km N of Cristalina, 07.III.1966, *H. S. Irwin 13717* (NY) – Flores de Goiás: Rod. BR-020, 2-3 km de JK, 22.I.1997, *G. Hatschbach 66096* (NY) – Formosa: Córrego Itaquera, ca. 30 km N of Formosa, 02.V.1966, *H. S. Irwin 15535* (NY) – São João de Aliança: Estrada para Vaozinho, 09.II.1994, *G. Hatschbach 60238* (NY). Mato Grosso. Barra de Garças: 266 km along new Road NNE of village of Xavantina, 06.IX.1968, *G. Eiten 8592* (NY); Ca. 35 km ENE of Barra de Garcas, 04.V.1973, *W. R. Anderson 9675* (NY) – Chapada dos Guimarães: Rodovia Chapada–Cuiabá, próximo do Portão do Inferno, 12.VIII.1997, *G. H. Hatschbach 66729* (NY); Vicinity of Burití, 12.II.1985, *E. C. de Oliveira Filho 260* (NY) - Xavantina: 4 km do Acampamento da expedição Inglesa, em direção Norte na estrada de Rodagem Xavantina-Cachimbo, 10.II.1969, *E. Onishi 915* (NY); Cachimbo Road, km 260, 04.XII.1967, *D. Philcox 3373* (NY) – Ribeirao Cascalheira: 8 km NE of the base campo of expedition, close to Xavantina-São Félix road), 12.IV.1968, *J. A. Ratter s.n.*(NY935916). Mato Grosso do Sul. Bonito: MS-382, 6,5 km W de Bonito,

09.XI.2002, *A. Pott et al. 10528* (MBM) – Camapuá: Open cerrado along highway BR-163, 07.II.1975, *W. R. Anderson 11241* (NY) – Caracol: 100 km SW de Jardim na rodovia BR-267 para Porto Murtinho, km 585, 16.IX.2002, *A. Pott Et al. 10975* (MBM) – Iguatemi: Fazenda Entre Rios, Rio Yhovy, 06.V.2009, *M. Morales et al. 715* (BAB) – Miranda: Carrapatinho, 13.II.1993, *G. Hatschbach 59055* (NY) – Rio Verde das Sete Quedas: Rio Verde, Sete Quedas, 08.VIII.1997, *G. H. Hatschbach 66758* (NY); Salto das Sete Quedas on the Rio Verde, 15 km SW of Rio Verde, 08.II.1975, *W. R. Anderson 11263* (NY) – Terenos: Terenos, 17.II.1990, *G. Hatschbach 23714* (NY). Minas Gerais. Formoso: Estrada entre a Chapada Gaúcha e cidade de Formoso, 30.XI.1997, *R. C. de Mendonca 3318* (MO); Parque Nacional Grande Sertão Veredas, 18.II.1999, *R. C. de Mendonça 3872* (NY); Parque Nacional Grande Sertão Veredas, próximo á entrada que dá acesso ao alojamento da Furnatura, 04.XII.1997, *R. C. de Mendonça 3354* (NY) – Joaquim Felício: Rio da Onça, 19.I.1996, *G. Hatschbach 64417* (NY); Serra do Cabral, 7 km W of Joaquim Felício, 07.III.1970, *H. S. Irwin 27142* (NY); Serra do Cabral, hills above Joaquim Felício, 13.II.1988, *W. W. Thomas 5953* (NY) – Paracatú: Serra da Anta, summit of Chapada, Ca. 10 km NW of Paracatú, 03.II.1970, *H. S. Irwin 25868* (NY) – Uberlândia: Jardim Umuarama, 19.I.1981, R. *C. Vieira 77* (NY) – Varzéa da Palma: Serra do Cabral, Agropecuária Serra do Cabral, 16.I.1996, *G. Hatschbach 64174* (NY). Pará. São Félix do Xingú: Reserva Florestal do Gorotire, 18.I.1983, *G. K. Gottsberger s.n.*(NY). Paraná. Arapotí: Fazenda Araporanga, 10.II.1997, *O. S. Ribas 1703* (NY) – Campo Mourão: Campo Mourão, Aeroporto, arredores, 17.V.1992, fl, *G. Hatschbach & L. R. Noblick 57062* (BAB, ny) –Ponta Grossa: Furnas, Vila Velha, 13.XII.1965, *R. Reitz 17502* (NY). São Paulo: Bataguacú: Near Highway BR-257, ca. 58 km W of São Paulo, 06.II.1975, *W. R. Anderson 11210* (NY) – Itapaetininga: Itapetininga, 02.II.1968, *O. Handro 2008* (SI) – Itirapina: K. 28, 21.II.1955, *A. S. Grotta s.n.*(NY) – Mogí-Guacú: Campos das Sete Lagoas". Fazenda Campininha, just north of Rio Moji-Guaçú, 10km N of Padua Sales, 03.XII.1959, *G. Eiten 1486* (NY) – Pirassununga: Near city of Pirassununga, 12.VI.1964, *D. O. Norris 3360* (NY) – Tatuhy: Campo Santa Cruz, 30.I.1918, *F. C. Hoehne 1411* (NY). Santa Catarina. Chapecó: Fazenda Campo São Vicente, 24 km W of Campo Eré, E of Road fork, no date, *L. B. Smith 11601* (NY). Tocantins. Pium: Ilha do Bananal, Parque Nacional do Araguaia, 27.III.1999, *R. C. de Mendonça 4081* (NY). PARAGUAY. Amambay:1,6 km al NE de la ruta en direcc. a P.J. Caballero, 08.III.2008, *R. H. Fortunato 8535* (BAB); Around Bella Vista Norte, 22º07’45’’S 56º30’00’’O, 12.I.2000, fl-fr, *E. M. Zardini & M. Vera 53514* (BAB); Around Cerro Corá, 22º39’53’’S

55º59’18’’O, 27.II.2001, fl-fr, *E. M. Zardini & A. Acosta 56092, 56119* (BAB); Colonia Fortuna-Guazú, 09.III.2008, *R. H. Fortunato et al. 9285* (BAB). Caaguazú: ±2 km E de Caaguazú, pot RN7, Barrio Tururú, Ayo. Tucurú, 08.III.2005, *R. H. Fortunato et al. 8535* (BAB) – San Pedro: 1 km al N del Barrio San Pedro, en direcc. a P.J. Caballero por ruta 3, 07.III.2008, *R. H. Fortunato et al. 9207* (BAB); 2 km N de Cororó hacia Cnia. Yby-Yahú, 15.II.1986, *R. H. Fortunato et al. 812* (BAB); Yaguareté Forest (Sustainable Forest Systems site), 23º46’53’’S 56º03’15’’O, 31.X.1996, fl, *E. M, Zardini & C. Balbuena 45781* (BAB); same locality, 17.I.1996, fr, *E. M. Zardini & S. Zavala 44207* (BAB).ARGENTINA. Corrientes. Ituzaingó: Ituzaingó, frente al cementerio, 15.II.2008, fr, *M. Morales et al. 632* (BAB). – Mburucuyá: Estancia Santa María, no date, *J. G. Fernández 842* (BAB); Parque Nacional Mburucuyá, 28.XII.2004, fl, *M. S. Ferrucci et al. 2208* (BAB) – San Miguel: RP118, a 2 km del cruce con RN12, 07.III.2007, *Morales & Seijo 266* (BAB). Misiones: San Ignacio: camino al Teyú Cuaré, 23.I.1987, *Fontana et al. 230* (BAB).

CHACO-ANDEAN

ACACIA VISCO

PERÚ. Cusco: Vilcabamba, Vilcabamba, Ututo, 20.X.2003, *E. Succlli et al. 1542* (MO). BOLIVIA. Cochabamba: Campero: Buena Vista, valles, 27.XI.1999, *C. Antezana Valera 1381* (BOLV) – Mizque: Mizquie, outside E of town, 27.XII.2002, *L. Rico & T. Windsor-Shaw 1199* (MO). Chuquisaca: Zudañez: Lambayo, 23.XI.1991, *Saravia Toledo 10075* (CTES). La Paz: Hacienda Huajchilla, 18 km al sudeste de La Paz (La Florida), a lo largo del Río La Paz., 18.XII.1986, *J. C. Solomon 15777* (MO). Santa Cruz: Bermejo: Bermejo, 0,5 km al N de Bermejo, no date, *Saldías & Grupo de Dendrología s.n.* (CTES) – Vallegrande, 15.II.1990, *I. R. Vargas 429* CTES) – Manuel María Caballero: 5 km (by air) SW of Comarapa on gravel road to Chilón, 4.5 km (by road) from highway at Comarapa, 26.XI.1999, *M. Nee 50668* (MO) – Vallegrande: Huasacañada, 15.II.1990, *I. R. Vargas 429* (CTES). Tarija: Arce: 5 km antes de llegar al abra de la Cruz, camino a Padcaya, 30.I.1998, *M. Liberman et al. 1909* (MO) – O’Connor: 3 km S de Entre Ríos, no date, no collector s.n. (CTES). ARGENTINA. Catamarca: Andalgalá, 01.X.1997, *P. Alayón 13* (CTES), 27.XI.1996, *C. A. O’Donnell s.n.*(CTES); Andalgalá, común en las quebradas, XI.1915, *Jörgensen 958* (MO); dry shrub forest between Andalgalá and Choya, 28.XI.1946, *E. Wall s.n.*(MO) – El Alto: Guayamba, 12.II.1947, *B. Ahumada 191*

(CTES); Fuerte de Andalgalá, XI.1872, *F. Schickendatz 55* (MO) – Santa María (referred originally to Tucumán, but probably wrong): Fuerte Quemado, 20.10.1948, *A. Schulz 7330* (CTES). Jujuy: Humahuaca, 14.I.1971, *A. Boelcke BAA7057* (CTES); Tilcara, 18.II.1971, *A. Boelcke 102* (CTES); Tumbaya, 21.XI.2001, *R. H. Fortunato 7429* (CTES); same locality,07.III.2002, *7645* (CTES); Vallegrande, 21.II.2008, *F. Zuloaga et al. 10296* (CTES). La Rioja: Uncertain department (between Famatina and Chilecito): Ruta 40, Plaza Vieja, entre Chilecito y Famatina 20.III.1960, *A. T. Hunziker et al. 15150* (CTES) – Capital: Sierra de Velasco, entre Dique Los Sauces y el cerro de La Cruz, a aprox. 1 km del último, 13.XII.1998, *F. Biurrun 2524* (CTES); Chilecito, 13.III.1989, *E. Aguirre 693* (CTES). Salta: *Nicora 9063* (CTES) – Cachi: La Paya, 4 km al W de RN40 y 8 km al S de Cachi, no date, *Novara 11540* (CTES) - Capital: Cerro San Bernardo, ladera S, sobre el camino carretero desde Portezuelo 29.III.2002, *Novara 11750* (CTES); Hills E of Salta (Barrio Tres Cerritos), 22.IX.1985, *Gentry & C. A. Palaci 51731a* (MO) – Chicoana: Ruta 59, quebrada de Escoipe, 23.II.1987, *E. Nicora 9063* (CTES) – Metán: Metán, 19.X.1988, *Del Castillo 1079* (CTES) – Rosario de Lerma: Ruta 51, camino de Ciudad de Salta a San Antonio de Los Cobres, 2 km antes de Chorrillos, 16.II.2002, *Cialdella 349* (CTES, SI), *R. H. Fortunato et al. 7254* (CTES). San Luis: Capital, 06.IV.1985, A. B. Guarmaschelli, 9 (CTES). Santiago del Estero: Copo, 01.XI.1974, *A. Schinini 10022* (CTES). Tucumán: Tafí del Valle, 01.I.1974, *A. Krapovickas 24510* (CTES); 19.X.1948, *Schultz 7292* (CTES), 09.II.1974, *T. M. Pedersen 10787* (CTES); Tapia, 27.X.1976,*Seigler & Vervoorst 10114* (MO); Trancas, 12.X.1925, *Schreiter LIL68590* (CTES).

GEOFFROEA DECORTICANS

PERÚ. Cusco: Anta: Mollepata, camino a Cunllac, 19.V.2006, *I. Huamantupa 7702* (MO).Tumbes: Hacienda La Choza, 08.II.1947, *O. V. Núñez s.n.*(SI16856). BOLIVIA. Chuquisaca: Comunidad Itacaray, 17.XII.2006, *J. A. Peñaranda et al. 264*[a] (MO); Huacareta. Comunidad Tacurbity. Alrededores de la comunidad, 25.VIII.2005, *J. Villalobos & L. Flores 204* (MO); Luis Calvo: Comunidad Arrayán, pie de monte de la serranía Inca Huasi, 16.X.2005, *A. Lliully et al. 327* (MO) – Sud Cinti: Cerca de la Comunidad Las Abras, Cañadón Pirua, 23.X.2005, *R. Lozano 1518* (MO). Santa Cruz. Unknown department (between Sara and Buena Vista): Entre Portachuelo y Bella Vista, 28.X.1980, *F. Zuloaga et al. 1489* (SI) – Andrés Ibáñez: Barrio Heroes del Chaco; 6 km SE (línea recta) de la ciudad de Santa Cruz; estancia "La Lechería", 26.IX.1990, *M. Saldías et al. 1188* (MO); Valle Grande, 19.VIII.1994, *B. Mostacedo C. et al. 2246* (MO) – Cordillera: Comunidad Cuarirenda, Izozog, al N de la Comunidad Cuarirenda, 15.IX.1998, *G. Bourdy 2036*

(MO); Iyobi, Ex10, 18.IX.1999, *M. Chiraye et al. 16* (MO); La Brecha, Bañados del Izozog, alrededores del hospital y 3 km E camino hacia río Parapetí, 03-05.VII.1991, *I. G. Vargas & E. Tapia 1053* (MO); Rancho Nuevo, Ex10, 26.X.1998, *M. Chiraye et al. 96* (MO); Manuel María Caballero: 6.4 km (by road) W of center of Saipina, on road from Saipina to Aiquile, valley of Río Mizque, 09.XII.2005, *M. H. Nee et al. 53722* (MO, NY); Vallegrande: Vallegrande, 19.VII.2010, *L. Arroyo P. et al. 5137* (MO). Tarija: Aniseto Arce: Entre La Mora y Padcaya, 22.X.1980, *F. Zuloaga et al. 1240* (SI); Tomatitas, 18 km NW of Tarija City, 19.IV.2003, *L. Rico et al. 1647* (BOLV, MO) – Cercado: 18 km hacia Entre Ríos, Santa Ana, no date, *S. Beck & M. Liberman 9636* (LPB); Tarija, 18 km hacia Entre Ríos, 22.X.1983, *S. Beck & M. Liberman 9636* (SI) – Gran Chaco: 15 km hacia el N de Yacuiba, Campo Pajoso, Lomas de Madrejones. 23.IX.1985, *S. Beck et al. 11503* (SI) – Tarija: Entre Concepción y Tarija, 22.XI.1980, *Zuloaga et al. 1249* (SI).CHILE. Antofagasta: El Loa, ruinas al O de San Pedro de Atacama, 16.II.1970, *Rodríguez & Rivera 24* (BAB) San Pedro de Atacama, 18.X.1961, *A. Garaventa H. 4282* (BAB). Atacama: Alto del Carmen: San Féliz, vereda enfrente de la destilería de pisco Horcón Quemado, 12.X.2010, *R. H. Fortunato et al. 9849 (BAB);* Copiapó, 08.II.1989, *J. Aronson 7731* (MO); Copiapó, piedra Colgada, 07.X.1966, *C. Giles 4978* (BAB); Copiapó, Valle, 28.IX.1953, *A. L. Cabrera 11367* (SI); Elqui Valley, about 12 km E of Vicuna, at Algarrobal, 02.VIII.2000, *L. R. Landrum & S. S. Landrum 9843* (MO); on the pass of Pajonales, 17.I.1992, *O. Zöllner 18315* (MO). Copiapó: Valle de Copiapó, cerca de Nantaco, 18.IX.1961, *F. Schegel 3859* (BAB). Coquimbo: Camino de Serena a Vicuña, 14.IX.1948, *F. Behn s.n.*(BAB); Coral Ovalle, Cabrería, 17.I.1949, *C. Jiles 1262* (BAB); Equi: Cerros Casablanca, along the road, 3 km west of Vicuña, 16.VIII.1939, *R. Wagenknecht 18413* (MO); Paihuano, 29.IX.1948, *F. Behn 22982* (BAB); Rivadavia, camino a Huantrea, 15.IV.1978, *G. Montero O. s.n.*(BAB).Tarapacá: Arica: Valle de Chaca, 40 km S of Arica. Fundo of Antonio Dekovic L., km 2015 on Pan-American Highway, 13.II.1989, *J. Aronson 7765* (MO); 1st Region. Valle de Azapa, 4-12 km E of Arica, 14.II.1989, *J. Aronson 7770* (MO); Quebrada Víctor Chaca, 23.IX.1955, *M. Ricardi 3456* (BAB). PARAGUAY. Alto Paraguay: Estancia "La Americana" aprox. 280 km NE de Filadelfia. A 100 m de la Administración, 16.IX.1986, *L. Pérez de Molas 1053* (MO). Boquerón: Colonia Neuland, línea 12 S, 01.IX.1992, *R. Degen 2709* (FCQ); Copoagro, km 589 Transchaco Highway, VII.1976, *P. N. Brandt 39* (SI); Estancia Catán, Cañada Lamadrid, 26.X.1994, *F. Mereles 5836* (CTES, FCQ, MO); Pirizal, 27.VIII.1992, *F. Mereles 4620* (FCQ). Presidente Hayes: Cruce Loma Plata-Ea Zalazar,

29.VII.1997, *E. M. Zardini & M. Vera 47104* (BAB); Estancia La Perla, 13.X.1986, *T. M. Pedersen 14603* (SI); Estancia Maroma, 19.X.2004, *M. Vera 83* (MO); Estancia Quebracho, Bañado Del río Pilcomayo, 22.IX.1993, *F. Mereles 5395* (MO); Estancia Santa Asunción, 25.X.2004, *M. Peña-Chocarro & J. De Egea Juvinel 1947* (MO); Estancia Tinfunque, 22.IX.1994, *F. Mereles 5797* (MO); Estancia Zalazar, 30.VII.1997, *E. M. Zardini & M. Vera 47190* (BAB); Km 92 de la RN9, III.2005, *R. H. Fortunato et al. 8584* (BAB); Palmar San Leonardo, ex Escalante, En bosque de inundación del antiguo Pilcomayo, 20.IX.1994, *F. Mereles 5770* (MO); Río Verde, cerca de Pozo Colorado, 11.X.1980, *J. Fernández Casas et al. 4466* (MO). ARGENTINA. Buenos Aires: Campana, 29.IX.1946, *A. T. Hunziker 1649* (MO) – Patagones: 25 km S of Carmen de Patagones, no da te, *H. A. Fabris & H. Schwabe 4913* (NY) – Saavedra: Sierra de Curamalal, 03.XI.1939, *A. L. Cabrera 5452* (SI) – Zárate: Zárate, barranca, 25.XI.2002, J. A. Hurrell et al. 4914 (MO, SI).Catamarca: Andalgalá: Común en cercos y en el campo seco. Andalgalá, 09.IX.1915, *P. Jörgensen 959* (SI) – Capayán: Huillapima, 06.IX.1960, *Marzocca s.n.*(BAB78157) – Capital: Ciudad de Catamarca, 15.XI.1946, *Dimitri & Piccinini 7* (BAB); La Brea, 30.XI.1946, *Dimitri & Piccinini 311* (BAB). Chaco: Primero DE Mayo: 24,7 km al S de Isla del Cerrito, 03.III.2006, *R. H. Fortunato et al. 8062* (BAB) – San Fernando: Barranqueras, IV.1983, *Martínez Crovetto s.n.*(SI). Corrientes: San Luis del Palmar, RP9, no date, *J. Greppi 36* (BAB). Córdoba. Capital: Camino a La Calera-Quebrada de las Rosas, no date, *no legible 10061* (BAB) – Colón: ASCOCHINGA, 30.X.1935, *M. L. Giardelli 50* (SI) – Ischilín: Quilino, 02.VI.1913, *no colector 136* (BAB) – San Alberto: Ca. 6 km NW of Villa Cura Brochero, Ruta Provincial 15, 13.XII.1978, *J. C. Solomon & A. Solomon 4122* (MO). Entre Ríos: Colón: Pueblo Liebig, no date, *D. Tosto s.n.*(BAB92307) – Diamante: Diamante, 01.II.1973, *A. Burkart 29338* (SI) – Gualeguay: Aldea Asunción, Estancia La Jarra, 23.II.2003, *J. Hurrell et al. 5032* (SI). Formosa: Laishi:Reserva Ecológica El Bagual. San Francisco de Laishi, 10.VI.1999, *DI Giacomo 420* (CTES). Jujuy: Uncertain department: Estes Hacienda, about 40 km north of Jujuy; along roadside dence, 05.X.1938, *W. J. Eyerdam & A. A. Beetle 22422* (MO) – Calilegua: Sierra de Calilegua, en los bosquies, 05.IX.1927, *S. Venturi 5251* (SI) – Capital: Cuesta de las Lajitas, 27.VIII.1981, *Ahumada et al. 4258* (SI) – La Caldera: RN9, Av. Palau y Av. Carmen Rudy, 23.IX.2005, *R. H. Fortunato et al. 12891* (BAB) – San Pedro: Ruta 34, ca. De San Pedro, 10.IX.1983, *A. L. Cabrera 34009* (SI) – Santa Bárbara: Camino a Agua Caliente, desde Caimancito, 27.IX.2015, *R. H. Fortunato et al. 12922* (BAB) – Tumbaya: Purmamarca, 21.XI.2001, *R. H. Fortunato et al. 7433* (BAB). La

Pampa: Caleu Caleu: Cerro de Los Viejos, Dto. 4, 24.III.1928, *Clos 3313* (BAB) – Hucal: Estancia La Rosada, 10.VI.1962, *Cano & Cámara 299* (BAB) – Loventué: Entre Casa Quemada y Victorica, X.1959, *Cano 343* (BAB) – Realicó: Realicó, FCO, 02.I.1929, *Clos 4236* (BAB) – Toay: Parque Luro, I.1960, *Cano 987* (BAB) – Trenel: Estancia Los Chañares, 1 km del ingreso a Trenel, 15.I.2005, *N. Paniego s.n.*(BAB92313) – Utracán: General Acha, en montes y suburbio, 06.XI.1953, *A. Burkart 19243* (SI). Mendoza: Lavalle: Ruta Nacional 142, km 37.5, al SW del río Mendoza en dirección a El Encón, 25.I.2008, *R. H. Fortunato et al. 9151* (BAB) – Las Heras: El Challao, 10.XI.1946, *Covas 18214* (SI) – Luján de Cuyo: Puntillas, 05.X.1993, *R. Sanzin 48* (SI) – San Rafael, no date, *G. Giráldez 17* (BAB54791). Río Negro: Adolfo Alsina: Camino entre Viedma y San Antonio, RN3 km 935, 13.XI.1963, *M. N. Correa et al. 2451* (BAB) – Avellaneda: Choele-Choel, 06.III.1913, *P. Jörgensen 3738* (BAB) – San Antonio: Cruce de entreada de San Antionio Oeste entre rutas 3 y 251, 12.XII.1994, *R. H. Fortunato et al. 4743* (BAB); San Antonio, 8 km de San Antonio al S, ruta 3, 08.X.1965, *M. N. Correa & Nicora 3191* (BAB). Salta: Cachi: Cachi, 03.XII.1960, *Ruiz Leal 21293* (SI); Puerta La Palle, entre Cachi y Molinos, Finca de Funes, 21.X.1948, *A. Burkart 17626* (SI) – Capital: Rotonda de Limanche, no date s.n. (BAB) – Chicoana: Calvimonte, 18.IX.1988, *V. Núñez 472* (SI) – General Güemes: Güemes, 18.XI.1942, *A. Burkart 13907* (SI) – La Poma: 21 km al N de Payogasta por Ruta Nac. 40, 13.XII.2002, *R. H. Fortunato et al. 7731* (BAB) – La Viña: Costado de ruta 68 km 129, entre Osara y Coronel Moldes, 5-6 km N de Cnel. Moldes, *Tolaba 1304* (BAB) –Rivadavia: La Merced Vieja, 7 km al norte de Santa Victoria Este, 25.XII.2002, *G. F. Scarpa 497* (SI) – San Martín: 5 km E de Campo Durán, frente a refinería REFINOL, 09.III.2001, *R. H. Fortunato et al. 7001* (BAB); Campo cultivado con poroto en General Ballivian, Sierra de San Antonio, 21.II.2015, *R. H. Fortunato et al. 10263* (BAB); Campo Durán, cerca de refinería, 20.V.2015, *R. H. Fortunato et al. 10260* (BAB) – Tartagal: Alrededores de la estación de tren de TARTAGAL, 08.III.2001, *R. H. Fortunato et al. 6971* (BAB). San Juan: Jáchal: San José de Jáchal, 28.II.1937, *Spegazzini s.n.*(BAB57709) – Valle Fértil: Salina de Mascasín, no date, *Piccinini & Leguizamón 1670* (BAB); Valle Fértil y alrededores, 7-8.VI.1933, *unknown s.n.*(BAB). San Luis: Pedernera: Entre Ruta 8 y Justo Daract, 29.XI.1969, *Cano 4300* (BAB) – Pringles: Embalse La Florida, 16.X.1989, *M.Múlgura 1104* (SI). Santa Fe: Nueve de Julio: Esteban Rams, 14.IX.1936, *A. Ragonese 2390* (SI); Independencia, 23.VIII.1987, *A. Krapovickas & R. Vanni 41899* (MO) – Vera: Las Gamas, 23.IX.1983, *J. H. Hunziker 10701* (SI). Santiago del Estero: Robles: Beltrán,

22.IX.1940, *R. Maldonado 417* (SI). Tucumán: Burruyacú: Dique El Cadillal, I.2000, *J. Greppi 3* (BAB); El Timbó, 10.X.1923, *S. Venturi 2490* (BAB) – Leales: Chañar Pozo, X.1919, *S. Venturi 477* (SI) – Tafí del Valle: Tafí, Amaicha del Valle, 31.I.1933, *Burkart 5181* (SI) – Trancas: Tapia, 20.II.1921, *S. Venturi 1121* (SI).

PROSOPIS STROMBULIFERA

CHILE. Coquimbo: Camino de Ovalle a La Chimba, 11.IV.1952, *Kausel 3300* (SI).Libertador General O'Higgins: Rancagua, 01.I.1905, *Bertero s.n.*(SI). Tarapacá: Iquique: La Huaica, cerca del pueblo, 11.V.1972, *Ricardi et al. 334* (BAB). ARGENTINA. Buenos Aires: Uncertain deparment (Between Patagones and Villarino): Desembocadura del río Colorado, II.1913, *P. Jörgensen s.n.*(BAB) – Villarino: Pedro Luro, no date, *Bartlet 19921* (SI). Córdoba: Ischilín: San José de las Salinas, no date, *Hosseus 687* (CORD). La Pampa: Uncertain deparment (Between Guatraché and Hucal): Entre Guatraché y Bernasconi, 17.XII.1951, *Ragonese & Piccinini 8124* (BAB) – Capital: Ruta 35, a ±10 km de Santa Rosa, 20.XII.1966, *E. Cano 3460* (BAB) – Chalileo: Santa Isabel, 18.III.1938, *A. L. Cabrera 4388* (SI) – Cura Có: Laguna Urre Lauquen, 12.XII.1966, *Cano 3437* (BAB) – Guatraché: Guatraché, 25.I.1934, *J. J. de Hernández-Burkart5956* (SI) – Lihuel Calel: Laguna La Asturiana, XI.1959, *Cano 419* (BAB). La Rioja: Coronel Felipe Varela: Villa Unión, no date, *T. Meyer 4149* (US) – Lamadrid: Villa Castelli, 14.I.1942, *T. Meyer 4066* (SI) – Patquía: Guayapa, III.1933, *Lahitte & Castro s.n.*(BAB448550) – Rosario Vera Peñaloza: Salina de Mascasín, en el terraplén de la vía del FC Belgrano, 13.XI.1970, *Piccinini & Leguizamón 1813* (BAB) – Vinchina: Vinchina, no date, *G. Covas 270* (NY). Mendoza: Capital: Vías al E de la capital, 04.IV.1937, *A. Burkart 8437* (SI) – Las Heras: Camino a Villavicencio, 13.II.2006, *A. Wulff et al. 1006* (SI) – Las Heras: Borbollón, 12.I.1948, *J. H. Hunziker 3155* (BAB); Campamento del Plumerillo, 01.XII.1955, *Ruiz Leal 3550* (SI) – Luján de Cuyo: Cerca del Dique Cipolletti, 26.I.1947, *G. Dawson & Pujals 1387* (BAB); Luján de Cuyo, 13.I.1908, *C. Spegazzini s.n.*(BAB22384) – Maipú: Barcala, no date, *no collector s.n.*(BAB); Pr. General Gutiérrez,18.XI.1933, *Ruiz Leal 1648* (SI) – San Rafael: Cuadro Bombal, 08.II.1947, *G. Dawson & C. Puyals 1681* (BAB71621); San Rafael, 18.XII.1927, *Güiraldes-Clos 3260* (BAB). Neuquén: Chos Malal: Río Barrancas, sobre camino a Buta Ranquil, 08.II.1950, *O. Boelcke et al. 4223* (BAB) – Pehuenches:

Barrancas, cerca del río, 23.XI.1982, *Rossow et al. 1794* (BAB) – Picún Leufú: Banda Mangrullo, Estancia El Mangrullo, 12.II.1956, *Mazzucconi 1163* (BAB). Río Negro: Uncertain department (between General Roca and Choele Choel): Camino Villa Regina-Choele Choel, 10.III.1949, *Illegible s.n.*(BAB73067) – Avellaneda: Coloia Josefa, II.1904, *C. Girola 95* (SI); RN22, km 1065, a 111 km de General Roca, camino a Choele Choel, 01.XII.2006, *Morrone et al. 5763* (SI) – Adolfo Alsina: Boca del Río Negro, 17.XII.1975, *M. C. Correa et al. 6828* (SI) – General Roca: Coronel J. F. Gómez, alr. E. Experimental, 12/25.II.1943, *R. A. Spegazzini 22* (BAB64592) – Pichi Mahuida: Buena Parada (próximo al Rí Colorado FCS), 24.III.1928, *E. C. Clos 3296* (BAB44456). Salta: Entre Cafayate y Santa Bárbara, 21.X.1948, *Ruiz Leal 12200* (SI). San Juan: Angaco: Angaco Norte, 21.I.1950, *Marzocca & P. Molinari 119* (BAB) – Iglesia: Reserva San Guillermo, valle del Río Blanco, margen derecha antes de Q. de Alcaparras, 17.II.1981, *Cajal et al. 54* (SI); Rodeo, cerca del puente, a orillas del río, 28.II.1989, *Molina et al. 4028* (BAB); Valle del Río Blanco, en las Juntas de La Palca, 21.XII.1981, *J. C. Pujalte 73* (SI) – Jáchal: Jáchal, 14.XII.1979, *A. L. Cabrera 31220* (SI) – Valle Fértil: Ruta 141 y empalme (510), km 137, no date, *Múlgura 4508* (SI) – Zonda: Pachaco, 23.II.1967, *A. L. Cabrera ET AL. 17914* (SI). San Luis: Ayacucho: Pampa de las Salinas, Arroyo Botija, NW de San Luis, ilegible s.n. (SI) – Capital: Alto Pencoso, II.1919, *Brach s.n.*(SI) – Pedernera: El Morro, no date, *Pastore 29* (SI); Entre Estancia Crámer y Villa Reynolds, 12.II.1971, *B. Vulleismier 1010* (SI). Santiago del Estero: Belgrano: Fortín Inca, FCCN, 09.IV.1919, *Girola s.n.*(BAB54604). Tucumán: Tafí del Valle: Amaicha del Valle, 03.II.1933, *A. Burkart 5494* (SI); Tiopunco, R307, 7 km N de Amaicha del Valle, entre mojones 125 y 126. Camino vecinal,11 km desde la ruta, 22.II.2000, *L. Anzoategui & L. Mautino 363* (SI).

SEASONALLY DRY TROPICAL FORESTS

AMBURANA CEARENSIS

BRAZIL. Acre: Brasiléia: Seringal Porongaba, Colocacão São José, 29.V.1991, *D. C. Daly Et al. 6789* (MO). Bahía: Uncertain deparment: 30 km na estrada de Brumado para Livramento do Brumado,

12.III.1991, *H. S. Brito & G. P. Silva 292* (K); Basin of the Upper São Francisco River, just beyond Calderao, 32km NE from Bom Jesus da Lapa, 22.VII.1981, *R. M. Harley Et al. 21516* (K) – Aracatu: Estrada que liga Unburanas a Ourives, 27.III.1984, *J. E. M. Brazao s.n.* (NY452232) – Barreiras: Ca. 10 km. W of Barreiras, 02.III.1971, *H. S. Irwin s.n.*(NY452224) – Bom Jesus da Lapa: Bom Jesus da Lapa, 18.IV.1980, *R. M. Harley Et al. S.n.*(L7044435) – Tucano: Tucano: Cachoeira do Inferno, às margens do Rio Itapicuru, 06.IX.2007, *D. Cardoso & A. M. Bastos 2056* (MO) – Ceará: Campos Sales: 2-4 km S of Campas Salas, 15.II.1985, *A. H. Gentry ET AL. 50127* (MO) – Quixadá: Quixadá, 06.VII.2008, *A. Ducke 1110* (G). Goiás: Calcavante: km 15 da estrada Teresina de Goiás/Cavalcante, 29.IV.1996, *B. A. S. Pereira s.n.*(NY982534) – Posse: km 19 da estrada Posse/Iaciara, 28.IV.1996, *B. A. S. Pereira s.n.*(NY982559). Mato Grosso do Sul: Corumbá: Corumbá. Close to the airport (Morro de Zé Feliciano), 08.X.1985, *J. A. Ratter 5128* (NY) – Ladário: Fazenda Uruba, 08.V.1994, *G. Hatschbach 60801* (MO). Pernambuco: Mirandiba: Serra das Umburanas (afloramentos rochoso), 23.IV.2007, *E. Córdula s.n.*(NY1019316). Piauí: PI-115, entre Campo Maior e Castelo do Piauí, 11.VI.1979, *E. Nunes & A. J. Castro 6472* (MO). Rio de Janeiro: Nova Friburgo: Arredores da Pedra Riscada, 01.I.1988, *A. Ducke 1965* (K).Rondonia: Alvorada do Oeste: Linha 64, a 04 km. da BR-429 em direção a Costa Marques, 01.V.1987, *C. A. Cid Ferreira 8980* (MO). Tocantins: Aurora do Tocantins: km 69 da estrada Taguatinga/Combinado, 16.VIII.1995, *B. A. S. Pereira s.n.*(NY982509). PERU: Cusco: La Convención: Dist. Echarate, San Antonio, Cirialo, 25.III.2006, *I. Huamantupa et al. 7387* (MO); Dist. Echarate, San Antonio, río Sapapoari, 23.III.2006, *J. Farfán Et al. 968* (MO). Loreto: Maynas: Mishana area, no date, *F. Ayala 5976* (MO). San Martín: Chazuta, Río Huallaga, no date, *G. Klug 4064* (MO); Mariscal Cáceres: Rio Huallaga, margen derecha; Balsa Probana; dtto. Tocache Nuevo, no date, *collector unknown USFPL 16755* (US). Pasco: Oxapampa, Shiringamazu, ca 20 km S of Iscozacin, Río Palcazu Valley, 08.VII.1988, *A. Gentry et al. 63437* (MO). BOLIVIA. Chuquisaca: Belisario Boeto: 30 km pasando el Rio Grande en el camano Vallegrande-Villa Senoño, 21.X.2001, *I. Vargas et al. 6289* (MO). La Paz: Chaquimayo-Tuichi trail ca 20 km NW of Apolo, disturbed dry forest along Río Machariapo, 12.VI.1990, *A. Gentry & R. Foster 71139* (BAB) – Franz Tamayo: Area Natural de Manejo Integrado Madidi, Asariamas, al Norte de Apolo, 21.V.2005, *A. Fernández Et al. 36* (MO); Area Natural de Manejo Integrado Madidi, Azariamas, 2 km al E del arroyo Javillas, 27.XI.2005, *L. Cayola et al. 2235* (MO); Parque Nacional y Area Natural de Manejo Integrado Madidi, NW de Apolo,

06.III.2005, *D. Choque et al. 29* (MO); Area Natural de Manejo Integrado Madidi, Virgen del Rosario, arroyo Yarimita, 17.III.2005, *J. Uzquiano et al. 134* (MO); Parque Nacional Madidi, camino Apolo-Azariamas, arroyo Pintata. A 314 m del campamento en direccion SE, aproximadamente a 15 minutos saliendo del camino, 20.II.2003, *L. Cayola et al. 69* (MO); Parque Nacional Madidi, río Quendeque, detrás del campamento Retamas, 28.I.2002, *D. De La Quintana 155* (MO); Parque Nacional Madidi, Sipicuyo, 1,7 km al N de Virgen del Rosario, 06.XI.2003, *A. Fuentes Et al. 5653* (MO) – Larecaja: Localidad Muchanes. En la pampa o planura, cruzando Chinendo, al NW de la Comunidad Muchanes, 30.IV.1994, *L. Vargas et al. 1212* (MO) – Sud Yungas: cuenca del Rio Boopi, La Asunta cerca de Evenay, 27.VII.1939, *B. A. Krukoff 10596* (MO). Santa Cruz: Chiquitos: Proximidades de la zona donde corta e camino de San José de Chiquitos a las salinas de San José, con la brecha del gasoducto principal a Corumbá, no date, *Fuentes s.n.*(USZ); Valle de Tucabaca; 10 km al SW del Río Tucabaca, no date, *I. Vargas et al. 3447* (MO); Valle de Tucabaca. A 20 km. al W del pueblo de Santo Corázon. Tramo 5 km. al NE del campamento Los Murciélagos, 24.X.1994, *Vargas Caballero s.n.*(NY452228) – Cordillera: Cabezas, 11.III.1945, *I. Peredo s.n.*(NY452227); Parque Nacional Kaa Iya del Gran Chaco, 4 km al NW de Palmar de Las Islas, 11.II.1998, *A. Fuentes & G. Navarro 2239*[a] (MO) – Ñuflo de Chávez: Las Trancas, Lomerio, 29.V.1998, *B. Mostacedo 3703* (MO); Las Trancas, Lomerio, parcelas de Bolfor, Las Trancas-95, *A. Jardim & G. Moscoso 1659* (MO); Lomerio. A 12 km al Norte de la comunidad Las Trancas, 03.VII.1995, *F. Mamani 928* (MO) – Velasco: Hacienda Acuario a 24 km de San José de Campamento, camino hacia el empalme a Piso Firme, 30.IV.1996, *J. Guillén et al. 29* (MO); Parque Nacional Noel Kempff Mercado. 15 km SE del campamento Los Fierros, sobre el camino al aserradero Tarbo, 02.XI.1993, *Saldías et al. 3363* (MO);Parque Nacional Noel Kempff Mercado, Cerro Pelao, no date, *A. Jardim 513* (MO). PARAGUAY. Alto Paraguay: Fuerte Olimpo, 26.III.1980, *L. Bernardi 20316* (MO); Proposed Biosphere Reserve Gran Chaco Americano: Agua Dulce, 08.II.2002, *E. M. Zardini & I. Apestegui 58312* (MO); Proposed Biosphere Reserve Gran Chaco Americano: Cerro León, 10.II.2002, *E. M. Zardini & J. C. Rivas 58524* (MO). Amambay: Bella Vista, 2 km S of town. Ranch of Félix Ocariz, 22.III.1983, *W. Hahn et al. 1276* (MO); Cerro Corá, allende el río Aquidabán, en colina, no date, *Bernardi 19177* (G). Cordillera: Instituto Agrícola Nacional. Caacupé, 80 km. E. de Asunción., 09.II.1984, *L. Elbert et al. 40164* (MO). ARGENTINA. Jujuy: Fraile Pintado, 02.IV.1941, *S. Zabala 111* (SI).

ENTEROLOBIUM CONTORTISILIQUUM

BRAZIL. Alagoas: Quebrangulo: Reserva Biológica de Pedra Talhada, *A. C. Cervi S.N.*(NY); Reserva Biológica de Pedra Talhada, Agreste, 14.XII.1994, *A. C. Cervi Et al. 7300* (NY). Bahia: Uncertain department: 5 km da cidade na estrada para Rio de Contas, 25.X.1988, *R. M. Harley et al. 25612* (K) – A ca. 500m da margem esquerda do Rio São Francisco, logo apos Ibotirama, a ca. 200m da ponte sobre o Rio São Francisco, na estrada para Barreiras (BR-242), 11.X.1994, *L. P. de Queiroz s.n.*(NY408743) – Caetité: Estrada Igaporã-Caetité, km 67., *L. Coradin et al. 4373* (K) – Feira de Santana: Campus da UEFS. Próxima da guarita da entrada do Campus, 05.XI.1991, *L. P. de Queiroz s.n.*(NY408742) – Gentio do Ouro: Mirorós, área do Projeto de Irrigação da CODEVASP., *A. L. Brochado & P. E. N. Silva 174* (K) – Jequitinhonha: Rod. BR-367, 10-15 km L de Jequitinhonha, 16.V.1988, *G. Hatschbach et al. 52190* (MO) – Livramento da Brumado: 5km da cidade na estrada para Rio de Contas, 25.X.1988, *R. M. Harley et al. s.n.*(NY408741) – Oliveira dos Brejinhos: Córrego Serra Negra., 12.X.1981, *G. Hatschbach 44166* (NY) – Riachão das Neves: Ca. 11km N de Riachao das Neves, na BR 135 (estrada Formosa do Rio Pret-Barreiras), 11.X.1994, *L. P. de Queiroz s.n.*(NY408744). Distrito Federal: Brasília: Between Fercal and Brasilia, 11.X.1963, *B. Maguire s.n.*(NY924342). Ceará: Fortaleza: Near Lagoa Maraponga, Parangaba, 22.X.1935, *F. E. Drouet s.n.*(NY924384). Goiás: Campinaçu: Próximo a foz do cor. Palmeiras. Local atingido pelo lago da UHE Serra da Mesa, 11.IX.1997, *S. P. Cordovil-Silva s.n.*(NY924355) – Iaciara: Km 15 da estrada Posse/Iaciara, 20.X.1995, *B. A. S. Pereira s.n.*(NY924198); Km 11 da estrada Nova Roma/Terezina de Goiás, 13.IX.1996, *B. A. S. Pereira s.n.*(NY924349) – Ipameri: Fazenda do Sr. Emerson Fitipaldi., 28.IX.1995, *T. B. Calcavanti s.n.*(NY1144390); Margem esquerda do Rio Corumba. 1 km da ponte Sao Bento, 26.IX.1995, *T. B. Calcavanti s.n.*(NY924353) – Niquelândia: Fazenda São João. Corrego do 'Val' (margem esq. do trairas); afluente do Rio Trairas; a 5 km de Indianapolis. Bacia de inundação da UHE Serra da Mesa, 09.VI.1992, *B. M. T. Walter s.n.*(924351); Reservatório em formação do AHE Serra da Mesa. Região do Rio Maranhão próxima ao Rio das Almas, 15.X.1997, *B. M. T. Walter s.n.*(NY924354). Maranhão: 10 km from Loreto on the road to São Felix, no date, *S. G. M. Bridgewater 785* (E). Mato Grosso: Acorizal: 100 km north of Cuiabá, en route to Diamantino, Brasilia-Acre Highway, 29.X.1963, *B. Maguire s.n.* (NY924362) –

Xavantina: By Rio das Mortes, just above Ferry, Xavantina, 12.IX.1967, *G. C. G. Argent s.n.*(NY924352). Mato Grosso do Sul: Antonio João: Antonio João, 5 km O, 16.X.1984, *G. Hatschbach 48541* (NY924319) – Aquidauana: Fazenda Salina, Pantanal do Rio Negro, 02.IX.1987, *B. Dubs s.n.*(NY924318). Minas Gerais: Uncertain deparment: APA Carste de Lagoa Santa, Matozinho, Fazenda Cauaia, 30.IX.1996, *A. E. Brina s.n.*(NY1144915) – Caratinga: Est. Biológica de Caratinga, 1a estação de coleta, trilha b, 17.X.1990, *E. M. Bacarica s.n.* (NY924322) – Grão Mogol: Estrada Porto Mandacarú - Grão Mogol, 20.VII.1985, *G. Martinelli s.n.*(NY924348) – Ituitaba: Carmo, 11.IX.1948, *A. L. Mesquita s.n.*(NY924347) – Nova Ponte: Nova Ponte, 25.IX.1996, *E. T. Neto s.n.*(NY924321) – Uberlandia: Fazenda Buriti, Uberlândia, 24.IX.1994, *G. M. Araújo s.n.*(NY924324) – Virgem da Lapa: Estrada Virgem da Lapa-Araçuai, km 10, 08.IV.1983, *H. C. de Lima s.n.*(NY1018198). Paraná: Candói: Rio Jordão, próximo ao Salto do Cachorro, no date, *G. Hatschbach Et al. 64472* (MO) – Tibagi: Estr. Tibagi-Castro, Rio Tibagi, 01.XI.1964, *G. Hatschbach s.n.*(NY). Rio Grande do Sul: Erechim: Erechim. RS 135, 11.VI.1993, *A. Butzke Et al. S.n.*(LEB) – Farroupilha: Farroupilha, no date, *R. Wasum 8968* (MO) – Porto Alegre: Morro da Polícia, 17.XI.1948, *B. Rambo 38243* (SI) – São Leopoldo: Vicinity of Sao Leopoldo, no date, *J. Eugenio Leite s.n.*(NY408732) – São Francisco de Paula: Estrada para Taquara no topo do morro, 31.XII.2000, *R. Wasum 871* (MO). Santa Catarina: Itajaí: Braia Braba, 15.VII.1953, *Reitz s.n.*(NY408735) – São Miguel do Oeste: Road from São Miguel, to Fr. Westphalen, km 39, 11.XII.1966, *J. C. Lindeman s.n.*(NY408734). São Paulo: Piracicaba, Piracicaba, 10.X.1967, *H. de F. Leitão Filho S.N.*(NY408733).BOLIVIA.Beni. Madidi: Arroyo Rudidi, sobre el Rio Tuichi, 17.II.2002, 14.XII.1994, *A. Araujo Et al. 402* (MO). Chuquisaca: Hernando Siles: no explicit locality, 14.XII.2006, *E. Portal et al. 92* (MO); Parque Nacional y Area de Manejo Integrado Serranía del Iñao. Comunidad Los Pinos, 26.IV.2003, *M. Serrano & J. Villalobos 4213* (MO) – Luis Calvo: Parque Nacional y Area de Manejo Integrado Serranía del Iñao. Comunidad Ticucha, 05.IV.2003, *M. Serrano et al. 4191* (MO) – Sud Cinti: Culpina, Cañón verde, sector las abras, 18.V.2005, *A. C. Carretero s.n.*(AAU); Culpina. Laderas bajas cercana al palmar, 08.V.2005, *A. C. Carretero 1522* (AAU); Culpina. Sector Arborito, Cdd. Manzanal, 17.VI.2005, *A. C. Carretero 1808* (AAU); Naranjitos. Sendero ca. 3 Km., al SW del Rancho de Don Fernando, 30.IV.2005, *R. Lozano & F. Tarifa 1238* (MO) – Luis Calvo: Municipio Villa Vaca Guzman, Canton Iguembe, 15.X.2007, *M. Jiménez et al. 394* (MO). La Paz: Franz Tamayo: Parque Nacional Madidi, río Hondo, senda turística que sale al Tuichi. , 11.IV.2002, *F. Bascope et*

al. 36 (MO) – Sud Yungas: 2 km a N desde el centro de Chulumani, no date, *Nee & Solomon 32014* (MO). Santa Cruz: Andrés Ibáñez: 12 km E del centro de Santa Cruz, 10.X.1989, *M. Saldías 080* (CTES); Avenida Pirai, W side of city Santa Cruz, 15.X.1990, *M. Nee 39282* (CTES); Santa Cruz de La Sierra, 29.III.1996, *M. Menacho & A. Jiménez 779* (CTES); Urbanización Monteverde, 06.V.1988, *Saldías 329* (CTES) – Chiquitos: Cerro Bocamina, Serranía de Sunsas, Tucavaca, el área de exploración de RTZ/Comsur, 05.VII.1995, *A. Jardim & A. Cadden 2226* (NY) – Cordillera: Charagua, 7 km NW del pueblo, orillas del río Ovai, 12.IV.1990, *I. R. Vargas 439* (CTES); Cuevo, 31.X.1993, *Saravia Toledo 12036* (CTES); Ibasiriri Izozog, cerca de la comunidad de Ibasiri, a 3 km de la Brecha, 20.V.1999, *A. Serato 2560* (MO); en los alrededores de la comunidad de Iyobi hasta 3 km al Oeste, no date, *M. Chiraye et al. 10* (MO); La Brecha, Bañados de Izozog, alrededores del hospital y 3 km E hacia el río Parapetí, *I. R. Vargas & E. Tapia 1034* (CTES); Progresiva de entre San Juan y el campamento Don Mario.Kp142+450-550, 09.XII.2001, *Y. Roca et al. 1481* (MO); Región de Lomerío. Cerca de la Comunidad San Antonio, 12.XII.1993, *M. Toledo 324* (CTES) – Florida: 1 km al este de Hierba buena por la carretera entre Santa Cruz y Comarapa, 05.VI.1992, *T. Killeen & I. Vargas 4042* (MO); along río Quirusillas [río Mairana], 1,1 km of highway on dirty road to Villa Ecce Homo, 08.V.1998, *Nee 49272* (CTES); Bellavista, 12.VI.2006, *M. Vargas 46* (MO); Carretera Comarapa-Santa Cruz, 98 km de Comarapa, 03.VII.1989, *D. N. Smith Et al. 13612* (CTES); Localidad de Bella Vista, camino entre la escuela y la entrada del sendero ecológico Cola de Mono, 05.II.2006, *D. Villarroel et al. 297* (MO); Samaipata, km 120 al SW en la carretera antigua Santa Cruz-Cochabamba, no date, *I. G. Vargas C. 828* (MO) – Germán Ibáñez: El Carmen, Ea. Campo en medio a 23 km SE de El Carmen, 02.V.1997, *Gutiérrez et al. 2109* (CTES) – Ichilo: Along road to Palacios, 2.5 km NNW of Montero-Buena Vista highway, 11.VI.1998, *H. M. Nee s.n.*(NY309795) – Ñuflo de Chávez: Región de Lomerío, a 5 km de la Plaza de la comunidad de Florida, 29.XI.1994, *M. E. Toledo 521* (CTES); Yendo a Sindicato San Salvador a 15 km al Este de El Puente, 08.II.1994, *J. Aguirre & M. Hurtado 3539* (MO) – Velasco: 50 m en al senda a la bahía, al E del campamento La Toledo, 16.IX.1995, *P. F. Foster et al. 138* (MO); Campamento EL REFUGIO a 1400 m al sudoeste de la casa yendo hacia la pampa de Las Islas, 23.V.1995, *R. Guillén & S. Coria 1490* (MO); Campamento TOLEDO, a 2500 m SE de la casa, 09.VI.1994, *R. Guillén & V. Choré 1801* (MO); Parque Nacional Noel Kempff Mercado. Campamento Cerro Pelao; 5 km del aserradero sobre el camino a El Empalme, 29.III.1994, *M. Saldías et al. 3616* (MO). Tarija: Aniceto

Arce: R.N. Flora y Fauna Tariquíua. Campamento en la Comunidad Salinas - La Misión, 25.XI.1998, *Z. N. Paniagua & K. Rodríguez 1405* (CTES); Mun. Padcaya. Cantón Emborozú. Reserva Natural Alarachi. Zona Cayotal. Río Emborozú Chico, 22.IV.2004, *M. Serrano et al. 4982* (MO). PARAGUAY. Uncertain department (between Central and Cordillera): Paraguaria Centralis: In regione lacus Ypacaray, no date, *Hassler 12262* MO). Caaguazú: Between Coronel Oviedo and Caaguazú; Route 2, km 148, 09.XI.1993, *E. M. Zardini & L. Guerrero 37175* (BAB, MO). Caaguazú: cerca de Mbutuy, 13.X.1980, *J. Fernández Casas & J. Molero 4233* (MA). Caazapá: Tava'i, 01.XI.1988, *I. Basualdo 1762* (MO). Canendiyú: Mbaracayú Natural Reserve, administered by Fundación Moisés Bertoni. Ñandurokai. Cerrado scrub., 31.X.1998, *E. M. Zardini & I. Chaparro 49472* (MO). Central: Border Tavarory-Acosta Ñu, 1 km E of road Ytororó-Tavarory, 16.VI.1994, *E. M. Zardini & L. Guerrero 39602* (BAB, MO); Border Tavarory-Acosta Ñu. Creek, affluent of Paraguay River, 10.VI.1993, *E. M. Zardini & V. Jara 38109* (BAB, MO); Nueva Italia, 14.III.1985, *L. Pérez de Molas 453* (MO);Ñemby, a 20-m del Centro, 20.VIII.1981, *I. M. Vavrek 293* (MO); San Lorenzo, campus universitario, 26.X.1984, *I. Basualdo 1163* (CTES). Concepción: Arroyo Pitanohaga, 01.VII.1994, *E. M. Zardini & L. Guerrero 39868* (BAB, MO); Estancia Primavera, 28.VI.2002, *E. M. Zardini & L. Guerrero 59013* (BAB, MO); Horqueta, 06.XI.1985, *Mueller 4* (MO), same locality, 09.III.1986, *Mueller 29* (MO). Cordillera: Caacupe-IAN, 09.II.1984, *Little 40166* (MO); Colonia Ojopoy, al E de Piribebuy, 03.VI.1985, *P. Arenas 2932* (CTES). Guairá: Cerro Nellville, 5 km E de Mboyacati, no date, *A. Schinini et al. 27682* (CTES); Tororo, Camino a cerro Acati, 14.XII.1988, *Soria 2964* (MO); Villarrica, salida de la ciudad, Cordillera del Ibytyrusu, cerro Polilla, 20.IV.1995, *F. Mereles & M. Soloaga 7555* (CTES). Itapúa: Isla Yacyretá, 26.V.1988, *Spinzi 29* (MO). Misiones: Yacyreta. Administración, 07.IV.1988, *Keel 1370* (MO). Paraguarí: Cerro Mbatoví, 09.VIII.1988, *I. Basualdo 1597* (MO); Compañía Costa Segunda, Cerro Palacios, *E. M. Zardini & M. Ortiz 3092* (MO); Paraguarí, 24.IX.1983, *I. Basualdo 693* (MO); Valle Apu'á, Colonia Achotei, estancia Lago Ypoá, 08.XI.2000, *F. Mereles et al. 8219* (CTES). San Pedro: Around Antequera, 23.VI.2006, *E. M. Zardini & L. Guerrero 56538* (BAB, MO); Around Ybapobo, 02.III.2001, *R. H. Fortunato et al. 56498* (MO). ARGENTINA. Buenos Aires: San Pedro: Entre San Pedro y La Mendieta, no date, *A. L. Cabrera 31026* (SI). Chaco: Comandante Fernández: EEA Sáenz Peña, 02.X.1989, *Bordón s.n.*(CTES5639) – San Fernando: Barranqueras, Paranacito, no date, *G. Rotta 422* (CTES). Corrientes: Capital: Perichón, 31.X.1975, *Ansotegui & Schinini 273* (CTES) – Empedrado: río

Empedrado, Ruta Nac. 12, 26.XI.1971, *A. Krapovickas et al. 19913* (CTES) – General Paz: 12 km E de Itá Ibaté, costa del río Paraná, 09.IV.1972, *Mroginsky et al. 695* (CTES) – Mburucuyá: Estancia Santa Teresa, 24.X.1976, *A. L. Cabrera 28194* (MO) – San Cosme: 30 km E de Corrientes por Paso de la Patria, 30.IX.1987, *Gentry et al. 59846* (CTES); Ensenada Grande, ruta 12, 06.X.1971, *A. Krapovickas 20000* (CTES) – San Miguel: Carambola, 02.II.1972, *T. M. Pedersen 10237* (CTES) – Santo Tomé: Ea Beltrán, Infrán Cué, 23 km W de Virasoro, 07.04.1992, *S. Tressens et al. 4003* (CTES). Formosa: Laishi: Reserva El Bagual, 10.X.2001, *Di Giacomo 529* (CTES). Formosa: Formosa: 21 km N of Formosa, 01.XI.1988, *J. Aronson 7669* (MO); 3 km N of city of Formosa, 12.III.1989, *J. Aronson 7839* (MO); Estancia Guaycolec, 14.X.2004, *M. Peña-Chocarro et al. 1870* (MO); Fortín Belgrano, no date, *G. Scarpa 736* (SI) – Pilagá: Espinillo, Colonia San Rafael, 26.X.1995, *A. Schinini 30376* (CTES) – Pilcomayo: La Frontera, 09.XI.1949, *I. Morel s.n.*(CTES); Sol de Mayo, 14 Km SW de Laguna Blanca, no date, *A. Schinini 30337* (CTES). Entre Ríos: Islas del Ybicuy: Delta, Arroyo Martínez, no date, *A. Burkart 15086* (SI) – La Paz: Estancia "La Invernada", no date, *A. Burkart 21220* (SI). Jujuy: El Carmen: El Carmen, 26.XI.1976, *A. L. Cabrera & H. A. Fabris 21143* (MO) – Ledesma: Ruta Prov. 31, 2 km del desvío de la Ruta Prov. 1 camino a Vinalito, 21.II.1998, *O. Morrone et al. 2880* (SI). Misiones: Cainguás: Predio UNLP: valle del arroyo Cuña Pirú, picada en selva, ruta 7, cerca del balneario, no date, *F. Biganzoli et al. 583* (SI) – Candelaria: Loreto, 13.XI.1931, *G. Grüner 3* (MO) – Capital: Posadas, in fruticetis humidis prope La Granja praedium, no date, *Ekman 1729* (MO) – Guaraní: Predio Guaraní. Sendero Mbya a 20 m de camino al ayo. Soberbio, 17.IX.1998, *S. Tressens 6087* (CTES) – San Ignacio: Parque Prov. Teyú Cuaré, Peñón de la Reina Victoria, 21.II.1996, *O. Morrone et al. 1093* (SI); San Ignacio, 26.XI.1949, *Bertoni 4678* (CTES). Salta: Anta: Parque Nacional El Rey, between intendencia and Quebrada Aguas Negras, 24.IX.1985, *A. Gentry et al. 51801* (MO) –Candelaria: Agua Caliente, 28.X.1927, *S. Venturi 5362* (NY) – Capital: Río Vaqueros, 5 Km. al E. del puente Ruta 9, no date, *L. Novara 2161* (MO) –Güemes: Trayecto por RP11, 20-30 km, entre 5 km al E de Estación ferroviaria Mojones y Campo Santo pasando por Betania, 22.IV.2000, *no colector s.n.*(CTES) – Rivadavia: El Colgado, 23.X.2009, *M. E. Suárez 298* (CTES) – San Martín: 3 km de la Ruta Nac. 34 camino de Piquirenda a El Chorrito, Snía. de Tartagal, 04.V.2003, *O. Morrone et al. 4592* (SI) – Orán: Ruta Prov. 18, a 3-4 km del Puente Internacional Argentina-Bolivia, camino a la Finca Yacúlica, no date, *O. Morrone 4522* (SI).

AMAZONIAN

CYNOMETRA BAUHINIIFOLIA

VENEZUELA. Amazonas: Autao, Río Sipapo, vía Boca de Cuao, 19.XI.1999, *A. Castillo 5398* (MO). Bolívar. Uncertain deparment: Locally frequent along Cano Coroso (Rio Caripo) between Rio Orinoco and Lago Coroso, 10.I.1956, *J. J. Wurdack 41194* (MO) – Aripao: Río Caurae, aguas debajo de Maripa-La Aurora, 25.VIII.1995, *J. Rosales 1679* (MO) – Ceceno: Fráneo La Urbana, bosque de revalse en planicie aluvial del Río Orinoco, en las inmediaciones del puerto El Jobal, 20.I.1989, *N. Cuello 703* (MO); Paso de Caruachi, río Caroní. 75 km E de Ciudad Bolívar , 31.III.1985, *B. K. Holst et al. 1968* (MO). GUYANA. Curuyuni-Mazarami. Essequibo river, 6-8 km downstream of Omai, 01.VI.1989, *L. G. Gillespie 1556* (INPA). Cuyuni-Mazarumi: 6-8 km downstream of Omai, 01.VI.1989, *L. Guillespie 1556* (MO). East Berbice: Corentyne River, Warakabra Rapids, 29.III.1995, *Mutchnick 1112* (MO). Essequibo: Essequibo river, between line 9-10 on line 9 side of riverIwokrama Reserve. Lady Smith Creek, 20.II.1995, *Mutchnick 838* (MO); Utakutu-U. Kuyuwini River, 10-15 km downstream from Taruma Rapids,27.IX.1993, *T. W. Henkel 3216* (MO). Rupununi: Kanuku mts., Bush Mouth near Witrau Falls, 10.II.1985, *Jansen-Jacobs 123* (MO); Kanuku Mts, Puwib R., near the farm of the Captain of Sandcreek, 23.II.1985, *Jansen-Jacobs 394* (MO); Kuyuwuni Landing, Kuyuwini river, 12.X.1992, *Jansen-Jacobs 3128* (MO). SURINAME. Sipariwini: Vicinity of Ulemari River, 13 km upstream from its confluence with Litani River, 02.IV.1998, *B. E. Hammer & S. Koemar 21263* (MO). COLOMBIA. Antioquía. San Luis: Cañón del Río Claro, III.1984, *Cogollo 1411* (MO). Vaupés. Mitú and vicinity; along lower Río Kuduyarí., 29.VII.1975, *Zarucchi 1675* (MO). BRAZIL. Amazonas. Barcelos: Arquipélago de Mariuá, entre as comunidades Don Pedro II e Cauburis, 20.X.2008, *A. B. Junqueira 741* (INPA) – Boa Vista: Rio Branco, 02.IX.1943, *Ducke 1355* (INPA) – Codajás: Paisagem Lago Badajoz: Margem esquerda Norte, Solimoes, 18.I.2011, *B. G. Luize 343* (INPA) – Manacapuru: Manacapuru, 14.III.1985, *F. F. Magalhaes 14* (INPA) – Manaus: Campus do INPA-Botânica-Aleixo, 16.IV.1999, *A. L. Cortez 10* (INPA); Ilha do Pombo, 08.IV.2007, *R. Mouzinho s.n.* (INPA); Parque dos Bilhares, 06.XI.2013, *A. K. F. Filatoff 1* (INPA). Mato Grosso do Sul: Ladário, 01.X,

Terminal de Bramese de Porto Ladário, rio Paraguai, 01.XII.1992, *V. J. Pott et al. 2008* (CTES). Pará: Alenquer: Río Mamiá, beira do rio, 4.III.1953, *R. L. Fróes 29452* (INPA) – Bélem: Río Xingú, foz do río Barajá, Ilha Adjacencia, 31.I.1987, *L. S. Coelho 426* (INPA) – Itatuiba: Río Tapajós, Lorena, Km 196, *M. F. da Silva 2281* (INPA) – Oriximinã: Rio Paru do Oeste, próximo do lugar Raimunda, 04.IX.1980, *C. A. C. Ferreira 2014* (INPA) – Santarém: Alter do Chão, Hotel Beloalter, maegeando ao Lago Verde à direita, 09.II.2011, *J. A. Lombardi 8001* (HUEFS) – Alter do Chão, Lago Verde, 13.VIII.2015, *T. E. Almeida 4069* (HUEFS) – Tucuturí: Margem direita do rio Tocantins, Igarapé Murixoba, 08.II.1980, *P. L. B. Lisboa 1518* (INPA). Roraima. Río Anauá, Cuadrícula NA-20-Z-D, entre ponto 3A e a foz, 30.IV.1974, *J. M. Pires 14511* (NY). São Paulo: Campinas: IAC, Fazenda Santa Elisa, Monjolinho, 30.IV.2004, *J. E. A. Bertoni & A. Geremias 1053* (IAC). ECUADOR. Guayas. Naranjal: Reserva Ecológica Manglares-Churute. Base del cerro Cimalón, 28.II.1992, *C. E. Cerón 18351* (MO). Loja. Bosque Petrificado Puyango, 31.V.1995, *X. Cornejo S. 3949* (MO). PERÚ: Amazonas. Concordanque: 400 m atrás de La Poza, río Santiago, 23.VIII.1979, *J. A. Leveau 284* (MO). Loreto. Maynas: Iquitos. Rio Momon, trib Rio Nanay;Riparian; from mouth to above San Andrés, 30.XII.1977, *S. T. Mc.Daniel 21353* (MO); Laguna de "Yarana", Río Nanay, 15 minutos arriba de Mishana (Johnson 40 hp) hacia la izquierda, 17.VIII.1978, *C. Díaz 450* (MO); Punchana. Río Nanay, Quebrada de Momoncillo, en orilla inundable, ca 30 minutos de Bellavista, 13.V.1993, *N. Rimachi Y. 10592* (MO). PARAGUAY. Central: 05.IX.1971, *A. Krapovickas & C. Cristóbal 19790* (CTES). Concepción: Colonia Yby Yahú, empalme rutas 3 y 5, 14.XII.1983, *R. Vanni Et al. 246* (CTES).ARGENTINA. Formosa: Río Paraguay, km 1416, Costa Colonia Aquino 20.III.1984, *J. J. Neiff 1623* (CTES); Río Paraguay, Villeta, 07.III.1984, *J. J. Neiff 1615* (CTES).

MUELLERA SERICEA

ECUADOR. Guayas: oad from Guayaquil to Cuevedo; km 78, 12.XI.1961, *C. H.Doodson & L. B. Thien 1279* (MO). BOLIVIA. Beni: No location specified but coordinates, 24.VIII.2006, *S. Altamirano 3466* (MO) – Trinidad: Missiones Guarayos, 1926, *E. Werdermann 2418* (MO). Santa Cruz: Ángel Sandoval: Laguna Mandioré, on the border with Brazil, 16.VII.1998, *N. Ritter 4565* (MO) – Germán Bush: Laguna Cáceras, the northern edge of the system, 19.VII.1998, *N. Ritter 4632* (MO) – Ñuflo de Chávez: Corrales , Ex-

5 , Lomerio , Miguelito, 18.XII.1995, *A. F. Fuentes 1465* (MO); Estancia San Miguelito, 200 km NE de la ciudad de Santa Cruz puesto San Ramón, 22.VII.1995, *A. F. Fuentes 971* (MO). PARAGUAY. Alto Paraguay: Puerto Casado, 20.X.1976, *T. M. Pedersen 4103* (SI). Central:Itá Enramada, IX.1971, *Schinini 3995* (SI); Trinidad, Asunción, playas del río Paraguay, X.1971, *T. Rojas 2121a* (SI). Presidente Hayes: Remansito, ca. Puente Remanso, 15.X.1994, *A. Krapovickas et al. 45636* (SI). ARGENTINA. Chaco: Primero de Mayo: 2 km de Puerto Antequera, camping al lado del club, 08.III.2000, *R. H. Fortunato et al. 6687* (BAB); Boca del Ancho, selva de inundación, 11.XI.1973, *Eskuche 2308-5* (SI); Colonia Benítez, ribera río Tragadero, cerca del puente, 13.XI.1968, *Schulz 16487* (BAB); INTA Colonia Benítez, esquina de bosque pastorial, 06.XII.1988, *R. H. Fortunato et al. 6133* (BAB); Puerto Antequera, XI.1934, *Schulz 1154* (SI) – San Fernando: Barranqueras, Paranacito, 02.II.2007, *G. Rotta 422* (CTES) – Fontana, Crece en las orillas de ríos y riachos, XII.1936, *T. Meyer 2208* (SI). Corrientes: Bella Vista: Isla del Medio, enfrente a Corrientes, 13.XI.1934, *A. Burkart 6789* (SI). Formosa: Pilcomayo: Clorinda, XII.1973, *Insfrán 1311* (SI); Estancia Bouvier, monte cercano al casco en dirección al río Paraguay, 21.XI.1991, *R. H. Fortunato et al. 2508* (BAB, SI).

3. Typical and endemic species of the Gran Chaco ecoregion (including indicators of Chaco lineage)

ACACIA CAVEN VAR. MICROCARPA

PARAGUAY. Alto Paraguay: Palmas Chicas, Chaco Paraguayo, XII.1937, *T. Rojas 7697* (SI). **Boquerón:** Muñoz, sector Pilcomayo, Chaco Paraguayo, VIII.1925, *T. Rojas 7056* (SI). Central: Estero del Ypoá, 13 km SE of Nueva Italia, S of Pindoty, 27.I.1990, *E. M. Zardini & E. Velázquez 18687* (BAB).**Cordillera:** Confluence of Río Paraguay and Río Salado, 18.XI.1989, *E. M. Zardini & U. Velázquez 16627* (BAB).**Presidente Hayes:** Bajo Puente Remanso, 20.X.1988, *F. Mereles 1625* (SI); Colonia Menno, Misión Nueva Vida, 09.XII.1977, *P. Arenas CEFAPRIN200* (SI); Río Confuso, sobre ruta, 15.X.1988, *F. Mereles 1575* (SI). **Paraguarí:** Arroyo Yuquití, 7 km E of Nueva Italia, 08.XII.1990, *E. M. Zardini & C. Velázquez 25121* (BAB); Estero del Ypoá, N part 10 km E of Nueva Italia. Arroyo Cañabé Basin, 11.XI.1991, *E. M.*

Zardini & P. Aquino 28620 (BAB). **ARGENTINA. Formosa:** Formosa: Entre Formosa y Mojón de Fierro, 07.I.1945, *Ragonese & Cozzo s.n.* (SI); Formosa, en barrancas del río Paraguay, 06.I.1945, *A. Krapovickas 934* (SI) – Pilcomayo: P.N. Pilcomayo, monte alrededor de la casa, no date,*R. Guaglianone & M. Múlgura s.n.* (SI).

ACACIA CURVIFRUCTA

PARAGUAY. Alto Paraguay: 1,5 km al S de la línea 3 y la pista de aviación, 04.XI.1992, *R. H. Fortunato et al. 3767* (BAB); Puerto Casado & Vicinity, near Ea. Guajhá, 18.X.1956, *T. M. Pedersen 4079* (MO). **Boquerón:** Colonia Menno, Loma Plata, 12.XI.1990, *R. Vanni 1944* (CTES); Pozo Colorado, 30.XI.1988, *C. Caballero 1510* (CTES). **Presidente Hayes:** 53 km S de Retiro Pozo Arias, IX.1994, *F. Mereles & R. Degen 5814* (CTES); 65 km E de Pozo Colorado, 19.XII.1987, *A. Schinini & Palacios 25873* (CTES); Between the Río Verde and Tacuara, 14.XII.1996, *E. M. Zardini & m. Vera 46105* (BAB); Estancia La Concepción. 14 km N del Casco de la Estancia, 07.IX.1995, *Degen & Mereles 3378* (CTES); Estancia Tinfunque, 23.IX.1987, *Spichiger 2139* (MO); Ruta a general Bruguez, 24.X.1995, *R. Degen 3406* (MO); Ruta a Gral. Garay, 9 km antes de Ninfa, 24.X.1995, *R. Degen & F. Mereles 3406* (CTES); Tte. Esteban Martínez y alrededores, 28.XI.2000, *F. Mereles 8310* (CTES). **ARGENTINA. Chaco:** Almirante Brown: M. Jacuboski 18, 27 km ESE de las 4 Bocas, 06.XII.1977, *Bordón 526* (CTES); Taco Pozo, 30.IX.1938, *J. N. Carmelich s.n.* (MO) – Güemes: Al N de Wichi en el abandonado pueblo de El Pintado, orillas del Teuco, 17.XI.1990, *R. H. Fortunato et al. 4308* (BAB); Miraflores, 15.XI.2006, *C. R. Salgado 505* (CTES). **Formosa:** Matacos: 6 Km al N de Ing. G. juarez, 23.XI.2000, *R. H. Fortunato et al. 6717* (BAB, ARIZ) – Pirané: 23,9 km SSO FC Gran Guardia, 31.XI.1981, *Bordón s.n.* (CTES). **Salta:** Anta: Fundo Campos del Norte. 10 km E of J.V. González, 03.III.1989, *J. Aronson 7834* (MO).

ACACIA EMILIOANA

BOLIVIA. Tarija: Puesto Las Marías, 10 km al E DE Capirenda, 25/11/1989, *C. Saravia Toledo 2219* (CTES). Santa Cruz: Cordillera, Izozog, Comunidad La Brecha , 12.VII.1998, *A. Fuentes 2505* (CTES); Cordillera, Parque Nacional Kaa-Iya del Gran Chaco, Fortín Ravelo, lagunas del paleocauce y potreros al NE del fortín, 8.II.1998, INCOMPLETO. **PARAGUAY.** Alto Paraguay: 35 km N de Teniente Montanía, en dirección a Madrejón, 10.III.2005, *R. H. Fortunato et al. 8651* (BAB, CTES); *Quintana et al. 560* (CTES)**.** **Boquerón:** Colonia Menonita, alrededores de Filadelfia, 8 km W de Fernheim, camino a Ruta Transchaco, 08/04/1996, *R. Vanni & Kurtz 3681* (CTES); EEA Colonia Neuland, 13.IX.1990, *R. Vanni et al. 2102* (CTES); Filadelfia, 10.VIII.1991, *Saravia Toledo 10046* (CTES); *Mereles 2142* (CTES). **Presidente Hayes:** *Krapovickas 45515* (CTES); *Mereles 8772* (CTES); *R. H. Fortunato 8798* (CTES).

BAUHINIA ARGENTINENSIS* VAR. *ARGENTINENSIS

BOLIVIA. Santa Cruz. Cordillera: Parque Nacional Kaa-Iya del Gran Chaco, Paleodunas 25 km al Ne de Palmar de las Islas, fr., 12.II.1998, *Fuentes & Navarro 2290* (CTES, USZ).

PARAGUAY. Alto Paraguay: Proposed Biosphere Reserve Gran Chaco Americano: Agua Dulce, *E. M. Zardini & Apestegui 58283* (BAB); Puerto Casado, XI.1916, *Rojas 2111* (SI). **Amambay:** P. N. Cerro Corá, cerrado scrub on Lorito Picada on red sandy soil, 11.XI.1999, *E. M. Zardini & P. Báez 52454* (BAB). **Boquerón:** At projecto Tagua, on the road to Fortín Toledo, 18.II.2002, *M. Luckow et al. 4496* (BAB); Between P. N. Teniente Agripino Enciso y Nueva Asunción, 27.I.1995, *E. M. Zardini & A. Acosta 42266* (BAB); Colonia Ferhneim, Colonia 22, XI.1981, *Arenas 1817* (SI); Colonia Menno, Paratodo, 01.II.1976, Fortín Gabino Mendoza, 12k km del desvío a Parque Cué sobre ruta a Lagerenza, 30.III.1975, *F. Me reles & R. Degen 3271* (CTES); Proposed National Park Médanos del Chaco, 12.XII.1998, *E. M. Zardini & N. Duarte 49276* (BAB); Ruta Transchaco, 9 km W de Mariscal Estigarribia, 07.III.1979, *Schinini & E. Bordas 1639* (CTES). **Central:** ruta Transchaco, 25 km S de Nueva Asunción, fl., 12-XII-1987, Schinini & Palacios 25671 (CTES). **Concepción:** Concepción, Mercado, 27.II.1994, *Krapovickas & Cristóbal 45077* (CTES). **Guairá**: Cerro Peró, Guairá, entre rocas desnudas, II.1924, *Rojas 4823* (SI). **Presidente Hayes:** Paratodo, fr., 9-XII1974, *Arenas 1101* (CTES). **ARGENTINA.** Formosa: Bermejo, 5 km al S de Pozo de Maza, 27.III.1999, *Scarpa 692* (SI) – Matacos: ruta 39, 23 km S de Ing. G. Juárez, en Aibal, fr., 6.III.2001,

Schinini et al. 35324 (CTES) – Patiño: Estero Patiño, Parada 102, IX.1967, *Morello s.n.* (SI). Salta. Rivadavia: Juan Solá, Estación Morillo, barrio Mataco, 10.I.1980, *Maranta et al. 149* (SI).

BAUHINIA ARGENTINENSIS* VAR. *MEGASIPHON

BOLIVIA. Chuquisaca: El Salvador-Cimboc, 27.I.1992, *Toledo & Nelson Joaquín 10436* (CTES); El Salvador-Cimboc, Laguna Seca, Campo Quemado, 09.XII.1992, *Pensiero & G. Marino 4391* (BAB). **ARGENTINA. Salta:** Güemes, Campo Santo, Finca yaquiasmé, 7.II.1957, *de la Sota 1370* (CTES) – San Martín: Dragones, 12.III.1992, *Saravia Toledo 10769* (BAB).

BAUHINIA HAGENBECKII

PARAGUAY. Central: Taumandy, 02.IV.1973, *Schinini 6187* (SI). **Cordillera:** Caacupé, XI.1947, *M. Rodríguez 14083* (SI); Tobaty, III.1972, *A. Schinini 2346* (SI); Valenzuela, s.f., *Hassler 6958* (SI) – **Concepción:** Apa, Bella Vista, s.f., *Hassler 7898* (SI). **Paraguarí:** Cerro del Acahay, II.1919, *T. Rojas 3311* (SI). **San Pedro:** Tapiracuay, 8 km de San Estanislao, camino a Rosario, 17.II.1962, *A. Krapovickas 13920* (SI).

CHAMAECRISTA ARACHIPHYLLA

BOLIVIA. Santa Cruz: Cordillera, Bañados del Izozog, 65 km al W de las instalaciones de Estancia Cachari, trayecto entre la pampa de Guanacos y Palmarito - Agua Fea. , **PARAGUAY. Alto Paraguay:** Cerro Cabrera - Picada Palmar, 22.V.1996, *F. Mereles 6390* (MO)**. Boquerón:** 1 km O de General Eugenio Garay, 08.V.1988, *Charpin & Ramella 21426* (BAB); 25 km SE de Nueva Asunción, 15.V.1994, *A. Krapovickas 45456* (BAB); Between P. N. Teniente Agripino Enciso y Nueva Asunción, 27.I.1995, *E. M. Zardini & A. Acosta 42226* (BAB). **ARGENTINA. Salta:** San Martín, 16 km al E de Dragones, 12.III.1992, *C. Saravia Toledo 10773* (G).

CHLOROLEUCON CHACOENSE

BOLIVIA. Santa Cruz: Parque Nacional Kaa-Iya del Gran Chaco, 24.V.1998, *Fuentes & J. Wirandaco 2403* (CTES);Parque Nacional Kaa-Iya del Gran Chaco, 30 km W del Campamento San José, sobre el gasoducto, V.1998, *Navarro& Fuentes 2438* (CTES)*;* Parque Nacional Kaa-Iya del Gran Chaco, 58 km W del Campamento San José, sobre el gasoducto, no date registered, *Navarro & Fuentes 2420* (CTES). **PARAGUAY. Boquerón:** 14 km N del cruce de Teniente Montanía, en dirección a Madrejón, 10.III.2005, *R. H. Fortunato et al. 8646 (BAB, CTES).*

DESMANTHUS TATUHYENSIS* VAR. *BREVIPES

PARAGUAY. Uncertain department: Pilcomayo River Basin, Río Negro, 06.III.2008, *R. H. Fortunato et al. 9177* (BAB). **ARGENTINA. Chaco:** San Martín: 14 km al N de La Eduviges, 05.XI.1983, *R. H. Fortunato et al. 201* (BAB). **Formosa:** Patiño: 20 km al O de Ibarreta por antigua Ruta 81, 12.XI.1983, *R. H. Fortunato et al. 494* (BAB); 3 km del cruce con ruta Pozo de Navagan-Lugones, hacia Estero Chumaco (Paraguay), ruta al Paraguay, 26.III.1992, *R. H. Fortunato et al. 3212* (BAB) –Pilcomayo: En sabana entre ruta 11 vieja y ruta 11 nueva, sobre el camino que une Puesto Salvación con la localidad de Bouvier, 11.XII.1967, *L A. García & Piccinini 73* (BAB); Estancia Bouvier, monte en camino de acceso en dirección al casco, 19.XI.1991, *R. H. Fortunato et al. 2450* (BAB) – Pirané: Ruta Nac. 16, a 20 km del límite con Santiago del Estero, cerca de Los Frentones, 23.XI.1990, *R. H. Fortunato et al. 1607* (BAB); Ruta 81, costado del camino, a unos 10 km de Palo Santo (hacia el E), 18.I.1981, *C. Petetín & Molina 1247* (BAB). Jujuy: Uncertain department (Capital or Palpalá): Ruta 66, 10 km SE de San Salvado de Jujuy, FECHA, *A. Schinini 22370* (F). **Santiago del Estero:** Silípica: Arraga, EEA. Santiago del Estero, 19.X.1982, *E. Carrizo 34* (BAB). **Tucumán:** Leales: Chañar Pozo, no date registered, *Venturi 429* (NY).

DENYSOPHYTUM STUCKERTII

PARAGUAY. Boquerón: Camacho, Chaco Paraguayo, III.1935, *T. Rojas 7329* (SI); General Garay, 69 km hacia Mariscal Estigarribia, 05.XI.1993, *S. Beck & M. Liberman 9443* (SI)*;* Parque Valle Natural, 8 km de Colonia Neuland, 13.III.2005, *R. H. Fortunato ET al. 8757* (BAB, SI).

ERYTHROSTEMON ARGENTINUS

BOLIVIA. Santa Cruz: Cordillera: 14 km NW de Boyuibe, 14.IV.1977, *Krapovickas et al. 31325* (SI); Camino hacia Boyuibe, entre mano derecha hacia Palmerita, no date registered, *Arroyo et al. 3249* (SI); Charagua, finca Itaguarezenda, 16.IX.1982, *Cabrera & M. M. Gutiérrez 33647* (SI); Estancia Rancho Chico, puesto nuevo, 22.V.1998, *Fuentes 2390* (SI). **ARGENTINA. Jujuy:** Santa Bárbara: Desvío de R34 a Aguas Calientes, Arroyo Aguas Calientes, 4 km E del Río San Francisco, 12.IX.1991, *R. Guaglianone et al. 2574* (SI).

ERYTHROSTEMON COLUTEIFOLIUS

Bolivia. Cordillera: Cerro Toborochi, 10–12 km por brecha abandonada al SE de Estancia Toborochi., 05.I.1991, *I. G. Vargas & G. Navarro 1922* (K). **PARAGUAY. Boquerón:** Destacamento General Diaz, 14.XII.1993, *R. Degen 3029* (MO); General Garay, 13 km hacia Mariscal Estigarribia, 05.XI.1986, *S. Beck & M. Lieberman 9442* (SI); Km 700, Transchaco Highway, VII.1976, *P. N. Brandt 17* (SI); Nueva Asunción-Eugenio Garay, 19.XI.1992, *F. Merelres 4931* (MO); Picuiba, Chaco Paraguayo, III.1935, *T. Rojas 7310* (SI);Ruta Transchaco, 11 km NW de Nueva Asunción, 12.XII.1977, *A. Schinini & R. Palacios 25717* (SI). **ARGENTINA. Chaco:** Güemes: 5 km al E de Torsalito, 22.XI.2001, *R. H. Fortunato et al. 7474* (MO). **Salta:** Güemes: ruta 34 al N Rio Juramento, 03.XI.1974, *Burkart et al. 30483* (MO); Jaquiasme, 22.XII.1938, *F. Devoto & Rial Alberti 3102* (K) –Rosario de Lerma: Campo Quijano, 17.I.1929, *S. Venturi 8477* (SI). **Santiago del Estero:** Pellegrini: Los Baños, Cerro del Remate, XII.1932, *Peirano s.n.* (SI). **Tucumán:** Capital: El Cadillal, 26.X.1927, *S. Venturi 5446* (MO) – Tapia: Tapia, no date, *Rodríguez 225* (SI). Trancas: cerca de el Aduralde en el camino a Salta, no date, *P. G. Lorentz 1004* (CORD); Entre Tapia y Vipos, a 500 m después de las casas, sobre el margen izquierdo de la ruta, 05.XII.1989, *L. Galetto 132* (MO); Tapia, Falera en los cerrillos, 12.XII.1920, *S. Venturi 1117* (MO); Vipos, 22.XII.1907, *M. Lillo 7237* (SI).

LOPHOCARPINIA ACULEATA

PARAGUAY. **Boquerón:** 23 km N de Teniente Montanía, 10.III.2005, *R. H. Fortunato ET al. 8650* (BAB, SI); 54 km al S del Parque Nac. Defensores del Chaco, 12.III.2005, *R. H. Fortunato et al. 8739* (BAB, SI) Colonia Fernheim, 10 km E de Campo Grande, 15.IX.1990, *R. Vanni ET al. 2137* (SI); Misión Nueva Vida, II.1981, *P. Arenas 1729* (SI); Tyto, Mariscal Estigarribia km 470, Ea. Ramona, 13.X.1992, *F. Mereles & Degen 4701* (SI). **ARGENTINA. Formosa:** Bermejo: Vaca Perdida, 05.XII.1996, *G. F. Scarpa 697* (SI) – Matacos: Ingeniero Juárez, ruta a Puerto Irigoyen, a 3 km al N de Puerto Irigoyen, 12.I.1957, *A. Burkart 20216* (SI).

MIMOSA CASTANOCLADA

BOLIVIA. Chuquisaca: Luis Calvo, San Isidro, 18.XII.1992, *Saravia and Nelson Joaquín 10903* (BAB). **Santa Cruz:** Cordillera: 71 km E de Boyuibe, ruta a Paraguay, 14.XII.1990, *Saravia Toledo 2862* (CTES); Parque Nacional Kaa-Iya del Gran Chaco, 20 km al W del PuestO Misiones, 19.VII.1998, *3638* (CTES); PN Kaa-Iya del Gran Chaco. Paleodunas 25 km NE de Palmar de las Islas, 12.II.1998, *Fuentes & Navarro 2277* (CTES). **PARAGUAY. Alto Paraguay:** 20 km al N del Puesto 4 de Mayo, por Línea 6, 24.X.1992, *Fortunato et al. 3621* (BAB); Cerro León Area, 13.II.1999, *Zardini & Godoy 50150* (BAB); Chaco, Línea 3 (Oeste), km 50, 09 Dec 1992, *Ramella et al. 2911* (BAB); Chaco, Palmar de las Islas, III.1989, *Mereles & Ramella 2849* (BAB, CTES); Chaco, Mayor Pedro Lagerenza, 04.VI.1978, *Schinini & Bordas 15114* (CTES); Nueva Asunción, ruta trasnschaco, 07.III.1979, *A. Schinini & E. Bordas 16416* (CTES); Parque Nacional Defensores del Chaco, Madrejón, 15.VIII.1983, *Hahn 1608* (BAB); proposed Biosphere Reserve "Gran Chaco Americano": Cerro León, 10.II.2002, *Zardini and Rivas 58521* (BAB). **Boquerón:** Nueva Asunción, ruta transchaco, 61 km W de Teniente Ochoa, 14.XII.1987, *A. Schinini & Palacios 25725* (BAB, CTES); Ruta Transchaco, km 470, 30.VI.1992, *Mereles & Degen 4507* (CTES).

MIMOSA CENTURIONIS

PARAGUAY. Concepción: Centurión, no date, *Fiebrig 4001* (G); Villa Sana, no date, *Fiebrig 4581* (G).

MIMOSA CORDOBENSIS

ARGENTINA. Córdoba. Cruz del Eje: Río Cabalabumba, Los Mogotes, 28-II-2014, *Barboza et al. 4192* (CORD). Totoral: Siena Chica (falda E): Arroyo Chilliorco entre Ascochinga y Santa Catalina, 26.X.1982, *A. T. Hunziker et al. 24372* (CORD).

MIMOSA CHACOËNSIS

Material selecto examinado. **ARGENTINA. Formosa:**Patiño:Muchutti, 10 km Norte de Las Lomitas, 4.XII.1978, *Bordón s.n.* (CTES 5719). **BOLIVIA.Chuquisaca.** Carlos Calvo:El Salvador-Cimboc, Laguna El Panta, 21.I.1992, *Saravia Toledo & Nelson 10312* (BAB); El Salvador, 8.XII.1992, *Pensiero & Marino 4379* (NY, SI). **Santa Cruz.**Cordillera:Estancia Perforación y alrededores, XI.2001, *Fuentes 3384* (MO). **PARAGUAY. Alto Paraguay.**A 200 m de la delegación de Nueva Asunción, 18-XI-1992, *Degen 2899* (FCQ, MO); Palmar de las Islas, 12.III.1989, *Mereles 2849* (FCQ); Nueva Asunción, 60 km al Oeste de Estancia La Patria, 7.XII.1992, *Nicora et al.* 9753 (BAB); ruta Transchaco, 25 km Sur de Nueva Asunción, 12.XII.1987, *Schinini & Palacios 25664* (BAB, FCQ, MO).**Boquerón.** Between Parque Nacional Teniente Agripino Enciso y Nueva Asunción, 27.I.1995, *Zardini & Acosta 42248* (BAB); proposed National Park Médanos del Chaco, 13.XII.1998, *Zardini & Duarte 49715* (BAB); 12.XII.1998, *Zardini & Duarte 49672*, 49596 (BAB).

MIMOSA MORONGII

PARAGUAY. Central: On the Gran Campo near Luque, no date, *T. Morong 728* (NY);Puesto de Peaje Emboscada, ca. Del río Salado, 06.III.2008, *R. H. Fortunato et al. 9187* (BAB, SI). **Paraguarí:** Ad ripas lagunae Yparacay, september, *Hassler 1035* (G, NY).

MIMOSA DETINENS

PARAGUAY . Boquerón: 3 km NE of Campo Loa Projecto Tagua on the road to Fortín Toledo, 17 Feb 2002, *Luckow et al. 4491* (BAB); Filadelfia, 06 Dec 1983, *Hahn 1856* (BAB); Parque Nacional Teniente

Agripino Enciso, 14 Dec 1998, *E. M. Zardini and N. Duarte 49916* (BAB). **ARGENTINA. Catamarca:** La Paz: Las Tejas, 08.II.1947, *A. Brizuela 763* (CTES). **Chaco:** Doce de Octubre: Zona Gobernador Pinedo 08.VIII.1962, *A. G. Schulz 11275* (CTES); Gancedo, 03.I.1975, *A. Krapovickas 27167* (CTES) – General Güemes: Castelli, 21.III.2005, *C. R. Salgado 447* (CTES); De Nueva Pompeya al Pago de Suri, 14.III.1970, *A. G. Schulz 17457* (CTES); Picada Canal Bermejo, km O de la Picada, 24 km al O de J. J. Castelli, 27.VII.1966, *A. G. Schulz 15508* (CTES) – Maipú: Tres Isletas, al S de Tres Isletas, antes de llegar, 18.III.1952, *A. G. Schulz 8292* (CTES) – O'Higgins: La Gran Bestia", II Sección, 1-2.XI.1954, *L. A. Duarte 461* (CTES). **Salta:** Anta: Joaquín V. González, no date registered, *M. R. Álvarez 218* (CTES); 54 km NE de Joaquín V. González, camino a Punta Blanca, no date registered, *A. Krapovickas & C. Cristóbal 46282* (CTES); Ruta 16, 7 km SE de Quebrachal, 08.XII.1979, *A. Schinini 19510* (CTES). **Santiago del Estero:** Moreno: Tintina 15 km, 12.II.1984, *KPDT 36* (CTES); Robles: Colonia Jaimez, 15.XI.1948, *F. E. Luna 342* (CTES).

MIMOSA TOBATIENSIS

BOLIVIA. Santa Cruz: Ñuflo de Chávez: 10 km W of Quimome, no date, *F. Mamami & Saucedo 676* (K). **PARAGUAY. Cordillera:** Cerca del peaje Emboscada, 06.III.2008, *R. H. Fortunato ET al. 9177* (BAB); entre Bernalcue y Emboscada, in regione lacus Ypacaray, II.1913, *E. Hassler 12501* (G); Tobatí, 09.I.1951, fl, *Sparre & Vervoorst 1504* (Paratipo: BAB).

MIMOZYGANTHUS CARINATUS

BOLIVIA. Chuquisaca: Luis Calvo, 11.XI.1993, *Saravia Toledo 12045* (CTES). **PARAGUAY. Boquerón:** no date, *M. R. Verena 21* (CTES); II.1981, *P. Arenas s.n.* (CTES); *P. Arenas 3299* (CTES); 12.II.2004, *P. Arenas 3801* (CTES). **Presidente Hayes:** 28.XI.2000, *Mereles et al. 8296* (CTES). **ARGENTINA. Formosa:** Bermejo, no date registered, *P. Arenas 3013* (CTES); Matacos, XI.1969, *M. I. Torres 58* (CTES). **Jujuy:** San Pedro de Jujuy, 04.XI.1971, *Marmel et al. 8677C* (CTES). **Salta:** Rivadavia: 17.II.2005, *M. E. Suárez 43* (CTES).

PIPTADENIOPSIS LOMENTIFERA

BOLIVIA. Chuquisaca: Luis Calvo, no date, *C. Saravia Toledo 10154* (CTES). **Santa Cruz:** Cordillera, 13.XII.1983, *C. Saravia Toledo 2683* (CTES); 12.IV.1993, *C. Saravia Toledo 11755* (CTES). **PARAGUAY. Alto Paraguay:** Chaco, P. N. Defensores del Chaco, Madrejón, 15.VIII.1983, *Hahn 1619* (CTES); Sector Puerto Casado, X.1938, *T. Rojas 8457*[a] (SI); Teniente Montanía, km 10, E, 19.X.1992, *F. Mereles & R. Degen 4795* (SI). Boquerón: Isla Poí, 200 km ESE de Puerto Casado, VIII.1934, *T. Rojas 7033* (SI). **Presidente Hayes:** Colonia Menno, Misión Nueva Vida, 02.II.1976, *P. Arenas 1514* (SI).

PROSOPIS ELATA

BOLIVIA. Santa Cruz: Cordillera; Parque Nacional Kaa Iya del Gran Chaco, Palmar de las Islas, 11.II.1998, *Fuentes & Navarro 2240* (CTES). **PARAGUAY. Alto Paraguay:** 30 km de Fortín Lagererenza, hacia Lagerenza-í, 11.X.1988, *M. Quintana 668* (CTES);Agua Dulce, 05.X.1979, *A. Schinini 18134* (CTES). **Boquerón:** Aproximadamente 10 km E de Mcal. Estigarribia, 15.XII.1987, *R. A. Palacios 1502* (CTES); Cnia. Fernheim. Colonia 22, II.1981, *P. Arenas 1797* (CTES); Filadelfia, 07.X.1979, *A. Schinini & E. Bordas 18193* (CTES); Fortín Teniente Ochoca, 11.XII.1987, *R. Palacios 1488* (CTES); Nueva Asunción, Fortín Teniente Ochoa, 11.XII.1987, *R. Palacios 1481* (CTES); Paratodo, 08.XII.1974, *P. Arenas 1091* (CTES); Tyto. Fortín Nueva Asunción, Tte. Enciso, 17.XI.1992, *R. Degen & F. Mereles 2882* (CTES). **ARGENTINA. Salta: Anta:** 54 km NE de Joaquín V. González, camino a Punta Blanca, 22.XI.1984, *A. Krapovickas 46280* (CTES); "Los Colorados", 110 km NNE de J. V. González, 21.XII.1986, *C. Saravia Toledo 12681* (CTES) – Rivadavia: coronel Juan Solá (Morillo), 19.II.2005, *M. E. Suárez 80* (CTES); Misión La Paz (sobre río Pilcomayo), 02.I.2003, *G. F. Scopa 541* (CTES); ruta 81, 21 km SE de Cnel. Juan Solá (Morillo), 10.XII.1979, *A. Schinini 15975* (CTES); ruta 81, 35 km SE de Coronel Solá (Morillo), 10.XII.1979, *A. Schinini 19580* (CTES). **Santiago Del ESTERO:** Copo: Entre Monte Quemado y Los Tigres, 24.I.1949, *Ragonese & Castiglioni 7191* (CTES).

PROSOPIS FIEBRIGII

PARAGUAY. Alto Paraguay: Palmas Chicas, Chaco Paraguayo, XII.1937, *T. Rojas 7227* (SI);Puerto Casado, Chaco Paraguayo, IX.1938, *T. Rojas 8229* (SI). **ARGENTINA. Formosa:** Patiño: El Cogoik, 07.XII.1972, *R. Palacios 484* (SI); Fortín Pilcomayo, 16.I.1945, *A. Krapovickas 1207* (SI).

PROSOPIS KUNTZEI

BOLIVIA. Santa Cruz: Florida: Santa Cruz, 166 km hacia Cochabamba, 03.X.1981, *S. Beck 7165* (CTES); Vallegrande: El Bello, 14.8 km de Vallegrande, camino a Moro-Moro, 19.XII.1993, *Saravia Toledo 12082* (CTES). **PARAGUAY. Boquerón:** Camino entre Teniente Montanía y Mcal. Estigarribia, 06.II.2005, *C. Vogt & F. Mereles 211* (CTES); Colonia Fernheim, colonia 22, II.1981, *P. Arenas 1794* (CTES); Colonia Menno, Fehr, 68 km NE de Filadelfia, 10.XII.1992, *A. Krapovickas 44292* (CTES); Estación de Servicio, Loma Plata, 09.III.2005, *R. H. Fortunato ET al. 8656* (CTES); Estancia Laguna de Cristal, 12.IV.2003, 12.IV.2003, *F. R. Verena 30* (CTES); Filadelfia, 13.III.1979, *A. Schinini & E. Bordas 16570* (CTES). **Presidente Hayes:** Ruta a Gral. Díaz, DEsvío a Colonia Nivaclé, 24.IX.1993, *F. Mereles & R. Degen 5404* (CTES); Santa Asunción, 12.II.2004, *Egea & Peña 248* (CTES). **ARGENTINA. Corrientes:** Sauce: 36 km N de Sauce, 12.XI.1981, *A. Schinini* 21659 (CTES). **Chaco:** Chacabuco : Charata, 04.XI.1994, *M. Alvarez 72* (CTES). Comandante Fernández: 20 km NE de Comandante Fernández, ruta 95, 19.II.1985, *Schinini 19991* (CTES); Napenay, RN16, 29.XII.1974, *Krapovickas 17296* (CTES) – General Güemes : 2 km OSO Comandancia Frías (picada), 15.X.1972, *C. Bordón s.n.* (CTES) ; Camino de Fuerte Espranza a Taco Pozo, 30.X.1986, *A. Schinini & M. Pires 24917* (CTES) ; El Pintado, RP9, orillas del río Teuco, 21.I.1980,*Piccinini & Hilfer 4177* (CTES) ; Miraflores, 14.IX.2006, *C. R. Salgado 449* (CTES) ; Paraje Costa Rica, reserva ecológica APROMA, 5 km E de 4 de febrero, cauce del Arroyo Guaycurú, 27.XI.1996, *A. Krapovickas 46851* (CTES). – Doce de Octubre: 4 km W de Itín, 21.IX.1972, *A. O. Bordón s.n.* (CTES) ; Gobernador Pinedo, 15.III.1937, *A. G. Schulz 8937* (CTES). – Donovan: Alrededores del zoológico de Sáenz Peña, 15.X.2014, *W. Medina 815* (CTES) - Primero de Mayo: Margarita Belén (ilegible), 30.XI.1943, *A. G. Schulz 4074* (CTES). – 25 DE Mayo : 9 km S de Machagai, 19.II.1980, *A. Schinini 20016* (CTES) – o'Higgins: 2 Sección, La Gran Bestia, XI.1954, *M. Duarte 471* (CTES). **Formosa:** Bermejo: Laguna Yema, 8 km S de EE Laguna Yema, 23.IV.1999, *R. Vanni ET al. 4338* (CTES) **–** Laishi: Reserva El Bagual, 15.X.2001, *A. Di Giacomo 537* (CTES) – Matacos: 30 km W de ing. JUÁREZ, 22.IX.1997, *G. J.*

Seijo & DeMatteis 1149 (CTES) – Patiño: Estancia El Bellaco, 15.VIII.1972, *Schulz 18309* (CTES); Ruta 81, 3 km NW de Bazán, 3 Pozos , 11.XII.1984, *A. Schinini 24240* (CTES) – Pirané: Ruta Nacional 90, 20 km al S de Pirané, 06.X.1981, *Valla 213* (CTES). **Salta:** Rivadavia: R81, 21 km SE de Cnel. Juan Solá (Morillo), 10.XII.1979, *Schinini 19571* (CTES). **Santiago del Estero:** Copo: Los Tigres a Monte Quemado, I.1990, *A. Del Castillo 1156* (CTES); Pampa de los Guanacos, 01.II.1974, *T. M. Pedersen 10730* (CTES) – Moreno: Entre Amamá y Tintina, 12.XI.1974, *KDTP 24* (CTES). **Tucumán:** Leales: 3 km antes de Estación Tacanas, 06.III.1966, *Vaca & Villa Carenzo 2806* (CTES).

PROSOPIS NIGRA* VAR. *RAGONESEI

ARGENTINA. Chaco: Güemes: 99 km de Fuerte Esperanza hacia J. J. Castelli, 15.XII.1983,no date, *no collector registered* (SI). **Santa Fe:** San Justo: Videla, *Ragonese* Herb. Santa Fe 2078 (SI); Vera: Vera, no date, *Herb. Inst. S. Fe 2420* (SI).

PROSOPIS NIGRA* VAR. *LONGISPINA

Chaco. Primero de Mayo: Colonia Benítez, not far from Resistencia, *A. G. Schulz 1356* (SI). **Corrientes:** Capital, 23.IX.1954, *T. M.Pedersen 2808* (SI).**Santiago del Estero:** Ojo de Agua: Ruta 9, 25 km de Ojo de Agua al N, 14.IX.1986, *R. H. Fortunato et al. 3955* (BAB).

PROSOPIS NUDA

BOLIVIA. Santa Cruz: Cordillera, Parque Nacional Kaa-Iya del Gran Chaco, ANMI Norte. 58 km al O del campamento San José, sobre el gasoducto. , 13.VI.1998, *A. F. Fuentes & G. Navarro 2409* (MO, USZ); Curuyuqui, 50 km SE of Santa Cruz on Río Parapetí, upland chaco, 24.X.1991, *A. H. Gentry ET al. 75174* (LPB, MO). **PARAGUAY. Boquerón:** 10 km S de la Reserva Biosfera Chaco Paraguayo, en ruta Montanía Madrejón, 10.III.2005, *R. H. Fortunato et al. 8654* (BAB, CTES); Chaco Boreal, Misión Santa Rosa. Manxuj Ethnobotanical Study. Vicinity of Santa Rosa in upland forest, 20.XII.1993, *T. L. Gragson 290* (MO); Estancia Laguna de Cristal, 12.II.2005, *Vogt & Mereles 257* (CTES); Estancia Rancho Silvante Newland, 01.I.2004, *F. R. Verena 67* (CTES); Estancia Tinfunque, 20.XII.1989, *F. Mereles 3329* (FCQ);

Mariscal Estigarribia, 16.IV.1978, *A. Schinini 15222* (MO); Mariscal Estigarribia, alrededores, 07.X.1979, *SChinini & Bordas 18151* (CTES).

PROSOPIS PUGIONATA

ARGENTINA. Córdoba: Uncertain department (beween Tulumba and Ischilín): Entre Quilino y San José, 07.XII.1949, *Ragonese 6599* (BAB) – Sobremonte: Entre km 907 y Mansilla, 14.X.1950, *Ragonese & Caso s.n.* (BAB74578, CTES) – Tulumba: Camino entre La Majadilla y La Esperanza, al pie de la ladera occidental de la Sierra Chica Norte, XI.1951, *M. Sayago 1685* (CTES); Entre San José y Las Cañas, 06.XII.1949, *Ragonese 6607* (BAB); N of Quilino, Ruta 60 km 880, 30.IX.1972, *B. Timmerman*s.n. (TEX)**.** **La Rioja:** Chamical: Pasando salinas, La Antigua", en el campo denominado "Loma del Tigre", 04.X.1979, *F. Biurrun 2479* (CTES) – Capital: RP25, entre Chamical y La Rioja, a unos 60 km de la primera, Campo La Chilca, 07.II.1988, *F. Biurrun & L. Blanco 5413* (CTES) – Rosario Vera Peñaloza: De Chepes hacia San Juan, no date, *A. T. Hunziker 12255* (SI).

PROSOPIS ROJASIANA

PARAGUAY. Boquerón: Filadelfia, 13.III.1979, *A. Schinini & E. Bordas 16371* (CTES); Mariscal Estigarribia, 07.I.1979, *A. Schinini & E. Bordas 18152* (CTES); Sector López de Filipis, no date, *T. Rojas 8310* (SI); Sector Pilcomayo, Magariños, *T. Rojas 8314* (SI). **Alto Paraguay:** Puerto Casado, *Pedersen 4171* (SI).

PROSOPIS RUBRIFLORA

BRAZIL. Mato Grosso do Sul: Porto Murtinho, Porto Murtinho, 20-30 km L, 24.X.1987, *G. Hatschbach 51648* (MO). **PARAGUAY. Concepción:** A 10 km de Estancia San Luis de l Sierra hacia Estancia Reyes Cué, 25.II.1990, *R. Palacios 1986* (MO); Puerto Risso, 15.I.1955, *A. G. Schulz 8841* (CTES).

SENNA CHACOENSIS

ARGENTINA. Chaco: Almirante Brown: Taco Pozo, XII.1935, *T. Meyer 2037* (SI) – Nueve de Julio: Las Breñas, 28.X.1959, *A. G. Schulz 10148* (SI).**Córdoba:** Río Seco: Colonia Candelaria, 22.I.1949, *M. Sayago*

386B (SI). **Salta:** Anta: 40 km SE de J. V. González , 17.XII.1986, *C. Saravia Toledo 1264* (SI) – San Carlos: 20 km N de Cafayate, 20.XII.1960, *A. Burkart 22053* (SI). **Santiago del Estero:** Pellegrini:Estancia El Remate 22.II.1928, *S. Venturi 5831* (SI) – Taboada: Añatuya, 27.XII.1973, *R. Palacios & Branco 509* (SI). **Tucumán:** Cruz Alta: Estación Aráoz, FFCCA, 21.XI.1925, *Schreiter s.n.* (SI).

SENNA CHLOROCLADA

BOLIVIA. Chuquisaca: Carandaity, III.1935, *T. Rojas 7200* (SI). **Santa Cruz:** Cordillera: 34 km N de Boyuibe, 29.III.2006, *DeMatteis ET al. 1976* (SI); Alto Parapetí, 24.XII.1981, *S. Beck 92* (SI); Camiri, 70 km hacia Yacuiba, 03.X.1983, *S. Beck & Lieberman 9389* (SI). **Tarija:** Villamontes, Río Pilcomayo, 30.IX.1921, *Pflantz 947* (SI). **PARAGUAY. Boquerón:** 14 km al N del cruce de Teniente Montanía, 10.III.2005, *R. H. Fortunato ET al. 8649* (BAB, SI); 14 km E de Filadelfia, Colonia Fernheim, Colonia 8 (Campo Grande), 11.IX.1990, *R. Vanni ET al. 1880* (SI); 17 km N de Filadelfia en dirección a Montanía, *R. H. Fortunato ET al. 8637* (BAB, SI); 52 km al N Del Cruce de Teniente Montanía, 10.III.2005, *R. H. Fortunato ET al. 8652* (BAB, SI); Capitán Demattei, III.1938, *T. Rojas 8436* (SI); EEA Neuland, 13.IX.1990, *R. Vanni et al. 2101* (SI); Platanillos, VIII.1934, *T. Rojas 7015* (SI); Teniente Montanía, 10.VI.1986, *F. Mereles 662* (SI). **Presidente Hayes:** Km 220, López de Filippis, orilla de monte, X.1938, *T. Rojas 8436* (SI); Nanawa, XI.1927, *T. Rojas 5225* (SI). **ARGENTINA. Formosa:** Bermejo: Puesto Desmonte al NO de Nuevo Pilcomayo, 12.XI.1941, *J. R. Cordini 64* (SI) – Matacos: Ing. Juárez, 08.IX.1978, *R. A. Palacios 5722* (SI). **Salta:** Anta: Pizarro, 09.II.1945, *A. Krapovickas 1699* (SI) – Metán: El Tunal, 21.VIII.1953, *T. Meyer 18180* (SI) – Rivadavia: Coronel Juan Solá, Morillo, 17.II.2005, *M. E. Suárez 6* (SI).

ADESMIA CORDOBENSIS

ARGENTINA. Catamarca: Ancasti: Sierra de Ancasti, entre El Portezuelo y Los Morteros, 20.XII.1963, *Ragonese 9766* (SI). Andalgalá: Andalgalá, 05.IX.1945, *P. Jörgensen 963* (SI); Alto de Las Juntas, las estancias, 12.I.1952, *H. Sleumer 2186* (SI); Ruta Prov. 48, Cuesta de la Chica, 23.III.2013, *F. Zuloaga et al. 14606* (SI); Cuesta de la Chilca, 30.I.2008, *G. Barboza et al. 1172* (SI) – Belén: Barranca Larga, II.1937, *Schreiter s.n.* (SI) – Paclín: Cuesta del Totoral, 26.XI.1960, *Ruiz Leal 21175* (SI). **Córdoba:** Uncertain

department (Colón-Punilla): Sierra Chica, camino de Ascochinga a La Cumbre, 20.XI.1936, *Nicora 1297* (SI) – Uncertain department (Pocho-Cruz del Eje): Entre Los Gigantes y Taninga, 05.XII.1958, *A. Burkart 20795* (SI) – Calamuchita: La Cumbrecita, 18-27.III.1955, *F. A. Roig 1011* (SI); Villa Reartes, 1917-1919, *Castellanos 96* (SI) – Colón: Dumesnil, Sierras, 10.II.1955, *A. ER. Lanfranchi 1251* (SI); La Calera, 23.I.1958, *A. Lanfranchi 1479* (SI); Salsipuedes, III.1938, *G. Dawson s.n.* (SI7310); Sierra Chica, Estancia La Reducción, 28.XII.1935, *A. Burkart 7400* (SI) – Pocho: Taninga, 14.X.1978, *A. L. Cabrera et al. 29651* (SI); Los Túneles, 06.XII.1958, *Ruiz Leal & F. A. Roig 20108* (SI) – Punilla: Capilla del Monte, Huertas Malas, 28.I.1941, *E. Nicora 2955* (SI); Cruz Grande, al N de La Cumbre, 28.II.1957, *A. Burkart 20442* (SI); El Batán, I.1952, *M. Sayago 819* (SI); San Esteban, 03.I.1938, *E. Nicora 1532* (SI) – Río Cuarto: Ruta Prov. 1, 3 km W de Achiras, 25.XI.1998, *A. Krapovickas 47553* (SI) – Santa María: Alta Gracia, 12.I.1940, *A. Burkart 10299* (SI) – Totoral: San Jorge, Pedanía Río Quinto, I.1950, *M. Sayago 523B* (SI). **Salta:** La Poma: Ruta 40, entre Pueblo Viejo y El Rodeo, 25.II.1987, *E. Nicora 9158* (SI). **San Luis:** General Pedernera: El Morro, 03.XI.1958, *Ruiz Leal 19870* (SI) – Pringles: Camino a La Florida desde Saladillo, 14.III.2011, *Deginani et al. 2123* (SI) – San Martín: Camino entre Villa Praga y Guanaco Pampa, 03.II.1971, *Anderson 2092* (SI).

AESCHYNOMENE PARAGUAYENSIS

PARAGUAY. Paraguarí: Chololó, 14.II.1969, *T. M. Pedersen 9301* (SI).

ARACHIS CORRENTINA

PARAGUAY. Central: Cerro Peró, entre Ypacaraí y Pirayú, 10.II.1966, *A. Krapovickas et al. 12593* (CTES). **Presidente Hayes:** Villa Hayes, 4 km W del río Paraguay, 13.XII.1972, *A. Schinini 19616* (CTES); Ruta Transchaco, entre B. Aceval y Villa Hayes, 18.V.1981, *A. Krapovickas et al. 37459* (CTES). **ARGENTINA. Corrientes:** Capital: 1 km W de Laguna Brava, 28.XII.1976, *A. Krapovickas et al. 30048* (CTES); 4 km E de R12, camino a Laguna Brava, 14.I.1966, *A. Krapovickas et al. 11919* (CTES); alrededores de la Escuela de Agricultura, 06.IX.1972, *A. G. Schulz 11791* (SI); Matadero Arroyo Pirayuí, 08.I.1966, *A. Krapovickas et al. 11905* (CTES); Ruta 12, 4 km W del aeropuerto Camba Punta, 28.XII.1976, *A. Krapovickas et al. 30050* (CTES) – Concepción: 6 km E de Santa Rosa, Ea. Millán, 27.III.1975, *M. M.*

Arbo 839 (CTES); El Batel, paso Crucecita, 11.Ii.1968, *A. Krapovickas et al. 13800* (CTES); Ruta 17, 9 km NE de Santa Rosa, 30.III.1974, *A. Krapovickas et al. 24580* (CTES); Tabay, 07.X.1973, *A. Schinini 7453* (CTES) – Itatí: A 5 km de Yacaré, Ruta 12, 23.10.1957, *M. Sayago 2133* (SI) – Ituzaingó: Ituzaingó, I.1947, *Spegazzini 10071* (SI) – Ituzaingó: 30 km W de Ituzaingó, 16.I.1970, *A. Krapovickas et al. 15680* (CTES); Estancia San Pedro,10.XI.1976, *M. M. Arbo 1088* (CTES); Ruta 12 km 1134, entre Itá Ibaté e Ituzxaingó, 05.III.1953, *A. Krapovickas 7890* (CTES); Villa Olivari, 18 km W de Ituzaingó, 02.X.1978, *A. Schinini 15669* (CTES); Lavalle: Punta del Rubio, no date, *Anzoátegui 1210* (CTES) – Mburucuyá: 10 km W de Mburucuyá, 05.II.1968, *A. Krapovickas 13782* (CTES); 32 km E de Saladas, Ea. Pindapoy, 26.XI.1970, *R. Carnevali 2271* (CTES) – San Cosme: Ramada Paso, 10.X.1975, *E. M. Zardini 644* (SI) – San Luis del Palmar: Arroyo Riachuelo, 7 km SE de San Luis del Palmar, 14.I.1966, *A. Krapovickas 11941* (CTES); between San Cosme y Ramada Paso, 09.XII.1947, *Stephens 16* (CTES); cercanías a San Luis del Palmar, 28.X.1969, *A. Burkart 27695* (SI) – San Miguel: Loreto, 25.I.1959, *Gregory et al. 9557* (CTES).

ARACHIS DURANENSIS

BOLIVIA. Chuquisaca: Tarija: Gran Chaco: 14 km N de Caparari, 08.IV.1977, *A. Krapovickas et al. 30072* (CTES); 18 km N de Yacuiba, camino a Villamontes, arroyo el Coloradito del Palmar, 07.IV.1977, *A. Krapovickas et al. 30070* (CTES); 30 km de Yacuiba, campo de La Tapia, 07.IV.1977, *A. Krapovickas et al. 30069* (CTES); 32 km N de Yacuiba, 10.IV.1977, *A. Krapovickas et al. 10073* (CTES); Simbolar del Carmen, 20 km E de Villamontes, 28.V.1971, *A. Krapovickas et al. 19408* (CTES, LPB). Tatarenda, 25.III.1902, *Fries 1465* (G). **ARGENTINA. Jujuy:** Capital: 2 km S de Palpalá, 29.III.1977, *A. Krapovickas ET al. 30061* (CTES); 8,6 km SE de San Salvador de Jujuy, 14.III.1982, *A. Schinini 21767* (CTES) – El Carmen: Perico, 1 km N de Río Perico, 28.III.1977, *A. Krapovickas ET al. 30060* (CTES, SI); Rotonda de acceso a Aeropuerto El Cadillal, 20.I.2002, *G. J. Seijo et al. 2772* (SI) – Ledesma: Ruta 34, 4 km S de Fraile Pintado, 09.XII.1970, *A. Schinini et al. 19539* (CTES). **Salta:** Anta: 1 km S de Anta, 21.III.1982, *A. Schinini 21770* (CTES); Palermo, río del Valle, 23.III.1980, *Vorano s.n.* (CTES); río del Valle, 24.I.2002, *G. J. Seijo et al. 2933* (SI); río del Valle 5 km N de Las Lajitas, 24.IV.1983, *A. Krapovickas et al. 38900* (CTES); Ruta 5 y puente sobre Arroyo Cabeza de Anta, 24.I.2002, *G. J. Seijo et al. 2825* (SI) –Capital: El Prado, río Arenales, 07.V.1959, *Gregory et al. 10038* (CTES); Salta, 07.III.1905,

Spegazzini (BAB13765); San Luis, río Arenales, 10.V.1975, *A. Krapovickas et al. 28458* (CTES) – Chicoana: Los Los, no date, *Filipovich 415* (LIL) – La Caldera: Estancia La Despensa, a 25 km de La Caldera, no date, *A. T. Hunziker 1635* (SI) – Metán: 14 km WNW El Tunal, 04.IV.1980, *Krapovickas et al. 36002* (CTES, SI); El Tunal, dique El Tunal, desvío a 3 km del dique entrada a propiedad privada, cerca del murallón, 24.I.2002, *G. J. Seijo et al. 2822* (SI) – San Martín: 16 km W de Ballivián, en arroyo afluente de cauce seco, 04.IV.1977, *A. Krapovickas et al. 30068* (CTES); 3 km E de Senda Hachada, río Seco, 02.IV.1977, *A. Krapovickas et al. 30065* (CTES); 5 km W de Dragones, 02.IV.1977, *A. Krapovickas et al. 30066* (CTES); Campo Durán, 22.I.1930, *Horovitz 5580* (SI); Ruta 34, km 1374, cruzando el puente sobre el Río Seco, margen S, 16.I.2002, *G. J. Seijo et al. 2741* (SI).

ARACHIS HASSLERI

PARAGUAY. Boquerón: Palmas Chicas, XII.1937, *T. Rojas 7623* (CTES, SI). **Concepción :** Na periferia da cidade de Loreto, 02.II.1997, *G. P. Silva ET al. 3818* (CTES); road from Concepción to Loreto, 20.V.2002, *M. J. Williams ET al. 950* (CTES).

ARACHIS LIGNOSA

PARAGUAY. Concepción: Concepción, *T. Meyer 18729* (LIL); Puerto Fonciere, 18.II.1948, *Stephens et al. 178* (LIL). **Paraguarí:** Paraguary, XII, *Hassler 6515* (G). **Presidente Hayes:** Puerto Militar, puente sobre el río Paraguay, frente a Concepción, 10.XII.1989, *Vanni et al. 1291* (CTES).

ARACHIS MICROSPERMA

BRAZIL. Mato Grosso do Sul: Bela Vista: Bela Vista, junto a alfándega de Bela Vista em gramados ornamentais, entrada a Bela Vista desde Jardim, área urbanizada junto a margem do río Apa, *Valls ET al. 7681* (CTES); entra a Bela Vista desde Jardim, 24.IV.1985, *Valls ET al. 8711* (CTES).

APURIMACIA DOLICHOCARPA

ARGENTINA. Córdoba: Pocho: Rp21, DE Tanti a Taninga, 08.X.2009, *F. Zuloaga ET al. 11318* (SI) – Cruz del Eje: Los Gigantes, 02.II.1952, *M. Sayago 267* (SI) – Pocho: Sierra de Achala, Abra de viaje más

allá de Las Chacras en el camino entre Salsacate y Los Gigantes, 28.X.1949, *A. T. Hunziker no registered* (SI); Sierra Grande, falta occidental, en Cuesta de Las Chcras, entre Arroyo Murúa y Arroyo Albarracín, 03.II.1951, *A. T. Hunziker 8838* (SI).

DALEA ELEGANS* VAR. *ELEGANS

BOLIVIA. Tarija: Cercado, ca. Tablada, 09.III.1986, *E. Bastián 990* (SI). **ARGENTINA. Córdoba:** Uncertain department (Colón-punilla): Camino de ASCOCHINGA A La Cumbre, 20.XI.1936, *M. L. Giardelli s.n.* (SI) – Calamuchita: Cumbrecita, 17.II.1953, *A. Krapovickas 7595* (SI); Villa Reartes, 1917-1919, *Castellanos 6891* (SI); Entre El Sauce y Minas de Wolfram, III.1894, *W. Bodebender 8187* (SI) – Colón:Salsipuedes – Ischilín: Los Terrones de Ongamira, 14.XII.1969, *R. Cute s.n.* (SI) – Jesús María: Estancia La Reducción, 26.XII.1935, *A. Burkart 7350* (SI) – Punilla: Campo San Roque, 12.II.1863, *J. Isern 8142* (SI); Capilla del Monte, III.1924, *M. Barros 763* (SI); Carlos Paz, 31.XII.1935, *A. Burkart 7363* (SI); Copina, 13.I.1940, *A. Burkart 10305* (SI); entre Quebrada de las Higueras y San Esteban, 11.XII.1885, *F. Kurtz 2836* (SI); Huerta Mala, 28.I.1941, *E. Nicora s.n.* (SI18544); La Falda, El Cuadrado, 27.XI.1986, *R. Kiesling & O. Ferrari 6417* (SI); near Capilla del Monte, path up Cerro Uritorco, 23.I.1966, *J. H. Hawckes et al. 3288* (SI); Sierra chica, Cerro Uritorco, falda Oeste, 15.I.1965, *A. T. Hunziker 17979* (SI); Villa Allende, XI.1924, *M. Barros 760* (SI) – Santa María: Malagueño, 24.I.1899, *T. Stuckert s.n.* (SI); Sierra Chica, Ochoa, en falda de cerro, 11.III.1951, *A. T. Hunziker 8971* (SI) – Totoral: San Jorge, I.1950, *M. Sayago 497* (SI) – Tulumba: Camino entre Deán Funes y San Pedro Norte, 14.II.1962, *A. Coccucci 375* (SI). **Jujuy:** Tumbaya: Volcán Chilcayo, camino a Abra Morada, 26.II.1985, *R. Kiesling et al. 5804* (SI). **San Luis:** Junín: Sierra de Comechingones, subiendo la falda frente a Estancia El Rincón, 08.II.1956, *A. T. Hunziker 11774* (SI) – Pringles: Arroyo, 500 m antes del Cerro Sololosta, 20.I.1956, *F. A. Rojas 6* (SI); Camino a Virorco, cerca del primer cruce, pastizal serrano, 14.I.1971, *D. L. Anderson 1970* (SI); El Durazno, I.1934, *F. A. Pastore 6622* (SI); Intihuasi, 05.II.1936, *Vignati 24* (SI).

CHAETOCALYX CHACOËNSIS

BOLIVIA. Chuquisaca: El Salvador Cimboc, La Pista, 20.III.1992, *Joaquin s.n* . (CTES). **Santa Cruz:** Cordillera, Parque Nacional Kaa-Iya, del Gran-chaco Paleodunas, 25 Km al NE de Palmar de las Islas,

12.II.1998, *Fuentes 2383*(CTES, USZ). **PARAGUAY. Alto Paraguay:** Parque Nacional Defensores del Chaco. Tyto a Palmar de Las Islas, 20.VII.1997, *F. Mereles 6672* (CTES). **Boquerón:** Colonias Mennonitas, ruta Trans-Chaco, 27-VI-1995, Utzschneider 16 (CTES); de Picuiba a Irindagué, III.1935, *T. Rojas 7398* (SI); Picada Jojoba Motor, Km 10 ruta TransChaco, 8.XII.1992, Mereles et al. 4971 (CTES, FCQ); Ruta Trans Chaco, 35 Km SE de Nueva Asunción, 12.XII.1967, *Schinini & Palacios 25722* (G);. In nemore 4-6 Km antecastrum Sargento Rodríguez, 10.III.1980, *Bernardi 20273* (G).

GALACTIA GLAUCOPHYLLA

ARGENTINA. Córdoba: Uncertain department (Colón-Punilla): Sierra Chica, camino de Ascochinga a La Cumbre, 20.XI.1936, *A. Burkart 1298* (SI) – Calamuchita: Reartes, *Castellanos s.n.* (SI) – Río Cuarto: Near Achiras, no date, *Gillies s.n.* (SI) – Colón: Ascochinga, 09.XI.1936, *M. Giardelli 7364* (SI); Estancia La Reducción, 26.XII.1935, *A. Burkart 7346* (SI) – Punilla: Camino de Carlos Paz y Pampa de Achala, 12 km antres de Copina, 29.XII.1935, *A. I. Pastore 363* (SI) – Río Seco: En el camino entre Ojo de Agua y Villa María (km 959), ca. del límite con Santiago del Estero, 08.XI.1949, *A. T. Hunziker 8028* (SI) – Totoral: "San Jorge", Pedanía Río Quinto, I.1950, *M. Sayago 521B* (SI). **San Luis:** General Pedernera: Cerro Pelado, II.1938, *F. A. Pastore 2062* (SI); El Morro, 27.III.1905, *F. A. Pastore 50* (SI) – Pringles: El Durazno, I.1934, *F. A. Pastore 6623* (SI).

GALACTIA LATISILIQUA* VAR. *CHACOENSIS

BOLIVIA. Chuquisaca: Carandaity, 1935, *T. Rojas 12429* (LIL, SI).**PARAGUAY. Alto Paraguay:**Fuerte Olimpo, no date registered, *T. Rojas 13848* (LIL);Palmas Chicas, 1937, *T. Rojas 7644* (SI); Puerto Casado, no date registered,*J. Ramírez 39* (SI). **Central:** Luque, no date registered, *T. Rojas 12.429* (LIL, SI).**ARGENTINA. Catamarca:** El Alto: Sierra del Alto, Cruz Chiquita, I.1944, *A. Castellanos s.n.* (BAA47774).**Chaco:** Veinticinco de Mayo: Napalpí, no date, *F. Buratovich 35* (LIL). Córdoba: Río Seco: Sierras de Río Seco, entre Villa de María y San Miguel, 03.II.1949, *M. Sayago 290* (SI). **Formosa:** Patiño: Puesto Porteño, no date, *I. Morel 2477*(LIL). Pilcomayo: Puesto Porteño, no date, *I. Morel 2477* (LIL). **Jujuy:** Capital: Guaico Hondo, *L. Castillón 341-83* (LIL); Villa Achával, no date, *A. L. Cabrera & R. Kiesling 20054* (LIL). **Salta:** Caldera: Mojotoro, 17.XI.1942, *A. Burkart 13116* (SI). **Santiago del Estero:**

Guasayán: Cerrillos, serranías, 13.V.1983, *A. M. Molina et al. 1399* (BAB) – Silípica: Arraga, Arraga, campo del INTA, 12.v.1983, *A. M. Molina et al. 1293* (BAB). **Tucumán:** Chicligasta: Yacuchira, no date, *L. Monetti 1363* (LIL) – Leales: Tres Pozos, XII.1938, *Descole & Schreiter s.n.* (LIL68251).

INDIGOFERA KURTZII

ARGENTINA. Córdoba: Capital: Chacras de la Merced, Ruta nº 19, a 8 km de la ciudad (km 332), *L. Ariza Espinar 2441* (CORD) – Punilla: Capilla del Monte, 07.III.1940, *E. Nicora 2495* (SI).

INDIGOFERA PARODIANA

BOLIVIA.Santa Cruz: Chiquitos: 37 km W de San José de Chiquitos, camino a El Tinto, 08.VI.2006, *DeMatteis et al. 2311* (SI) – Manuel María Caballero: Saipina, Estancia Buena Vista, 6 km del pueblo, 02.I.1995, *J. Balcazar 85* (BAB). **PARAGUAY. Boquerón:** 17 km NE Trans-Chaco Highway in the road to Buena Vista, 17.II.2002, *M. Luckow et al. 4488* (BAB); at projecto Tagua, on the road to Fortín Toledo, 18.II.2002, *M. Luckow et al. 4494* (BAB); Between P. N. Teniente Agripino Enciso y Nueva Asunción, 27.I.1995, *E. M. Zardini & L. Guerrero 42458* (BAB); Parque Nacional Teniente Agripino Enciso, 14.XII.1998, *E. M. Zardini & N. Duarte 49881* (BAB).**ARGENTINA. Catamarca:** Valle Viejo: El Portezuelo, no date, *Inistroza s.n.* (LIL12036). **Córdoba:** Tercero Arriba: Pastizales en los alreddedores de la ciudad de Río Tercero, 15.III.1996, *R. H. Fortunato & A. Lamarque 5338* (BAB). **Jujuy:** Capital: Camino al aeropuerto El Cadillal, Arroyo Palo Marcado, 23.I.1976, *A. L. Cabrera et al. 27840* (BAB) – El Carmen: Alrededores de Pampa Blanca, 02.V.1992, *R. H. Fortunato et al. 3808* (BAB) – Perico: El Cadillal, 31.III.1977, *Krapovickas & Schinini 30787* (SI). **La Rioja**: Belgrano: Piedemonte oriental de la Sierra de Los Llanos, 10 km N de Olta, sobre R79, 23.I.1998, *F. Biurrun & E. Pagliari s.n.* (SI). **Salta:** Anta: Anta: Pozo Escondido, 49 km al E de J. V. González, 17.XII.1986, *Saravia Toledo 1257* (SI) – Capital: San Bernardo, *Spegazzini LPS11586* (LP) – Guachipas, 1 km al S de Guachipas en dirección a El Cebilar, 16.III.2001, *R. H. Fortunato et al. 7181* (BAB) – La Viña: La Viña, no date, *C. Spegazzini LPS11577* (LP)– San Martín: RN81, 30 km E de Dragones, 08.V.1999, *Seijo & Krapovickas 1951* (BAB). **Santiago del Estero:** Guasayán: El Cebilar, III.1944, *Pierotti s.n.* (SI) –Quebrachos: Sumampa Viejo, 27.X.1946,

27.X.1946, *Ragonese 6305* (BAB). **Tucumán:** Capital: Duraznito, 03.II.1922, *S. Venturi 1678* (SI) – Trancas: ±200 m antes de Vipos, 30.III.1971, *M. N. Correa et al. 4223* (BAB).

STYLOSANTHES RECTA

PARAGUAY. Boquerón: 9 km al E de Loma Plata, propiedad del Sr. Ebenfeld, *Vanbni et al., 2173* (CTES); Estación Experimental Neuland, *Vanni 2495* (ctes); Estancia San Ramón, *L. Pérez 2531* (CTES): Parque Valle Natural, *Vanni 2448* (CTES, G, LIL, NY).**Presidente Hayes:** Estancia Yrenda, *L. Pérez 2995* (CTES); Isla Poí, 24 km N de ruta Transchaco, km 415, *Krapovickas 44356* (CTES).

TEPHROSIA HASSLERI

PARAGUAY. Cordillera: Tobaty IX.1900, *Hassler 6156* (G). **ARGENTINA. Corrientes:** San Miguel: Estancia San Juan Poriajhu, ruta 17, 8 km S de Ruta 12, 06.XII.1992, *S. Tressens et al. 4247* (CTES).
