# Supplementary material for: Diversity and conservation of legumes in the Gran Chaco and biogeograpical inferences
Source: PLoS One. 2019 Aug 14;14(8):e0220151. doi: 10.1371/journal.pone.0220151 (PMC6693842; doi:10.1371/journal.pone.0220151)
Supplement: S1 Table — IUCN Categorization—Criteria and Parameters. (PDF) [file pone.0220151.s002.pdf]

S1 Table. IUCN Categorization – criteria and parameters

Subfamily	Genus	Specific epithet	Subspecies	Variety	Form	EEO	Category GEOCAT	AAO	Grid siz3e	Category GEOCAT	Severely fragmented/few populations	Continuing decline	Extreme fluctuations	Decision
Caesalpinioideae	Acacia	caven		microcarpa		82514,419	LC	192000	4	EN	no	no	no	LC
Caesalpinioideae	Acacia	curvifructa				315702,045	LC	416000	4	EN	no	no	no	LC
Caesalpinioideae	Acacia	emilioana				110888,334	LC	208000	4	EN	no	no	no	VU
Caesalpinioideae	Acacia	monacantha			schulziana						Unknown	Unknown	Unknown	DD
Caesalpinioideae	Chamaecrista	arachyphylla				44.038.132	NT	96000	4	EN	yes	no	no	EN
Caesalpinioideae	Chloroleucon	chacöense				239257,898	LC	192000	4	EN	yes	no	no	VU
Caesalpinioideae	Denisophytum	stuckerti				239257,898	LC	48000	4	EN	no	no	no	VU
Caesalpinioideae	Desmanthus	tatuhyensis		brevipes		162736,772	LC	132000	2	EN	no	no	no	LC
Caesalpinioideae	Erythrostemon	argentinus				310021,672	LC	464000	4	EN	yes	no	no	EN
Caesalpinioideae	Erythrostemon	coluteifolius				825626,377	LC	544000	4	VU	no	no	no	VU
Caesalpinioideae	**Libidibia**	paraguariensis				614328,676	LC	688000	4	VU	no	no	no	LC
Caesalpinioideae	Lophocarpinia	aculeatifolia				132559,697	LC	496000	4	EN	no	no	no*	VU
Caesalpinioideae	Mimosa	castanoclada				56133,786	LC	272000	4	EN	no	yes	yes by deforestation	EN
Caesalpinioideae	Mimosa	centurionis				113826	EN	3000	1	CR	yes	Unknown	yes, potential change of land use	CR
Caesalpinioideae	Mimosa	chacöensis				191360,37	LC	44000	2	EN	no	yes	yes, deforestation and build of roads	EN
Caesalpinioideae	Mimosa	cordobensis				254264.264	EN	3000	1	CR	yes	Unknown	yes, area affected by tourism and urban expansion	CR

Caesalpinioideae	Mimosa	craspedisetosa		17925,138	VU	64000	4	EN	yes	Unknown	yes, potential change of land use	EN
Caesalpinioideae	Mimosa	detinens		641320,144	LC	720000	4	VU	no	no	no	LC
Caesalpinioideae	Mimosa	morongii		189112	EN	4000	1	CR	yes	Unknown	yes, drastic urban expansion	CR
Caesalpinioideae	Mimosa	pseudopetiolaris		1639,241	EN	24000	2	EN	yes	yes, 1 population is possible missing	yes, drastic urban expansion	EN
Caesalpinioideae	Mimosa	sensibilis	sensibilis	355372,809	LC	880000	4	VU	no	no	no	LC
Caesalpinioideae	Mimosa	tobatiensis		11108,412	VU	16000	2	EN	yes	yes, 1 population is possible missing	yes, at least one population threatened	EN
Caesalpinioideae	Mimosa	troncosoae			CR			CR	yes	N/D	yes, drastic changes in land use, deforestation	CR
Caesalpinioideae	Mimozyganthus	carinatus		753529,861	LC	1152000	4	VU	no	no	no	LC
Caesalpinioideae	Piptadeniopsis	lomentifera		92004,856	LC	256000	4	EN	no	yes	yes, deforestation and drastic changes in land use	EN
Caesalpinioideae	Prosopis	campestris		54951,001	LC	20000	2	EN	yes	Unknown	yes, high rates of deforestation	EN
Caesalpinioideae	Prosopis	elata		185676,473	LC	272000	4	EN	no	no	no	LC
Caesalpinioideae	Prosopis	fiebrigii		132953,304	LC	208000	4	EN	no	no	no	LC
Caesalpinioideae	Prosopis	hassleri	hassleri	202811521	LC	400000	4	EN	no	no	no	LC
Caesalpinioideae	Prosopis	hassleri	nigroides						yes	Unknown	yes, deforestation	DD
Caesalpinioideae	Prosopis	kuntzei		1094353,42	LC	1776000	4	VU	no	no	no	LC

Caesalpinioideae	Prosopis	nigra	ragonesei	-	-	32000	4	EN	yes, but big populations were found by the authors and they are not yet adequately documented in herbaria.	no	no	VU
Caesalpinioideae	Prosopis	nigra	longispina	-	CR	32000	4	EN	yes, but big populations were found by the authors and they are not yet adequately documented in herbaria.	N/D	Yes, some populations in suburban areas	VU
Caesalpinioideae	Prosopis	nuda		77070,779	LC	176000	4	EN	no	no	no	VU
Caesalpinioideae	Prosopis	pugionata		73884,116	LC	224000	4	EN	no	no	no	LC
Caesalpinioideae	Prosopis	rojasiana		50699,485	LC	128000	4	EN	yes	Unknown	Yes, deforestation	EN
Caesalpinioideae	Prosopis	rubriflora		5560,057	VU	80000	4	EN	yes	yes	Yes, deforestation	EN
Caesalpinioideae	Prosopis	vinalillo		**584.319,89**	LC	560000	4	VU	no	no	no	LC

Caesalpinioideae	Senna	chacoënsis		241271,343	LC	192000	4	EN	no	no	no	LC
Caesalpinioideae	Senna	chloroclada		403477,951	LC	752000	4	VU	no	no	no	LC
Caesalpinioideae	Senna	spiniflora							no	no	no	LC
Cercidoideae	Bauhinia	argentinensis	megasiphon	21213,896	LC	48000	4	EN	yes	Unknown	yes, at least one population in an area with high deforestation rate	EN
Cercidoideae	Bauhinia	argentinensis	argentinensis	298742,85	LC	208000	4	EN	no	no	no	LC
Cercidoideae	Bauhinia	hagenbeckii		132886,59	LC	208000	4	EN	no	no	no	NT
Papilionoideae	Adesmia	cordobensis		114533,903	LC	124000	2	EN	no	no	no	LC
Papilionoideae	Aeschynomene	paraguayensis		-	CR	8000	2	CR	yes	Unknown	yes, population in suburban areas	CR
Papilionoideae	Apurimacia	dolichocarpa		22499	CR	4000	1	CR	yes	Unknown	yes, at least 2 localities in turistic areas	CR
Papilionoideae	Arachis	batizocoi		18136,424	VU	9000	1	CR	yes	Unknown	yes, by construction of gasoducts and roads and invasive plants	CR
Papilionoideae	Arachis	correntina		71980,396	LC	26000	1	EN	no	no	no	LC
Papilionoideae	Arachis	duranensis		65117,797	LC	23000	1	EN	no	no	no	EN
Papilionoideae	Arachis	hassleri		8154	CR	2000	1	CR	yes	no	yes, by deforestation and changes in land use	CR
Papilionoideae	Arachis	lignosa		2508,714	EN	5000	1	CR	yes	Unknown	yes, populations in suburban areas	CR
Papilionoideae	Arachis	microsperma		-	CR	2000	1	CR	yes	Unknown	yes, by deforestation and changes in land use	CR
Papilionoideae	Centrosema	kermesi			DD			DD	-	-	-	DD
Papilionoideae	Chaetocalyx	chacoensis		25175,444	NT	24000	2	EN	yes	Unknown	yes, high rates of deforestation	EN
Papilionoideae	Crotalaria	chaco-serranensis		220405,426	LC	640000	4	VU	no	no	no	LC

Papilionoideae	Dalea	elegans		162736,772	LC	132000	2	EN	no	no	no	LC
Papilionoideae	Desmodium	burkartii		-	CR	8000	2	CR	yes	Unknown	yes, at least one population in suburban areas	CR
Papilionoideae	Desmodium	intermedium		-	CR	8000	2	CR	yes	Unknown	yes, by overgrazing and plantation of pines	CR
Papilionoideae	Galactia	glaucophylla		20930,058	NT	44000	2	EN	no	Unknown	no	VU
Papilionoideae	Galactia	latisiliqua	chacoensis	**378.663,95**	LC	44000	2	EN	no	no	no	LC
Papilionoideae	Galactia	texana	degasperii	13107,385	VU	12000	2	EN	yes	Unknown	yes, high rates of deforestation	EN
Papilionoideae	Indigofera	kurtzii		11163,256	VU	20000	2	EN	5 localidades	One population (Catamarca) with few modern collections	yes, by expansion of turistic activities	EN
Papilionoideae	Indigofera	parodiana		616217,671	LC	100000	2	EN	no	no	no	LC
Papilionoideae	Stylosanthes	recta		12722,028	VU	24000	2	EN	yes	Unknown	yes, growing in an area with intensive agriculture	EN
Papilionoideae	Tephrosia	hassleri		-	CR	8000	2	CR	yes	Unknown	yes, by urban expansion and minery	CR
